# Supplementary material for: Surgical intervention for paediatric infusion-related extravasation injury: a systematic review
Source: BMJ Open. 2020 Aug 6;10(8):e034950. doi: 10.1136/bmjopen-2019-034950 (PMC7412604; doi:10.1136/bmjopen-2019-034950)
Supplement: Supplementary data [file bmjopen-2019-034950supp001.pdf]

Evidence Services | library.nhs.uk

Search Results

Table of Contents

Search History .... page 2

1. Thoracic and abdominal extravasation: a complication of hyperalimentation in infants. .... page 4

2. [Traumatic cerebral aneurysm showing remarkable extravasation into the lateral ventricle--report of a case in an infant (author's transl)]. .... page 4

3. Urinary ascites in children owing to extravasation of urine from the bladder. .... page 4

4. Bone scintigraphy for neonatal osteomyelitis: simulation by extravasation of intravenous calcium. .... page 4

5. Perirenal urinary extravasation associated with urethral valves in infants. .... page 4

6. Nafcillin extravasation injury. Use of hyaluronidase as an antidote. .... page 4

7. Cutaneous calcinosis in a neonate following extravasation of calcium gluconate. .... page 4

8. Urinary extravasation in the newborn male with posterior urethral valves. .... page 4

9. Intrathoracic extravasation of sclerosing agents associated with central venous catheters. .... page 5

10. Nonoperative management of full-thickness intravenous extravasation injuries in premature neonates using enzymatic debridement. .... page 5

Little M, *et al. BMJ Open* 2020; 10:e034950. doi: 10.1136/bmjopen-2019-034950

Search History

1. AMED, EMBASE, Medline, CINAHL; p?ediatric*.ti,ab; 2702402 results.
2. AMED, EMBASE, Medline, CINAHL; neonat*.ti,ab; 508216 results.
3. AMED, EMBASE, Medline, CINAHL; infant*.ti,ab; 761110 results.
4. AMED, EMBASE, Medline, CINAHL; child.ti,ab; 737276 results.
5. AMED, EMBASE, Medline, CINAHL; 1 OR 2 OR 3 OR 4; 4209881 results.
6. AMED, EMBASE, Medline, CINAHL; extra?vasat*.ti,ab; 19189 results.
7. AMED, EMBASE, Medline, CINAHL; extravasation.ti,ab; 30514 results.
8. AMED, EMBASE, Medline, CINAHL; 6 OR 7; 32931 results.
9. AMED, EMBASE, Medline, CINAHL; 5 AND 8; 1849 results.
10. AMED, EMBASE, Medline, CINAHL; outcome*.ti,ab; 2946939 results.
11. AMED, EMBASE, Medline, CINAHL; PROM*.ti,ab; 2921492 results.
12. AMED, EMBASE, Medline, CINAHL; patient?related*.ti,ab; 131 results.
13. AMED, EMBASE, Medline, CINAHL; patient?report*.ti,ab; 689 results.
14. AMED, EMBASE, Medline, CINAHL; 10 OR 11 OR 12 OR 13; 5654365 results.
15. AMED, EMBASE, Medline, CINAHL; conservative*.ti,ab; 231035 results.
16. AMED, EMBASE, Medline, CINAHL; non?operative*.ti,ab; 11082 results.
17. AMED, EMBASE, Medline, CINAHL; non?surg*.ti,ab; 14737 results.
18. AMED, EMBASE, Medline, CINAHL; cold*.ti,ab; 225847 results.
19. AMED, EMBASE, Medline, CINAHL; cool*.ti,ab; 101309 results.
20. AMED, EMBASE, Medline, CINAHL; hot*.ti,ab; 160999 results.
21. AMED, EMBASE, Medline, CINAHL; warm*.ti,ab; 110969 results.
22. AMED, EMBASE, Medline, CINAHL; elevat*.ti,ab; 1359688 results.
23. AMED, EMBASE, Medline, CINAHL; dress*.ti,ab; 53707 results.
24. AMED, EMBASE, Medline, CINAHL; 15 OR 16 OR 17 OR 18 OR 19 OR 20 OR 21 OR 22 OR 23; 2170626 results.
25. AMED, EMBASE, Medline, CINAHL; medic*.ti,ab; 3892625 results.
26. AMED, EMBASE, Medline, CINAHL; steroid*.ti,ab; 449723 results.
27. AMED, EMBASE, Medline, CINAHL; cortico?steroid*.ti,ab; 114994 results.
28. AMED, EMBASE, Medline, CINAHL; corticosteroid*.ti,ab; 206312 results.
29. AMED, EMBASE, Medline, CINAHL; sub?cut*.ti,ab; 245741 results.
30. AMED, EMBASE, Medline, CINAHL; subcut*.ti,ab; 189955 results.
31. AMED, EMBASE, Medline, CINAHL; inject*.ti,ab; 1404121 results.
32. AMED, EMBASE, Medline, CINAHL; topical*.ti,ab; 199318 results.
33. AMED, EMBASE, Medline, CINAHL; cream*.ti,ab; 38332 results.
34. AMED, EMBASE, Medline, CINAHL; ointment*.ti,ab; 24526 results.
35. AMED, EMBASE, Medline, CINAHL; emollient*.ti,ab; 3696 results.
36. AMED, EMBASE, Medline, CINAHL; antidote*.ti,ab; 11910 results.
37. AMED, EMBASE, Medline, CINAHL; Sodium?thiosulfate*.ti,ab; 6 results.
38. AMED, EMBASE, Medline, CINAHL; mechlorethamine*.ti,ab; 1486 results.
39. AMED, EMBASE, Medline, CINAHL; Dexrazoxane*.ti,ab; 1175 results.
40. AMED, EMBASE, Medline, CINAHL; N?acetylcysteine*.ti,ab; 1604942 results.
41. AMED, EMBASE, Medline, CINAHL; vitamin?C*.ti,ab; 422 results.
42. AMED, EMBASE, Medline, CINAHL; ascorbic*.ti,ab; 58559 results.
43. AMED, EMBASE, Medline, CINAHL; basic AND fibroblast AND growth AND factor.ti,ab; 29979 results.
44. AMED, EMBASE, Medline, CINAHL; granulocyte AND macrophage AND colony AND stimulating AND factor.ti,ab; 37389 results.
45. AMED, EMBASE, Medline, CINAHL; hyaluroni*.ti,ab; 42841 results.
46. AMED, EMBASE, Medline, CINAHL; salin*.ti,ab; 375104 results.
47. AMED, EMBASE, Medline, CINAHL; 25 OR 26 OR 27 OR 28 OR 29 OR 30 OR 31 OR 32 OR 33 OR 34 OR 35 OR 36 OR 37 OR 38 OR 39 OR 40 OR 41 OR 42 OR 43 OR 44 OR 45 OR 46; 7833762 results.
48. AMED, EMBASE, Medline, CINAHL; squeeze*.ti,ab; 9290 results.
49. AMED, EMBASE, Medline, CINAHL; surg*.ti,ab; 3592311 results.
50. AMED, EMBASE, Medline, CINAHL; intervention*.ti,ab; 1719913 results.
51. AMED, EMBASE, Medline, CINAHL; irrigation*.ti,ab; 45442 results.
52. AMED, EMBASE, Medline, CINAHL; excis*.ti,ab; 326226 results.
53. AMED, EMBASE, Medline, CINAHL; infiltrat*.ti,ab; 404118 results.
54. AMED, EMBASE, Medline, CINAHL; therapeut*.ti,ab; 1901755 results.
55. AMED, EMBASE, Medline, CINAHL; manage*.ti,ab; 2431717 results.
56. AMED, EMBASE, Medline, CINAHL; treatment*.ti,ab; 8388485 results.

57. AMED, EMBASE, Medline, CINAHL; wash?out*.ti,ab; 28666 results.
58. AMED, EMBASE, Medline, CINAHL; operat*.ti,ab; 2000359 results.
59. AMED, EMBASE, Medline, CINAHL; bedside*.ti,ab; 57042 results.
60. AMED, EMBASE, Medline, CINAHL; liposuct*.ti,ab; 5719 results.
61. AMED, EMBASE, Medline, CINAHL; fasciotom*.ti,ab; 5310 results.
62. AMED, EMBASE, Medline, CINAHL; 48 OR 49 OR 50 OR 51 OR 52 OR 53 OR 54 OR 55 OR 56 OR 57 OR 58 OR 59 OR 60 OR 61; 15163731 results.
63. AMED, EMBASE, Medline, CINAHL; 9 AND 24; 222 results.
64. AMED, EMBASE, Medline, CINAHL; 9 AND 47; 982 results.
65. AMED, EMBASE, Medline, CINAHL; 9 AND 62; 1130 results.
66. AMED, EMBASE, Medline, CINAHL; 24 OR 47 OR 62; 20658308 results.
67. AMED, EMBASE, Medline, CINAHL; 9 AND 66; 1455 results.
68. AMED, EMBASE, Medline, CINAHL; 67 NOT animal; 1176 results.
69. AMED, EMBASE, Medline, CINAHL; 68 NOT laboratory; 1117 results.
70. AMED, EMBASE, Medline, CINAHL; 69 NOT adult; 804 results.

1. Thoracic and abdominal extravasation: a complication of hyperalimentation in infants.

Citation: AJR. American journal of roentgenology, Mar 1977, vol. 128, no. 3, p. 419-422, 0361-803X (March 1977)

Author(s): Spriggs, D W; Brantley, R E

Source: Medline

2. [Traumatic cerebral aneurysm showing remarkable extravasation into the lateral ventricle--report of a case in an infant (author's transl)].

Citation: No shinkei geka. Neurological surgery, Apr 1977, vol. 5, no. 4, p. 371-378, 0301-2603 (April 1977)

Author(s): Nakamura, T; Matsuoka, Y; Nishimura, S

Source: Medline

3. Urinary ascites in children owing to extravasation of urine from the bladder.

Citation: The Journal of urology, Sep 1979, vol. 122, no. 3, p. 409-411, 0022-5347 (September 1979)

Author(s): Redman, J F; Seibert, J J; Arnold, W

Source: Medline

4. Bone scintigraphy for neonatal osteomyelitis: simulation by extravasation of intravenous calcium.

Citation: Radiology, Apr 1980, vol. 135, no. 1, p. 185-186, 0033-8419 (April 1980)

Author(s): Balsam, D; Goldfarb, C R; Stringer, B; Farruggia, S

Source: Medline

5. Perirenal urinary extravasation associated with urethral valves in infants.

Citation: The Journal of urology, Nov 1980, vol. 124, no. 5, p. 688-691, 0022-5347 (November 1980)

Author(s): Mitchell, M E; Garrett, R A

Source: Medline

6. Nafcillin extravasation injury. Use of hyaluronidase as an antidote.

Citation: American journal of diseases of children (1960), Dec 1981, vol. 135, no. 12, p. 1113-1114, 0002-922X (December 1981)

Author(s): Zenk, K E; Dungy, C I; Greene, G R

Source: Medline

7. Cutaneous calcinosis in a neonate following extravasation of calcium gluconate.

Citation: Journal of the American Academy of Dermatology, Mar 1982, vol. 6, no. 3, p. 392-395, 0190-9622 (March 1982)

Author(s): Hironaga, M; Fujigaki, T; Tanaka, S

Source: Medline

8. Urinary extravasation in the newborn male with posterior urethral valves.

Citation: Journal of pediatric surgery, Dec 1982, vol. 17, no. 6, p. 751-756, 0022-3468 (December 1982)

Author(s): Greenfield, S P; Hensle, T W; Berdon, W E; Geringer, A M

Source: Medline

9. Intrathoracic extravasation of sclerosing agents associated with central venous catheters.

Citation: The American journal of pediatric hematology/oncology, Jan 1988, vol. 10, no. 3, p. 249-251, 0192-8562 (1988)

Author(s): Watterson, J; Heisel, M; Cich, J A; Priest, J R

Source: Medline

Full Text: Available from *Ovid* in *American Journal of Pediatric Hematology/Oncology*

10. Nonoperative management of full-thickness intravenous extravasation injuries in premature neonates using enzymatic debridement.

Citation: Annals of plastic surgery, Feb 1989, vol. 22, no. 2, p. 146-149, 0148-7043 (February 1989)

Author(s): Falcone, P A; Barrall, D T; Jeyarajah, D R; Grossman, J A

Source: Medline

Full Text: Available from *Ovid* in *Annals of Plastic Surgery*

Evidence Services | library.nhs.uk

Search Results

Table of Contents

Search History .... page 2

1. Hepatic and splenic blush on computed tomography in children following blunt abdominal trauma: Is intervention necessary? .... page 3

2. Safety and effectiveness of intravenous pentamidine for prophylaxis of Pneumocystis jirovecii pneumonia in pediatric hematology/oncology patients .... page 3

3. Efficacy, Safety, and Pharmacokinetics of a New 10 % Liquid Intravenous Immunoglobulin Containing High Titer Neutralizing Antibody to RSV and Other Respiratory Viruses in Subjects with Primary Immunodeficiency Disease .... page 3

4. A multicenter, randomized, observer-blinded, active-controlled study evaluating the safety and effectiveness of ceftaroline compared with ceftriaxone plus vancomycin in pediatric patients with complicated community-acquired bacterial pneumonia .... page 3

5. Predictive factors for conservative treatment failure in grade IV pediatric blunt renal trauma .... page 3

6. Clinical negligence claims in pediatric surgery in England: Pattern and trends .... page 3

7. Outcomes of peripherally inserted double lumen central catheter in very low birth weight infants .... page 4

8. Pelvic Artery Embolization in the Management of Obstetrical Hemorrhage: Predictive Factors for Clinical Outcomes .... page 4

9. The role of interventional radiology for pediatric blunt renal trauma .... page 4

10. Safety and efficacy of gadoteric acid in pediatric magnetic resonance imaging: overview of clinical trials and post-marketing studies .... page 4

Little M, *et al. BMJ Open* 2020; 10:e034950. doi: 10.1136/bmjopen-2019-034950

Search History

1. EMBASE; exp PEDIATRICS/; 87850 results.
2. EMBASE; exp CHILD/; 2256483 results.
3. EMBASE; exp INFANT/; 930470 results.
4. EMBASE; exp ADOLESCENT/; 1367630 results.
5. EMBASE; exp NEWBORN/; 495069 results.
6. EMBASE; exp CONTRAST MEDIUM EXTRAVASATION/ OR exp DRUG EXTRAVASATION/ OR exp EXTRAVASATION/ OR exp INJECTION SITE EXTRAVASATION/; 11979 results.
7. EMBASE; exp OUTCOME MEASUREMENT/ OR exp OUTCOMES RESEARCH/ OR exp EVIDENCE BASED MEDICINE/ OR exp TREATMENT OUTCOME/; 1809683 results.
8. EMBASE; 1 OR 2 OR 3 OR 4 OR 5; 2973897 results.
9. EMBASE; 6 AND 8; 958 results.
10. EMBASE; 7 AND 9; 150 results.

1. Hepatic and splenic blush on computed tomography in children following blunt abdominal trauma: Is intervention necessary?

Citation: Journal of Trauma and Acute Care Surgery, 2016, vol./is. 81/2(266-270), 2163-0755;2163-0763 (2016)

Author(s): Ingram M.-C.E.; Siddharthan R.V.; Morris A.D.; Hill S.J.; Travers C.D.; McKracken C.E.; Heiss K.F.; Raval M.V.; Santore M.T.

Source: EMBASE

Full Text: Available from *Ovid* in *Journal of Trauma and Acute Care Surgery*

2. Safety and effectiveness of intravenous pentamidine for prophylaxis of Pneumocystis jirovecii pneumonia in pediatric hematology/oncology patients

Citation: Journal of Pediatric Hematology/Oncology, July 2016, vol./is. 38/6(e180-e185), 1077-4114;1536-3678 (26 Jul 2016)

Author(s): Solodokin L.J.; Klejmont L.M.; Scipione M.R.; Dubrovskaya Y.; Lighter-Fisher J.; Papadopoulos J.

Source: EMBASE

Full Text: Available from *Ovid* in *Journal of Pediatric Hematology/Oncology*

3. Efficacy, Safety, and Pharmacokinetics of a New 10 % Liquid Intravenous Immunoglobulin Containing High Titer Neutralizing Antibody to RSV and Other Respiratory Viruses in Subjects with Primary Immunodeficiency Disease

Citation: Journal of Clinical Immunology, August 2016, vol./is. 36/6(590-599), 0271-9142;1573-2592 (01 Aug 2016)

Author(s): Wasserman R.L.; Lumry W.; Harris J.; Levy R.; Stein M.; Forbes L.; Cunningham-Rundles C.; Melamed I.; Kobayashi A.L.; Du W.; Kobayashi R.

Source: EMBASE

Full Text: Available from *Springer Link Journals* in *Journal of Clinical Immunology*

4. A multicenter, randomized, observer-blinded, active-controlled study evaluating the safety and effectiveness of ceftaroline compared with ceftriaxone plus vancomycin in pediatric patients with complicated community-acquired bacterial pneumonia

Citation: Pediatric Infectious Disease Journal, July 2016, vol./is. 35/7(760-766), 0891-3668;1532-0987 (01 Jul 2016)

Author(s): Blumer J.L.; Ghonghadze T.; Cannavino C.; O'Neal T.; Jandourek A.; Friedland H.D.; Bradley J.S.

Source: EMBASE

Full Text: Available from *Ingenta* in *Pediatric Infectious Disease Journal, The*
Available from *Ovid* in *Pediatric Infectious Disease Journal*

5. Predictive factors for conservative treatment failure in grade IV pediatric blunt renal trauma

Citation: Journal of Pediatric Urology, April 2016, vol./is. 12/2(93.e1-93.e7), 1477-5131;1873-4898 (01 Apr 2016)

Author(s): Lee J.N.; Lim J.K.; Woo M.J.; Kwon S.Y.; Kim B.S.; Kim H.T.; Kim T.-H.; Yoo E.S.; Chung S.K.

Source: EMBASE

6. Clinical negligence claims in pediatric surgery in England: Pattern and trends

Citation: European Journal of Pediatric Surgery, February 2015, vol./is. 25/1(66-70), 0939-7248;1439-359X (February 2015)

Author(s): Thyoka M.

Source: EMBASE

7. Outcomes of peripherally inserted double lumen central catheter in very low birth weight infants

Citation: Journal of Neonatal-Perinatal Medicine, March 2016, vol./is. 9/1(99-105), 1934-5798;1878-4429 (24 Mar 2016)

Author(s): Cheong S.M.; Totsu S.; Nakanishi H.; Uchiyama A.; Kusuda S.

Source: EMBASE

8. Pelvic Artery Embolization in the Management of Obstetrical Hemorrhage: Predictive Factors for Clinical Outcomes

Citation: CardioVascular and Interventional Radiology, December 2015, vol./is. 38/6(1477-1486), 0174-1551;1432-086X (01 Dec 2015)

Author(s): Zhang E.; Liu L.; Owen R.

Source: EMBASE

Full Text: Available from *Springer Link Journals* in *CardioVascular and Interventional Radiology*

9. The role of interventional radiology for pediatric blunt renal trauma

Citation: Italian Journal of Pediatrics, October 2015, vol./is. 41/1(no pagination), 1720-8424;1824-7288 (15 Oct 2015)

Author(s): Lin W.-C.; Lin C.-H.

Source: EMBASE

Full Text: Available from *National Library of Medicine* in *Italian Journal of Pediatrics*
Available from *BioMed Central* in *Italian Journal of Pediatrics*
Available from *National Library of Medicine* in *Italian Journal of Pediatrics*
Available from *ProQuest* in *Italian Journal of Pediatrics*

10. Safety and efficacy of gadoteric acid in pediatric magnetic resonance imaging: overview of clinical trials and post-marketing studies

Citation: Pediatric Radiology, November 2015, vol./is. 45/12(1831-1841), 0301-0449;1432-1998 (01 Nov 2015)

Author(s): Balassy C.; Roberts D.; Miller S.F.

Source: EMBASE

Full Text: Available from *Springer Link Journals* in *Pediatric Radiology*

Evidence Services | library.nhs.uk

Search Results

Table of Contents

Search History .... page 2

1. Emergency treatment of accidental infusion leakage in the newborn. .... page 3

2. Childhood extravasation injuries: improved outcome following the introduction of hospital-wide guidelines. .... page 3

3. [Treatment protocol for extravasation lesions]. .... page 3

4. The use of central venous catheters in paediatric oncology--a cautionary tale. .... page 3

5. A new approach to management of intravenous infiltration in pediatric patients: pathophysiology, classification, and treatment. .... page 3

6. Saline irrigation for the management of skin extravasation injury in neonates. .... page 3

7. Extravasation injuries. .... page 3

8. [Central venous catheters in newborn infants. Work Group Parenteral Nutrition of the Perinatology Section of the Dutch Pediatric Society]. .... page 4

9. Extravasation of parenteral alimentation fluid into the renal pelvis--a complication of central venous catheter in a neonate. .... page 4

10. Percutaneous central line extravasation masquerading as an abscess. .... page 4

Little M, *et al. BMJ Open* 2020; 10:e034950. doi: 10.1136/bmjopen-2019-034950

Search History

1. Medline; exp PEDIATRICS/; 49428 results.
2. Medline; exp CHILD/; 1666146 results.
3. Medline; exp INFANT/; 1008023 results.
4. Medline; exp INFANT, NEWBORN/; 535610 results.
5. Medline; 1 OR 2 OR 3 OR 4; 2185064 results.
6. Medline; exp EXTRAVASATION OF DIAGNOSTIC AND THERAPEUTIC MATERIALS/; 3098 results.
7. Medline; exp "OUTCOME ASSESSMENT (HEALTH CARE)"/ OR exp PATIENT OUTCOME ASSESSMENT/ OR exp TREATMENT OUTCOME/; 815946 results.
8. Medline; exp EARLY MEDICAL INTERVENTION/; 1559 results.
9. Medline; exp THERAPEUTIC IRRIGATION/ OR exp THERAPEUTICS/; 5011881 results.
10. Medline; exp SPECIALTIES, SURGICAL/ OR exp SURGICAL PROCEDURES, OPERATIVE/; 2809893 results.
11. Medline; 8 OR 9 OR 10; 6354238 results.
12. Medline; 5 AND 6; 345 results.
13. Medline; 7 AND 12; 36 results.
14. Medline; 11 AND 12; 248 results.
15. Medline; 12 OR 13 OR 14; 345 results.

1. Emergency treatment of accidental infusion leakage in the newborn.

Citation: British journal of plastic surgery, Jan 2002, vol. 55, no. 1, p. 89., 0007-1226 (January 2002)

Author(s): Rose, Victoria; Turner, Matthew; Harris, Paul A; Moss, Anthony L H

Source: Medline

Full Text: Available from *Ovid* in *Plastic and Reconstructive Surgery*

2. Childhood extravasation injuries: improved outcome following the introduction of hospital-wide guidelines.

Citation: Journal of plastic, reconstructive & aesthetic surgery : JPRAS, Apr 2015, vol. 68, no. 4, p. 505-518, 1878-0539 (April 2015)

Author(s): Ghanem, Ali M; Mansour, Abdulrab; Exton, Rebecca; Powell, Jonathan; Mashhadi, Syed; Bulstrode, Neil; Smith, Gillian

Source: Medline

3. [Treatment protocol for extravasation lesions].

Citation: Cirugía pediátrica : organo oficial de la Sociedad Española de Cirugía Pediátrica, Jul 2006, vol. 19, no. 3, p. 136-139, 0214-1221 (July 2006)

Author(s): Andrés, A M; Burgos, L; López Gutiérrez, J C; Encinas, J L; Díaz, M; Rivas, S; Ros, Z

Source: Medline

4. The use of central venous catheters in paediatric oncology--a cautionary tale.

Citation: Scottish medical journal, Feb 1990, vol. 35, no. 1, p. 11-14, 0036-9330 (February 1990)

Author(s): Russell, L; Craig, J I; Mackinlay, G A; Eden, O B

Source: Medline

5. A new approach to management of intravenous infiltration in pediatric patients: pathophysiology, classification, and treatment.

Citation: Journal of infusion nursing : the official publication of the Infusion Nurses Society, Jul 2011, vol. 34, no. 4, p. 242-249, 1539-0667 (2011 Jul-Aug)

Author(s): Amjad, Ibrahim; Murphy, Travis; Nylander-Housholder, Linda; Ranft, Amanda

Source: Medline

Full Text: Available from *Ovid* in *Journal of Infusion Nursing*

6. Saline irrigation for the management of skin extravasation injury in neonates.

Citation: The Cochrane database of systematic reviews, Jan 2012, no. 2, p. CD008404., 1469-493X (2012)

Author(s): Gopalakrishnan, P N; Goel, N; Banerjee, Sujoy

Source: Medline

Full Text: Available from *John Wiley and Sons* in *Cochrane Library, The*

7. Extravasation injuries.

Citation: British journal of plastic surgery, Mar 1993, vol. 46, no. 2, p. 91-96, 0007-1226 (March 1993)

Author(s): Gault, D T

Source: Medline

Full Text: Available from *Ovid* in *Plastic and Reconstructive Surgery*

8. [Central venous catheters in newborn infants. Work Group Parenteral Nutrition of the Perinatology Section of the Dutch Pediatric Society].

**Citation:**	Tijdschrift voor kindergeneeskunde, Jun 1993, vol. 61, no. 3, p. 76-82, 0376-7442 (June 1993)
**Author(s):**	van Lingen, R A; Liem, K D; Krediet, T G
**Source:**	Medline

9. Extravasation of parenteral alimentation fluid into the renal pelvis--a complication of central venous catheter in a neonate.

**Citation:**	Journal of perinatology : official journal of the California Perinatal Association, Oct 2001, vol. 21, no. 7, p. 465-466, 0743-8346 (2001 Oct-Nov)
**Author(s):**	Nadroo, A M; al-Sowailem, A M
**Source:**	Medline
**Full Text:**	Available from *Nature Publishing Group* in *Journal of Perinatology*<br>Available from *Nature Publishing Group* in *Journal of Perinatology*<br>Available from *ProQuest* in *Journal of Perinatology*

10. Percutaneous central line extravasation masquerading as an abscess.

**Citation:**	Indian pediatrics, Apr 2014, vol. 51, no. 4, p. 309-310, 0974-7559 (April 2014)
**Author(s):**	Govind, Binu; Tete, Prakash Ignace; Thomas, Niranjan
**Source:**	Medline
**Full Text:**	Available from *Springer Link Journals* in *Indian Pediatrics*
