# Supplementary material for: Surgical intervention for paediatric infusion-related extravasation injury: a systematic review
Source: BMJ Open. 2020 Aug 6;10(8):e034950. doi: 10.1136/bmjopen-2019-034950 (PMC7412604; doi:10.1136/bmjopen-2019-034950)
Supplement: Supplementary data [file bmjopen-2019-034950supp002.pdf]

Supplementary 2
Study Attrition Process

Level 1 - Is a clinical study of paediatric extravasation injury?

If yes then proceed to level 2

Level 2 - Does it report on one or more of the following outcomes?

- Short-term outcomes:
    - Time to complete tissue healing
    - Infection
    - Hypothermia
    - Anaphylactic reactions
    - Fluid and electrolyte disturbance
    - Any other adverse effects reported in the studies;
- Long-term outcomes (preferably at least three months from the injury):
    - Severity of the scar including contractures, functional impairment, disfigurement and need for further surgical procedures.

Level 3 – is it an original clinical study or systematic review of clinical studies?

If yes then proceed to level 4

Level 4 - In which of the following groups of the study types does it belong?

Group 1

- RCT
- Quasi-randomised study (e.g. alternation rather than randomisation used)
- Systematic reviews of RCTs or quasi-randomised studies

Group 2

- Cross-sectional study
- Case-series
- Systematic reviews of cross-sectional studies or case-series

If yes then retrieve full article for consideration for inclusion
