# Supplementary figures and images for: Surgical intervention for paediatric infusion-related extravasation injury: a systematic review
Source: BMJ Open. 2020 Aug 6;10(8):e034950. doi: 10.1136/bmjopen-2019-034950 (PMC7412604; doi:10.1136/bmjopen-2019-034950)

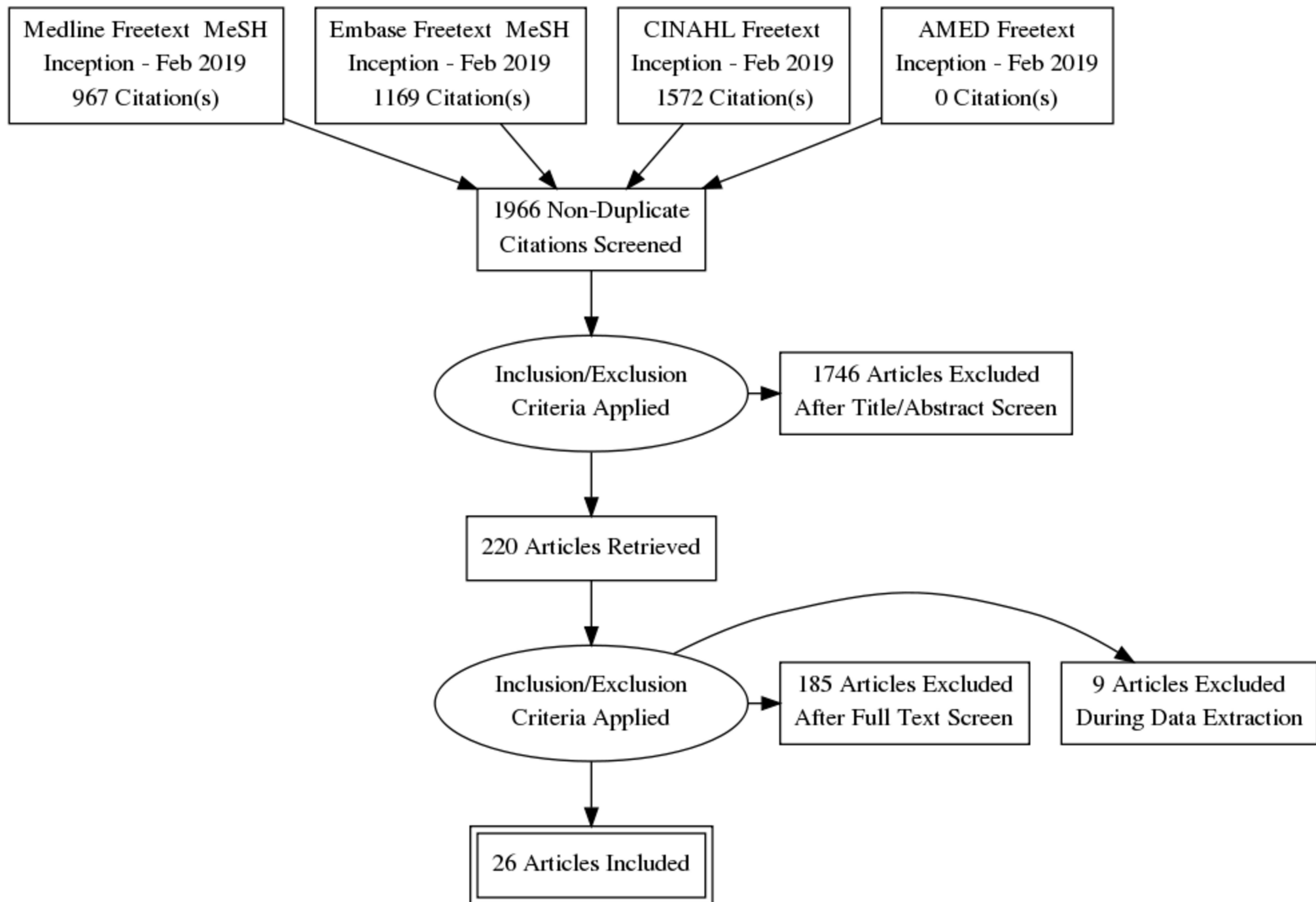

Supplement: Supplementary data [file bmjopen-2019-034950supp004.pdf]
