# Supplementary material for: Surgical intervention for paediatric infusion-related extravasation injury: a systematic review
Source: BMJ Open. 2020 Aug 6;10(8):e034950. doi: 10.1136/bmjopen-2019-034950 (PMC7412604; doi:10.1136/bmjopen-2019-034950)
Supplement: Supplementary data [file bmjopen-2019-034950supp003.pdf]

BMJ Publishing Group Limited (BMJ) disclaims all liability and responsibility arising from any reliance placed on this supplemental material which has been supplied by the author(s)

Supplementary 3
<u>Review-Specific Proforma</u>

Author	Patients (n)	Age (mean)	Gender		Comorbidities				Cannula location			Cannula type	
			M	F	Premature	Sepsis	Malignancy	Other	Upper limb	Lower limb	Scalp	Periph eral	Central

Author	Vesicants							Medical Management	Surgical Management	Control (Y/N)
	TPN	≥5% Dextrose	Other IV Fluids	Calcium-containing fluids	Antibiotics	Contrast	Other			

Author	Surgical injuries								Wound outcome	Functional outcome
	Swelling/ Erythema	Partial thickness skin injury	Full thickness skin injury	Tendon injury	Nerve injury	Vascular injury	Compartment Syndrome	Amputation		
