# Supplementary material for: Surgical intervention for paediatric infusion-related extravasation injury: a systematic review
Source: BMJ Open. 2020 Aug 6;10(8):e034950. doi: 10.1136/bmjopen-2019-034950 (PMC7412604; doi:10.1136/bmjopen-2019-034950)
Supplement: Author's manuscript [file bmjopen-2019-034950.draft_revisions.pdf]

BMJ Open

BMJ Open is committed to open peer review. As part of this commitment we make the peer review history of every article we publish publicly available.

When an article is published we post the peer reviewers' comments and the authors' responses online. We also post the versions of the paper that were used during peer review. These are the versions that the peer review comments apply to.

The versions of the paper that follow are the versions that were submitted during the peer review process. They are not the versions of record or the final published versions. They should not be cited or distributed as the published version of this manuscript.

BMJ Open is an open access journal and the full, final, typeset and author-corrected version of record of the manuscript is available on our site with no access controls, subscription charges or pay-per-view fees (http://bmjopen.bmj.com).

If you have any questions on BMJ Open's open peer review process please email info.bmjopen@bmj.com

BMJ Open

Surgical Intervention for Paediatric Infusion-Related Extravasation Injury: A Systematic Review

Journal:	*BMJ Open*
Manuscript ID	bmjopen-2019-034950
Article Type:	Original research
Date Submitted by the Author:	22-Oct-2019

Complete List of Authors:	Little, Max; Whittington Hospital NHS Trust, General Surgery<br>Dupré, Sophie; University of Oxford Nuffield Department of Orthopaedics Rheumatology and Musculoskeletal Sciences<br>Wormald, Justin; University of Oxford Nuffield Department of Orthopaedics Rheumatology and Musculoskeletal Sciences; Stoke Mandeville Hospital, Plastic & Reconstructive Surgery<br>Gardiner, Matthew; University of Oxford Nuffield Department of Orthopaedics Rheumatology and Musculoskeletal Sciences; Wexham Park Hospital, Plastic & Reconstructive Surgery<br>Gale, Chris; Imperial College London, Neonatal Medicine, School of Public Health<br>Jain, Abhilash; University of Oxford Nuffield Department of Orthopaedics Rheumatology and Musculoskeletal Sciences, Plastic Surgery; Imperial College Healthcare NHS Trust, Plastic & Reconstructive Surgery

Keywords:	Paediatric plastic & reconstructive surgery < PAEDIATRIC SURGERY, PLASTIC & RECONSTRUCTIVE SURGERY, Paediatric intensive & critical care < PAEDIATRICS, Paediatric oncology < ONCOLOGY

SCHOLARONE™
Manuscripts

I, the Submitting Author has the right to grant and does grant on behalf of all authors of the Work (as defined in the below author licence), an exclusive licence and/or a non-exclusive licence for contributions from authors who are: i) UK Crown employees; ii) where BMJ has agreed a CC-BY licence shall apply, and/or iii) in accordance with the terms applicable for US Federal Government officers or employees acting as part of their official duties; on a worldwide, perpetual, irrevocable, royalty-free basis to BMJ Publishing Group Ltd ("BMJ") its licensees and where the relevant Journal is co-owned by BMJ to the co-owners of the Journal, to publish the Work in this journal and any other BMJ products and to exploit all rights, as set out in our licence.

The Submitting Author accepts and understands that any supply made under these terms is made by BMJ to the Submitting Author unless you are acting as an employee on behalf of your employer or a postgraduate student of an affiliated institution which is paying any applicable article publishing charge ("APC") for Open Access articles. Where the Submitting Author wishes to make the Work available on an Open Access basis (and intends to pay the relevant APC), the terms of reuse of such Open Access shall be governed by a Creative Commons licence – details of these licences and which Creative Commons licence will apply to this Work are set out in our licence referred to above.

Other than as permitted in any relevant BMJ Author's Self Archiving Policies, I confirm this Work has not been accepted for publication elsewhere, is not being considered for publication elsewhere and does not duplicate material already published. I confirm all authors consent to publication of this Work and authorise the granting of this licence.

For peer review only

Surgical Intervention for Paediatric Infusion-Related Extravasation Injury: A Systematic Review

Little, Max[1]; Dupré, Sophie[2]; Wormald, Justin[3]; Gardiner, Matthew[4]; Gale, Chris[5]; Jain, Abhilash[2,6]

1. Department of Surgery, The Whittington Hospital, Magdala Avenue, London, UK, N19 5NF
2. Nuffield Department of Orthopaedics, Rheumatology and Musculoskeletal Sciences, University of Oxford, Oxford, UK, OX1 2DJ
3. Department of Plastic Surgery, Stoke Mandeville Hospital, Aylesbury, Buckinghamshire, UK, HP21 8AL
4. Department of Plastic Surgery, Wexham Park Hospital, Wexham St, Slough, UK, SL2 4HL
5. Neonatal Medicine, Department of Medicine, Imperial College London, Chelsea and Westminster Campus, London, UK, SW10 9NH
6. Department of Plastic and Reconstructive Surgery, Imperial College Healthcare NHS Trust, St Mary's Hospital, Praed St, London, UK, W2 1NY

Mr Max Little MBBS MRCS
Core Surgical Trainee, The Whittington Hospital NHS Trust, London, UK

Dr Sophie Dupré MBBS BSc (Hons)
Academic Foundation Doctor, University of Oxford, UK

Mr Justin C R Wormald MBBS MRes MRCS
NIHR Academic Clinical Fellow and Specialist Registrar in Plastic and Reconstructive Surgery, University of Oxford, UK

Mr Matthew Gardiner MA PhD FRCS (Plast)
Hand Fellow, NIHR Senior Clinical Lecturer and Specialist Registrar in Plastic and Reconstructive Surgery, University of Oxford, UK

Dr Chris Gale MBBS MSc PhD MRCPCH *
Clinical Senior Lecturer in Neonatal Medicine and Consultant Neonatologist, Imperial College London, UK

Professor Abhilash Jain MBBS MRCS MSc PhD FRCS (Plast) *
Associate Professor of Plastic and Reconstructive Surgery, University of Oxford and Imperial College NHS Trust, London, UK
*Co-Senior Authors

Corresponding author:
Dr Chris Gale
christopher.gale@imperial.ac.uk
+44 (0)20 3315 3519
Academic Neonatal Medicine, H4.4
Chelsea and Westminster Hospital
Chelsea and Westminster Campus

Running Head: Paediatric extravasation injury

Keywords: Extravasation; Infiltration; Paediatric; Necrosis; Gault

Word Count: 3,980 (including abstract and summary)

For peer review only

Abstract

Objectives

This systematic review aims to assess the quality of literature supporting surgical interventions for paediatric extravasation injury and to determine and summarize their outcomes.

Methods

We performed a systematic review by searching Ovid MEDLINE and EMBASE as well as AMED, CINAHL, Cochrane Central Register of Controlled Trials (CENTRAL), Cochrane Database of Systematic Reviews and clinicaltrials.gov from inception to February 2019. All studies other than case reports were eligible for inclusion if the population was younger than 18 years old, there was a surgical intervention aimed at treating extravasation injury and they reported on outcomes. Risk of bias was graded according to the National Institutes of Health (NIH) study quality assessment tools.

Results

26 studies involving 728 children were included – one before-and-after study and 25 case series. Extravasation injuries were mainly confined to skin and subcutaneous tissues but severe complications were also encountered, including amputation (one toe and one below elbow). Of the surgical treatments described, the technique of multiple puncture wounds and instillation of saline and/or hyaluronidase was the most commonly used. However, there were no studies in which its effectiveness was tested against another treatment or a control and details of functional and aesthetic outcomes were generally lacking.

Conclusion

There is a lack of high quality evidence to support treatment of extravasation injury in children. A definitive trial of extravasation injuries, or a centralized extravasation register using a universal grading scheme and core outcome set with adequate follow-up, are required to provide evidence to guide clinician decision-making.

For peer review only

For peer review only - http://bmjopen.bmj.com/site/about/guidelines.xhtml

Strengths and Limitations

- A systematic review was performed according to PRISMA guidelines and registered on PROSPERO
- Two authors used a bespoke inclusion/exclusion form to independently assess study eligibility
- Studies were eligible for inclusion if the population was younger than 18 years old, if there was a surgical intervention aimed at treating extravasation injury in any setting and if they reported on short- or long-term outcomes
- Two researchers also independently assessed the included studies' risk of methodological bias using the National Institutes of Health (NIH) study quality assessment tools
- 18 years old may represent a relatively arbitrary cut-off age to differentiate between 'paediatric' and 'adult' in terms of extravasation injury

For peer review only

Introduction

Extravasation is the inadvertent leakage of a vesicant solution from its intended vascular pathway, commonly a peripheral or central vein, into the surrounding tissue with the potential to harm a patient. A vesicant is any medicine or fluid with the potential to cause blisters, severe tissue injury or necrosis if there is leakage. [1] If left untreated, extravasations may result in delayed healing, scarring and functional morbidity, including amputation in severe cases. Children requiring intravenous (IV) therapy often have multiple risk factors for extravasation injuries, and neonates are at even greater risk of more serious injury due to poor venous integrity, capillary leakage, and expandable subcutaneous tissue that accommodates relatively large volumes of fluid. [2]

The reported incidence of extravasation in adult and paediatric inpatients ranges between 0.1% - 6.5% across studies from the UK, USA and Canada, but inconsistent reporting and documentation means that the true figure is likely to be higher. [2–4]

Multiple treatments have been described. The “Gault technique” [5] utilizes multiple small incisions made around the injury site. These are subsequently used to instill saline and/or hyaluronidase to wash out the extravasation fluid. This method remains a commonly described technique for the treatment of more severe paediatric extravasations. However, there is a lack of high quality comparative studies in which treatments are evaluated against each other and/or controls. It is therefore not known which of these treatments are effective, harmful or at the very least, necessary; a conclusion supported by a recent scoping review of interventions. [6] That review highlighted the fact that the lack of consensus extended to published guidelines. Their survey also showed wide variation in the frequency of use of the saline washout techniques in neonatal units across the National Health Service (NHS) in the UK. This systematic review aims to evaluate the quality of the literature and to summarize the published outcome data of the surgical management of paediatric extravasation injuries.

For peer review only - http://bmjopen.bmj.com/site/about/guidelines.xhtml

Methods

We performed a systematic review of original clinical studies or systematic reviews (excluding case reports) of paediatric extravasation injury, reported in accordance with the Preferred Reporting Items for Systematic Reviews and Meta-Analyses (PRISMA) [7] statement and using methodology outlined in the Cochrane Handbook for Systematic Reviews of Interventions [8] where applicable. The protocol was developed prospectively, locally peer-reviewed and registered on the PROSPERO database (CRD42016045647).

Search methods

Studies were identified through a systematic literature search in Ovid MEDLINE and Ovid EMBASE as well as AMED, CINAHL, Cochrane Central Register of Controlled Trials (CENTRAL), Cochrane Database of Systematic Reviews and clinicaltrials.gov from inception to February 2019. To identify grey literature, Web of Science was searched using bespoke searches. Internet-wide searches using Google were employed to identify clinical guidelines describing the management of extravasation in the UK. All of these strategies comprised modifications of a common set of terms to identify the relevant clinical area. Key search terms in this common set included the Medical Subject Headings (MeSH) “extravasation of diagnostic and therapeutic materials", “infant”, “child”, and “preschool”. These MeSH terms were combined with appropriate Boolean operators [Supplementary 1]. The index search terms were then combined with free text terms to identify appropriate clinical studies. No language restrictions were imposed.

The search results were merged and, after screening, duplicate citations were discarded along with studies unrelated to the research objective.

Data availability

All relevant data are included in the article or uploaded as supplementary information.

For peer review only

Criteria for selecting studies

Study selection criteria were determined in advance during the protocol stage. Two authors used a bespoke inclusion/exclusion form to independently assess eligibility of the title and abstracts of the search results. [Supplementary 2] Randomized Control Trials (RCTs), quasi-experimental and cross-sectional studies and case-series were eligible for inclusion if the population was younger than 18 years old, if there was a surgical intervention aimed at treating extravasation injury in any setting and if they reported on short- or long-term outcomes. Any disparities regarding inclusion of articles were discussed between the authors and a joint decision was made based on the inclusion criteria.

Assessment of Bias

Two researchers also independently assessed the included studies' risk of methodological bias using the National Institutes of Health (NIH) study quality assessment tools [9] for "case series" and for "before-after studies with no control group" – the two types of included studies. Discrepancies were resolved by discussions with the other authors.

Patient and Public Involvement

No patients involved

For peer review only

Results

Search strategy

Our search strategy identified a total of 1,966 research articles of which 220 were potentially relevant to the research question. Of the 220 papers 185 did not meet the inclusion criteria. Thirty-five papers were deemed eligible for inclusion after studies in which conservative management or no intervention were excluded. The requisite data were immediately available in 26 study reports [4,10,19–28,11,29–34,12–18] and the authors of the remaining nine studies [5,35–42] were contacted twice with additional requests, mainly for exclusively paediatric extravasation data, and given at least two months to reply. Unfortunately, none of the authors were able to provide these data and those studies were excluded. The data were extracted from the remaining 26 studies [Supplementary PRISMA flow-diagram] using a pre-specified (review-specific) proforma [Supplementary 3] by two researchers, with discrepancies resolved by a third.

Study Characteristics

The included studies were published between 1975 and 2018. The mean sample size was 32 with a range of 4-147 participants. One study was a before-and-after comparative study, [31] two were prospective case series, [19,24] and the remaining 23 were retrospective case series. The follow-up periods were not reported in 14 included studies. In the other studies, the follow-up periods ranged from three days to 15 months [Table 1].

For peer review only - http://bmjopen.bmj.com/site/about/guidelines.xhtml

Table 1 – Included Studies

Rf	Author	Year	Country	Title	n	Methods	Period	Mean follow-up
4	Murphy	2017	Australia	Extravasation injury in a paediatric population	43	Retrospective Series	-	-
10	Falcone	1989	USA	Nonoperative management of full-thickness intravenous extravasation injuries in premature neonates using enzymatic debridement	15	Retrospective Series	-	6 months
19	Ghanem	2015	UK	Childhood extravasation injuries: improved outcome following the introduction of hospital-wide guidelines	48	Prospective Series	1 year	-
20	Kostogloudis	2015	Greece	Severe Extravasation Injuries in Neonates: A Report of 34 Cases	34	Retrospective Series	2 years	15 months
21	Sung	2016	Korea	Nonoperative Management of Extravasation Injuries Associated With Neonatal Parenteral Nutrition Using Multiple Punctures and a Hydrocolloid Dressing.	12	Retrospective Series	4 years	10 months
22	Odom	2018	USA	Peripheral Infiltration and Extravasation Injury Methodology: A Retrospective Study.	147	Retrospective Series	-	-
23	Compaña	2017	Spain	Lesions Associated With Calcium Gluconate Extravasation	4	Retrospective Series	-	2 months
24	Harris	2001	UK	Limiting the damage of iatrogenic extravasation injury in neonates	56	Prospective Series	3 years	-
25	Andrés	2006	Spain	Treatment protocol for extravasation lesions	15	Retrospective Series	6 years	-
26<br>27	Linder	1983<br>1985	USA	Management of extensive doxorubicin hydrochloride extravasation injuries<br>Prevention of extravasation injuries secondary to doxorubicin	18	Retrospective Series	-	3 months
28	Upton	1979	USA	Major intravenous extravasation injuries	7	Retrospective Series	10 years	-
11	Casanova	2001	France	Emergency treatment of accidental infusion leakage in the newborn: Report of 14 cases	14	Retrospective Series	-	-
29	von Heimburg	1998	Germany	Early and late treatment of iatrogenic injection damage	5	Retrospective Series	-	-
30	Weiss	1975	Israel	Localized necrosis of scalp in neonates due to calcium gluconate infusions: a cautionary note	4	Retrospective Series	-	-
31	Hanrahan	2013	USA	Hyaluronidase for treatment of intravenous extravasations: implementation of an evidence-based guideline in a pediatric population	113	Before-and-after study	4 years	-
32	Boyar	2018	USA	Efficacy of Dehydrated Human Amniotic Membrane Allograft for the Treatment of Severe Extravasation Injuries in Preterm Neonates	4	Retrospective Series	-	1-2 months
33	Yan	2017	China	Incidence, risk factors and treatment outcomes of drug extravasation in pediatric patients in China	18	Retrospective Series	6 months	1– 5 months
34	Myers	2018	USA	Managing intravenous infiltration injuries in the neonatal intensive care unit	28	Retrospective Series	7 years	-
12	Cochran	2002	USA	Treatment of iodinated contrast material extravasation with hyaluronidase	8	Retrospective Series	7 years	-
13	Cho	2007	Korea	Successful combined treatment with total parenteral nutrition fluid extravasation injuries in preterm infants	5	Retrospective Series	4 months	-
14	Onesti	2012	Italy	The use of hyalomatrix PA in the treatment of extravasation affecting premature neonates	26	Retrospective Series	6 years	14 months
15	Firat	2013	Turkey	Management of extravasation injuries: a retrospective study	13	Retrospective Series	2 years	-
16	Ching	2014	UK	Paediatric extravasation injuries: a review of 69 consecutive patients	69	Retrospective Series	1 year	3 days
17	Sivrioglu	2014	Turkey	Versajet hydrosurgery system in the debridement of skin necrosis after Ca gluconate extravasation: report of 9 infantile cases	9	Cohort Study	-	1 year
18	Yan	2014	China	Treatment of cutaneous injuries of neonates induced by drug extravasation with hyaluronidase and hirudoid	13	Retrospective Series	2 years	3 months

For peer review only

Patient Characteristics

Across the 26 included studies, there were 728 children. The median age of the 68 children for whom the individual data was available was 0.66 months (interquartile range (IQR) = 5.05). The mean age of 14 months (range: neonatal to 17 years old) was calculated using the data for 322 children across 16 included studies. There was a small difference in sex, with 187 males (57%) and 142 females (43%) (data on sex was lacking for 399 children). 19 of the 26 included studies recorded the sites of the implicated cannula for 553 children. The most common site was the upper limb (n=343; 62%), followed by the lower limb (n=177; 32%) and then the scalp (n=21; 4%). Ten studies specifically stated whether the cannula was a peripheral (n=314; 98%) or central line (n=7; 2%). [Table 2]

For peer review only

Table 2 – Demographics

Ref	Author	n	Mean age (months)	Male	Female	N upper limb	N lower limb	N Scalp	N other	Peripheral Cannula	Central Cannula
**Gault Technique Studies**											
4	Murphy	43	-	-	-	32	9	-	-	-	-
11	Ghanem	48	38.4	-	-	48	25	4	5	73	7
12	Kostogloudis	34	0.6	-	-	6	28	-	-	34	-
17	Andrés	15	36	-	-	14	-	1	-	15	-
21	Casanova	14	1	-	-	4	9	1	-	-	-
32	Ching	69	0.7	32	37	45	17	-	7	-	-
24	Harris	56	-	-	-	-	-	-	-	-	-
	**Total**	**279**	**15.3 (mean)**	**32**	**37**	**149**	**88**	**6**	**12**	**122**	**7**
**Debridement + further surgery**											
10	Falcone	15	0.6	7	8	12	2	1	-	-	-
13	Sung	12	-	6	6	7	5	-	-	-	-
15	Compaña	4	2.6	3	1	2	2	-	-	4	-
17	Andrés (2)	15	36	-	-	14	-	1	-	15	-
20	Upton	7	67.2	-	-	-	-	-	-	-	-
23	Weiss	4	-	-	-	-	-	4	-	4	-
25	Boyar	4	1.1	3	1	3	1	-	-	4	-
29	Cho	5	0.6	-	-	4	-	-	-	6	-
30	Onesti	26	0.6	17	9	14	10	2	-	-	-
31	Firat	13	50	3	10	6	4	3	-	-	-
33	Sivrioglu	9	0.9	-	-	5	3	1	-	9	-
26 27	Linder	18	-	-	-	-	-	-	-	-	-
29	Von Heimburg	5	-	-	-	-	-	-	-	-	-
	**Total**	**137**	**17.7 (mean)**	**46**	**28**	**67**	**27**	**12**	**0**	**42**	**0**
**Hyaluronidase injections**											
14	Odom	147	-	87	60	106	40	2	-	147	-
26	Yan (2017)	18	39.7	10	8	12	6	-	-	18	-
27	Myers	28	1.3	-	-	14	13	1	-	-	-
28	Cochran	8	-	4	4	-	-	-	-	-	-
34	Yan (2014)	13	0.9	8	5	9	3	1	-	-	-
31	Hanrahan	113	-	-	-	-	-	-	-	-	-
	**Total**	**327**	**14.0 (mean)**	**109**	**77**	**141**	**62**	**4**	**0**	**165**	**0**
	**Overall Totals**	**728**	**15.13 (mean)**	**187**	**142**	**343**	**177**	**21**	**12**	**314**	**7**

For peer review only

For peer review only - http://bmjopen.bmj.com/site/about/guidelines.xhtml

Of the 16 studies that provided data on comorbidities for 167 children, prematurity was the most common comorbidity in 11 (n=121; 72%). Malignancy (n=25; 15%) was the main comorbidity in three studies.

Of the 20 studies that reported on them, the most commonly extravasated substances affecting 363 children were [Table 3]:

1) Total Parenteral Nutrition (TPN) – 122 children (34%); most common in eight studies.
2) Intravenous maintenance fluids other than those specifically listed – 70 children (19%); most common in three studies. Unfortunately the data was not available for the outcomes of different types of maintenance fluids so they have been grouped together.
3) Calcium-containing products – 32 children (9%); most common in four studies.
4) Dextrose of concentration ≥5% - 28 children (8%); most common in one study.
5) Intravenous antimicrobials – 20 children (6%); most common in no studies. Of those 20 extravasations, five were due to Flucloxacillin with one injury taking 60 days to heal and five were due to unspecified cephalosporin antibiotics, three of which required skin grafting. One case of severe contractures and extensor loss on the dorsum of a child’s hand was due to an unspecified tetracycline antibiotic. There were no individual outcomes recorded for the seven extravasations of Aciclovir, the single extravasations of Gentamicin, Ganciclovir or the remaining extravasations involving Flucloxacillin and cephalosporin antibiotics.

For peer review only

Table 3 – Comorbidities and extravasated materials in those papers that recorded them

		Comorbidities			Vesicant Type								
Ref	Author	Prematurity	Sepsis	Malignancy	TPN	≥5% Dextrose	Other IV Fluids	Calcium-containing	Antimicrobials	Chemo	Contrast	Dopamine	Other
**Gault Technique Studies**													
4	Murphy	-	-	-	10	11	6	2	6	-	2	-	5
19	Ghanem	14	-	-	22	10	6	-	8	-	-	1	1
20	Kostogloudis	34	-	-	28	4	-	-	2	-	-	-	-
25	Andrés	-	1	5	7	-	-	4	-	4	-	-	-
11	Casanova	-	-	-	-	-	-	2	-	-	-	9	3
16	Ching	"Majority"	-	-	16	-	22	-	-	-	-	-	31
	**Total**	**48**	**1**	**5**	**83**	**25**	**34**	**8**	**16**	**4**	**2**	**10**	**40**
**Debridement + further surgery**													
10	Falcone	15	-	-	9	1	3	1	-	-	-	-	2
21	Sung	9	-	-	12	-	-	-	-	-	-	-	-
23	Compaña	2	-	-	-	-	-	4	-	-	-	-	-
25	Andrés (2)	-	1	5	7	-	-	4	-	4	-	-	-
26 27	Linder	-	-	18	-	-	-	-	-	18	-	-	-
28	Upton	-	-	1	-	1	2	1	1	2	-	-	-
30	Weiss	4	-	-	-	-	-	4	-	-	-	-	-
32	Boyar	4	-	-	4	-	-	-	-	-	-	-	-
13	Cho	5	-	-	4	-	-	-	-	-	-	-	1
14	Onesti	26	-	-	-	-	26	-	-	-	-	-	-
15	Firat	-	1	1	-	1	4	3	3	1	-	-	1
17	Sivrioglu	3	-	-	-	-	-	9	-	-	-	-	-
	**Total**	**68**	**2**	**25**	**36**	**3**	**35**	**26**	**4**	**25**	**0**	**0**	**4**
**Hyaluronidase injections**													
33	Yan (2017)	-	-	-	1	-	-	1	-	-	-	-	-
12	Cochran	-	-	-	-	-	-	-	-	-	8	-	-
18	Yan (2014)	5	-	-	9	-	1	1	-	-	-	-	2
	**Total**	**5**	**0**	**0**	**10**	**0**	**1**	**2**	**0**	**0**	**8**	**0**	**2**
	**Overall Totals**	**121**	**2**	**25**	**122**	**28**	**70**	**32**	**20**	**25**	**10**	**10**	**46**

For peer review only

For peer review only - http://bmjopen.bmj.com/site/about/guidelines.xhtml

Interventions

Uncertainty remains over whether there is a difference in the outcomes of children who are treated conservatively or surgically and the case series that exist cover a number of treatments for a range of injuries from swelling to full-thickness defects. However, a recent scoping review found that the outcomes used and results detailed were generally limited, [6] making it difficult for clinicians to rely upon those studies in deciding who to treat conservatively. This is why we have focused solely on surgical interventions.

The Gault Method

279 children with an average age of 15.3 months were involved in the seven studies looking at the Gault method. No patients had skin necrosis at presentation.

107 were treated using the Gault method with saline, of which nine (8%) developed full thickness skin injury / skin necrosis and one (1%) developed compartment syndrome requiring fasciotomies. Of those with necrosis, six required no surgical intervention and three were debrided and treated with artificial dermis.

76 were treated using the Gault method with hyaluronidase, of which three (4%) developed skin necrosis and were all managed conservatively.

One child received only hyaluronidase injections and developed necrosis, which healed spontaneously.

95 children received no treatment, of which ten (11%) developed necrosis (one of whom went on to lose three toes), three (3%) developed an associated infection, one (1%) had an ischaemic toe requiring amputation and one (1%) developed calcinosis cutis. [Table 4]

For peer review only

Table 4 – Interventions

Ref	Author	Intervention Summary	Outcomes
**Gault Technique Studies**			
4	Murphy	Gault's + saline (11% of patients).	Three patients (7%) suffered injuries, which led to significant tissue necrosis, delayed healing and prolonged morbidity. None of these were washed out due to delayed referral.
20	Kostogloudis	Gault's + saline (100% of patients).	All wounds healed within 25 days. One case of distal foot ischaemia resolved after treatment.
24	Harris	Gault's + saline (100% of patients).	No episodes of skin or soft tissue loss were recorded and no reconstructive surgery was required.
25	Andrés	Gault's + saline (67% of patients) + Debridement + dermal substitute in 53%	Seven of ten treated with Gault's method avoided necrosis and recovered fully. Three developed necrosis and, with the remaining five, were debrided and received artificial skin and obtained satisfactory outcomes.
11	Casanova	Gault's + hyaluronidase (79% of patients) / + saline (14%)	No skin involvement in ten patients; Blistering healed in one; Necrosis resolved in three
19	Ghanem	Gault's + hyaluronidase (46% of patients).	Three patients had tissue necrosis (one of whom received wash out). There was satisfactory healing with no requirement for surgical intervention.
16	Ching	Gault's + hyaluronidase (62% of patients).	Of the 62% of patients washed out, none developed complications. Two other significant complications.
**Debridement + further surgery**			
10	Falcone	Topical fibrinolysin/deoxyribonuclease ointment then debridement.	All wounds healed completely with no infections and no functional scar contractions at up to 16 months follow-up. No skin grafts were required.
21	Sung	Multiple punctures using a scalpel blade + hydrocolloid dressing. Then debridement.	All patients showed favourable results without functional deficits or conspicuous scars
23	Compaña	Topical steroids, Burow's Solution and Silver Sulfadiazine. 3 debridements followed by split-thickness skin grafts.	Of four patients, two successfully healed, one was lost to follow-up and one died of other causes.
25	Andrés (2)	Gault's + saline (67% of patients) + debridement in 33% + artificial dermis.	Seven of the ten patients treated with Gault's method avoided necrosis and recovered fully. Three developed necrosis. The five who were debrided and received artificial skin obtained satisfactory outcomes.
30	Weiss	Wet dressings and repeated economical debridement.	Wounds healed well in 15 to 40 days. Scars were visible but without discolouration
32	Boyar	Enzymatic or autolytic debridement before mechanical debridement and application of dehydrated human amniotic membrane allograft (dHAMA)	Complete closure of significant wounds with minimal soft scars and normal pigmentation
13	Cho	Topical antibiotic + anti-inflammatory herbal mixture. 1 debridement. 1 escharotomy.	One patient had pigmentation at four weeks, one had a small-sized contracture at 50 days, one had small linear scar at two years.
14	Onesti	Topical collagenase, then debridement, and then Hyalomatrix PA (dermal substitute).	18 patients healed fully after 21 days. Four had pathological scars and four had debilitating scar contractures needing secondary surgery.
15	Firat	Topical hirudin and antibiotics, then 3% boric acid, then repetitive debridement.	Seven patients required split-thickness skin grafting and two required fasciocutaneous flaps. All recovered well with scar development in four. Minor functional loss in the hands or feet as a result of scar formation was managed by physiotherapy and pressure garments.
26<br>27	Linder	Debridement and wound closure: mostly split-thickness skin grafts or delayed primary closure. All patients had at least 2 operations.	The mean time for wound closure was 49 days (range 10–85 days). Three patients died before wound closure. At least one patient needed a split-thickness skin graft. One child developed sympathetic dystrophy syndrome. Some children developed permanent joint stiffness.
28	Upton	Debridement and skin grafts. All patients needed two or more operations.	Two patients experienced contractures, two had extensor loss, one had hair loss, one had loss of motion and one required further reconstruction.
29	von Heimburg	Debridement, allogeneic donor tissue grafts and autologous split-thickness skin grafts.	After 15 days there was full healing in all five infants
17	Sivrioğlu	Versajet hydrosurgery for all patients. 1 sharp debridement.	Minimal scar formation with no hypertrophic scarring in any patient.
**Hyaluronidase injections**			
22	Odom	Injection of hyaluronidase or Phentolamine without incisions.	No patients required surgical intervention for wound healing or had an infection.
31	Hanrahan	Injection of hyaluronidase.	Mean harm scores were similar between the group receiving hyaluronidase and the group not receiving it.
33	Yan (2017)	Injection of hyaluronidase in 33% (1 patient requiring surgical excision of a lesion)	All healed and had "good outcomes"
34	Myers	Injection of hyaluronidase in 50%	Time to healing averaged 16.2 days (range 1-82 days). No patient required surgical intervention.
12	Cochran	Injection of hyaluronidase in 25%	One patient had a prolonged course with swelling and skin peeling of the hand.
18	Yan (2014)	Injection of hyaluronidase and application of hirudoid.	Three patients lost to follow-up. Negligible loss of functional movements. One case of scarring and readmission with calcinosis.

For peer review only - http://bmjopen.bmj.com/site/about/guidelines.xhtml

Debridement and further surgery

There were 137 children with an average age of 17.7 months across the 14 studies in the debridement and further surgery group.

Nine studies (98 children) used management plans comprising debridement and further surgery in combination with conservative measures, details of which are shown in Table 4. [10,13–15,21,23,25,30,32] Of those 98 children, 34 (35%) had established full thickness skin injury / skin necrosis at presentation and 28 (29%) developed necrosis after conservative measures. 84 children (86%) underwent mechanical debridement as part of their treatment. The vast majority of children's wounds healed with minimal scarring and no functional deficit but four (4%) had debilitating scar contractures necessitating further surgery, ten (10%) required skin grafts, and two (2%) required fasciocutaneous flaps.

The other five studies (39 children) used only debridement and further surgery. All of those 39 children had full thickness skin injury / skin necrosis at presentation. 30 (77%) underwent mechanical debridement, of whom 22 (56%) required further operations including skin grafts and fasciocutaneous flaps and two developed contractures. The remaining nine children (23%), all from one study, underwent Versajet hydrosurgery with good healing in all children and one requiring sharp debridement.

In total, of the 137 children in the debridement and further surgery group, 103 (75%) developed skin necrosis, all of whom (in addition to some of those who were yet to develop necrosis, total n=123 (89%)) received either mechanical or water jet debridement. At least 38 children (28%) went on to require further operations.

Hyaluronidase injections

The final group of studies used hyaluronidase injections at the extravasation site without the need for incisions or washouts. This group comprised six studies and 327 children. [12,18,22,31,33,34]

97 children received hyaluronidase injections, one (1%) of whom had skin necrosis at presentation that resolved, and one (1%) developed necrosis requiring further surgery. Of the remaining 230 children that did not receive hyaluronidase, none developed skin necrosis at any stage, although reporting on the extent of skin damage and eventual outcomes across the studies was generally poor.

For peer review only

Comparison of the interventions

The Gault Method

Among the studies included in this review, Ghanem et al [19] provides the closest approximation of a comparative study of the Gault method in children: 48 extravasations were diagnosed and children were divided into early (<24 hours, n=45) and later (>24 hours, n=3) referrals. Among early referrals, 22 were deemed to be 'at risk' injuries and received washout using Gault's method. Skin necrosis occurred in one of 45 early referrals but in two of three later referrals. The conclusion of this study, that the incidence of necrosis is higher in the later referral group, is limited by low methodological quality including the use of an arbitrary 24-hour cut-off, the small size of the later referral group and a lack of data describing the types of vesicants. It is important to note that the necrosis resolved with conservative management in all three cases.

Of the 183 children treated with a variation of the Gault technique across the included studies, 12 (7%) developed skin necrosis (none had skin necrosis at presentation). This compares favourably to the 11% (10/95) of children who received no treatment across those same studies. However, 73% (69/95) of the untreated group were late referrals and may be expected to have worse outcomes related to time of presentation. The only study to apply the Gault technique in which plastic surgery review time and patient ages were similar was Ching, Wong and Milroy. [16] In that study, none of the 43 children treated with Gault's technique and hyaluronidase developed any complications whereas there were three cases of associated infection, one ischaemic toe requiring amputation and one case of calcinosis cutis amongst the 26 children that received no treatment.

The degree of injury was only recorded for 36 (13%) of the 279 children in the included studies describing the Gault technique, of which 22 (8%) were full-thickness skin injuries.

Debridement and further surgery

73 children (53%) of the 137 in the debridement and further surgery studies had at least skin necrosis / full thickness skin injury at presentation

For peer review only - http://bmjopen.bmj.com/site/about/guidelines.xhtml

In contrast to the Gault as well as the hyaluronidase injection studies, the degree of injury was documented for 93% of the children (128/137).

Hyaluronidase injections

One (0.3%) of the 327 children reported in studies describing hyaluronidase injections had skin necrosis at presentation, and one (0.3%) went on to develop it. The degree of injury was recorded for only 49 (15%) of the 327 children. The reporting of patient outcomes was particularly scarce in this group.

Injuries

The types of injury were recorded for 212 of the 728 children across the included studies. Injuries were limited to swelling, pain or erythema (n=63; 30%), partial-thickness skin injury such as superficial blistering in others (n=29; 14%) and full-thickness skin injury such as necrosis in the largest number of children (n=110; 52%). However, these figures are skewed by the fact that 10 studies only looked at extravasation injuries causing at least skin necrosis. [10,15,17,20,26–30,32] One study described a transient episode of foot ischaemia which resolved with conservative management and one compartment syndrome of the left forearm requiring fasciotomies. [20] Two studies described toe amputations secondary to ischaemia. [4,16] One study of major injuries with full-thickness tissue loss had several casualties including a below elbow amputation and two episodes of extensor loss. [28] Table 5 demonstrates the huge range of injury severity encountered and the number of each in the different intervention groups.

For peer review only

Table 5 – Injuries in those papers that recorded them

		Skin changes			Other injuries				
**Ref**	**Author**	**Pain/swelling/erythema**	**Partial thickness skin injury**	**Full thickness skin injury**	**Tendon**	**Nerve**	**Vascular**	**Compartment Syndrome**	**Amputation**
**Gault Technique Studies**									
4	Murphy	-	-	3	-	-	-	-	1
11	Casanova	10	1	3	-	-	-	-	-
16	Ching	-	-	2	-	-	-	-	1
19	Ghanem	-	-	3	-	-	-	-	
20	Kostogloudis	-	-	6	-	-	1	1	-
25	Andrés	-	-	8	-	-	-	-	-
	**Total**	**10**	**1**	**25**	**0**	**0**	**1**	**1**	**2**
**Debridement + further surgery**									
10	Falcone	-	-	16	-	-	-	-	-
13	Cho	1	2	3	-	-	-	-	-
14	Onesti	-	18	8	-	-	-	-	-
15	Firat	-	-	9	-	-	-	-	-
17	Sivrioglu	-	-	9	-	-	-	-	-
21	Sung	1	2	9	-	-	-	-	-
23	Compaña	-	1	3	-	-	-	-	-
25	Andrés (2)	-	-	8	-	-	-	-	-
26 27	Linder	-	-	18	-	-	-	-	-
28	Upton	-	-	7	2	-	-	-	1
29	von Heimburg	-	-	5	-	-	-	-	-
30	Weiss	-	-	4	-	-	-	-	-
32	Boyar	-	-	4	-	-	-	-	
	**Total**	**2**	**23**	**103**	**2**	**0**	**0**	**0**	**1**
**Hyaluronidase injections**									
12	Cochran	21	1	-	-	-	-	-	-
18	Yan (2014)	5	3	1	-	-	-	-	-
33	Yan (2017)	17	-	1	-	-	-	-	-
	**Total**	**43**	**4**	**2**	**0**	**0**	**0**	**0**	**0**
	**Overall Totals**	**55**	**28**	**122**	**2**	**0**	**1**	**1**	**3**

For peer review only - http://bmjopen.bmj.com/site/about/guidelines.xhtml

Millam's grading system for extravasation injuries

Several of the included studies [11,13,17,20,22,33] make reference to the four stages of extravasation/infiltration severity, which was first published by Millam in 1988. [43] This focuses on presence of pain, erythema, oedema, capillary refill, skin temperature and breakdown and it has been proposed that stages III & IV may require intervention. [44]

In one case series, [33] treatment was determined by the severity of extravasation injury based on a grading system similar to Millam's. Of the 18 children, the seven with Grade I and II injuries were managed conservatively. Ten children had grade III injuries – five were managed conservatively but the other five who had also developed skin erythema received active treatment with hyaluronidase injections. The one child with a grade IV injury required excision of the lesion – the sole surgical intervention in that study – after having been injected with hyaluronidase. All wounds healed with 'good outcomes'.

Risk of Bias

The one before-and-after study [31] was evaluated using a separate tool to the other studies (which were all case series). It was given an overall 'fair' rating by both authors. The vast majority of the case series were rated by both authors as 'good' or 'fair'.

All four of the studies rated as 'poor' lacked a clearly defined study population. Compaña, Míguez & de Toro Santos [23], Harris, Bradley & Moss [24], and von Heimburg & Pallua [29] did not clearly state their objective whilst Cochran, Bomyea & Kahn [12], Harris, Bradley & Moss and von Heimburg & Pallua lacked clearly-defined, valid and reliable outcome measures. Compaña, Míguez & de Toro Santos did not clearly describe the study's intervention and von Heimburg & Pallua's subject were not comparable. In combination, these factors led to the overall 'poor' rating of these four studies, meaning that their results were at high risk of methodological bias. [Table 6]

For peer review only - http://bmjopen.bmj.com/site/about/guidelines.xhtml

Table 6 – Risk of Bias for Included Studies

Case series

		Questions										
Ref	Study	1	2	3	4	5	6	7	8	9	Overall rating	Level of evidence
4	Murphy	Y	Y	N	Y	Y	Y	Y	–	Y	Good	4
10	Falcone	Y	Y	N	Y	Y	Y	Y	–	N	Good	4
11	Casanova	Y	Y	N	Y	Y	N	N	–	Y	Fair	4
12	Cochran	Y	N	N	Y	Y	N	N	–	Y	Poor	4
13	Cho	Y	Y	N	Y	Y	Y	Y	–	Y	Good	4
14	Onesti	N	Y	N	Y	Y	Y	Y	–	N	Fair	4
15	Firat	Y	Y	N	Y	Y	Y	Y	–	Y	Good	4
16	Ching	Y	Y	Y	Y	Y	Y	N	–	Y	Good	4
17	Sivrioglu	Y	Y	N	Y	Y	Y	Y	–	Y	Good	4
18	Yan 2014	Y	Y	N	Y	Y	Y	N	–	Y	Good	4
19	Ghanem	Y	Y	N	Y	N	N	Y	–	Y	Fair	4
20	Kostogloudis	Y	Y	N	Y	Y	Y	Y	–	Y	Good	4
21	Sung	Y	Y	N	Y	Y	N	Y	–	N	Good	4
22	Odom	Y	Y	N	Y	N	Y	N	–	Y	Good	4
23	Compaña	N	N	N	Y	N	Y	–	–	Y	Poor	4
24	Harris	N	N	Y	–	Y	N	–	–	Y	Poor	4
25	Andrés	Y	N	Y	N	Y	Y	–	–	Y	Good	4
26	Linder 1983	N	Y	Y	Y	Y	Y	–	–	Y	Good	4
27	Linder 1985	N	Y	Y	Y	Y	Y	Y	–	Y	Good	4
28	Upton	Y	N	Y	Y	Y	N	Y	–	Y	Good	4
29	von Heimburg	N	N	Y	N	Y	N	Y	–	Y	Poor	4
30	Weiss	N	Y	Y	Y	Y	N	Y	–	Y	Fair	4
32	Boyar	Y	Y	N	Y	Y	Y	Y	–	Y	Good	4
33	Yan 2017	Y	N	Y	–	Y	N	Y	Y	Y	Good	4
34	Myers	Y	Y	Y	Y	Y	Y	Y	–	Y	Good	4
5	Gault	N	N	Y	N	Y	Y	N	–	Y	Poor	4

Key	Y	N	–
	Yes	No	N/A, Not Recorded, or Cannot Determine

Before-and-after study

		1	2	3	4	5	6	7	8	9	10	11	12	Overall rating	Level of evidence
31	Hanrahan	Y	N	–	–	Y	Y	Y	N	–	Y	N	N	Fair	3

For peer review only

For peer review only - http://bmjopen.bmj.com/site/about/guidelines.xhtml

Discussion

This paper describes a systematic review of characteristics and operative management of paediatric extravasation injuries. Our review highlighted three key issues. Firstly, surgical management is commonly reported in the literature in cases where there is significant soft tissue injury but as there are no comparative studies, it is unclear whether this is optimal. Secondly, there are no data to support one surgical management approach over another, or over no treatment. Thirdly, there are no data on the adverse events caused by the different surgical interventions.

A variation of the Gault method was used in the largest number of children to receive surgical treatment across the included studies. Gault [4] described a retrospective case series of 96 patients, ranging in age from newborn to 70 years, who were split into two groups: those seen within 24 hours of the extravasation and those seen later than this. Patients seen within 24 hours had treatment as per the Gault method while those seen after 24 hours had no treatment. The original Gault paper was not included in our review because there were no separate paediatric data available but a separate risk of bias assessment by two authors rated the study as 'poor' due to the lack of a clearly defined objective or population, subjects that were not comparable and an inadequate length of follow-up. [Table 6] Despite the inherent limitations in the original Gault study, the method he described is widely used, as demonstrated by this review where it was a commonly described technique to manage extravasation injuries. This approach is not without downsides: it is an invasive procedure with associated morbidity such as pain and possible tendon and nerve damage, and takes considerable time to perform.

Evidence for the continuing use of the "Gault technique"

The degree of injury was recorded for 13% of the children included in the Gault technique studies, 93% of those in the debridement and further surgery group and 15% of those in the hyaluronidase injection group. These figures demonstrate some of the difficulties faced in drawing comparisons between different

For peer review only - http://bmjopen.bmj.com/site/about/guidelines.xhtml

interventions, both due to the poor recording of injuries and because of the fact that accurate documentation of injury severity appears to be more consistently applied following more severe injuries.

It was not possible to perform pooled analysis from included studies of Gault and alternative techniques due to study heterogeneity and incomplete outcome reporting.

All but one of the included studies were case series and the one before-and-after study [31] primarily examined the effect of new extravasation treatment guidelines on knowledge of hyaluronidase, extravasation incident reporting and time from extravasation discovery to treatment. Therefore, even though they found similar harm scores for the hyaluronidase and the non-hyaluronidase groups, the results could not be relied upon to assess the effectiveness of the intervention.

In addition, inclusion criteria varied considerably, details of the injuries were poorly described, and no studies made direct comparison to another intervention. Finally, detailed outcome measures following interventions were generally lacking. In summarizing the included studies, it can be noted that despite widespread use, there is no high-quality evidence to support the Gault approach or its modifications.

The future of extravasation management

A key conclusion from this review is that resolution of extravasation injuries is common regardless of the treatment applied. In the absence of data to support one treatment modality over another, it is imperative that high quality methodology randomized comparisons are undertaken; however, given the high rate of resolution with conservative treatment, any such trial will require a large, collaborative and multidisciplinary approach. Although many of the in the included studies required minimal intervention, full-thickness skin injury was commonplace; this has the potential for lifelong scarring in patients with an immature skin barrier such as children and preterm infants in particular. More serious complications were rare in but given that only a minority of studies reported on longer-term outcomes, these may underestimate the true burden of extravasation injuries. We suggest the creation of a core outcome set for extravasation injury treatment and that future studies use longer-term follow-up to monitor those outcomes.

For peer review only - http://bmjopen.bmj.com/site/about/guidelines.xhtml

The use of a universal grading scheme (such as Millam's [43]) to assess the initial extent of injuries would also aid the comparison between different interventions. However, this requires meticulous documentation of the appearance of the extravasated site as well as the extent of tissue damage.

This review was limited by the low methodological quality of included studies and inconsistent outcome reporting. Variability in baseline data, intervention data and outcome data precluded formal meta-analysis. These limitations prevent the authors from providing definitive clinical recommendations either for or against paediatric extravasation, based on the current evidence.

Conclusion

There is a paucity of evidence to inform surgical treatment of paediatric and neonatal extravasation injuries. Gault's technique is a commonly described approach, but we did not identify any evaluation of its effectiveness compared to other approaches or conservative management. A centralized extravasation register, use of a standardized and agreed grading scheme, development of a core outcome set and adequate follow-up of extravasation injuries are required to provide incidence and outcome data for this condition, and to inform much-needed, definitive trials of therapeutic approaches to extravasation injuries in children and neonates.

For peer review only

Author Contributions

Max Little was one of the authors to independently assess eligibility of the title and abstracts of the search results. He then aided in extraction of the relevant data from included studies, performed the risk of bias assessments and drafted all of the versions of the article leading up to this submission.

Sophie Dupré also aided in the extraction of the relevant data from included studies, performed the risk of bias assessments and assisted in the drafting of each version of the article.

Justin Wormald made substantial contributions to the design of the study, aided in the extraction of data and in their analysis and interpretation, as well as reviewing and critically editing each version of the article.

Matthew Gardiner made substantial contributions to the design of the study as well as reviewing and critically editing each version of the article.

Chris Gale acted as one of the co-senior authors, lending his expertise in paediatrics. He reviewed and critically edited the later versions of the article, substantially changing the text and ensuring its accuracy.

Abhilash Jain acted as the other co-senior author, lending his expertise in plastic surgery. He made substantial contributions to the design of the study, aided in the analysis and interpretation of the data from included studies and critically reviewed each version of the article.

Funding: JW is an NIHR Academic Clinical Fellow; Chris Gale is funded by a Medical Research Council (MRC) Clinician Scientist Fellowship. This research is supported by the National Institute for Health Research (NIHR) infrastructure at NDORMS. The views expressed are those of the author(s) and not necessarily those of the NHS, the NIHR or the Department of Health. No funding was directly received for the development of this manuscript.

Conflict of interest: CG has received support from Chiesi Pharmaceuticals to attend an educational conference; in the past 5 years he been investigator on received research grants from Medical Research Council, National Institute of Health Research, Canadian Institute of Health Research, Department of Health in England, Mason Medical Research Foundation, Westminster Medical School Research Trust and Chiesi Pharmaceuticals; he declares no other conflict of interest.

For peer review only

References

1. Doellman D, Hadaway L, Bowe-Geddes LA, et al. Infiltration and extravasation: Update on prevention and management. *J Infus Nurs*. 2009;32(4):203-211. doi:10.1097/NAN.0b013e3181aac042

2. Paquette V, McGloin R, Northway T, et al. Describing intravenous extravasation in children (DIVE study). *Can J Hosp Pharm*. 2011;64(5):340-345.

3. Wilkins C, Emmerson A. Extravasation injuries on regional neonatal units. *Arch Dis Child Fetal neonatal Ed*. 2004;89(3).

4. Murphy A, Gilmour R, Coombs C. Extravasation injury in a paediatric population. *ANZ J Surg*. 2017.

5. Gault D. Extravasation injuries. *Br J Plast Surg*. 1993;46(2):91.

6. Corbett M, Marshall D, Harden M, et al. Treatment of extravasation injuries in infants and young children: a scoping review and survey. Southampton (UK): NIHR Journals Library. *Health Technol Assess (Rockv)*. 2018;22(46). doi:10.3310/hta22460

7. Moher D, Liberati A, Tetziaff J, et al. Preferred Reporting Items for Systematic Reviews and Meta-Analyses. The PRISMA Statement. *PLoS Med*. 2009;6(7):e1000097. doi:10.1371/journal.pmed1000097

8. Higgins J, Green S. Cochrane Handnook for Systematic Reviews of Interventions Version 5.1.0 [updated March 2011]. *Cochrane Collab*. 2011. http://handbook.cochrane.org.

9. NIH Study Quality Assessment Tools. https://www.nhlbi.nih.gov/health-topics/study-quality-assessment-tools. Accessed March 25, 2019.

10. Falcone P, Barrall D, Jeyarajah D, et al. Nonoperative management of full-thickness intravenous extravasation injuries in premature neonates using enzymatic debridementtle. *Ann Plast Surg*. 1989;22(2):146.

11. Casanova D, Bardot J, Magalon G. Emergency treatment of accidental infusion leakage in the newborn: Report of 14 cases. *Br J Plast Surg*. 2001;54(5):396-399.

12. Cochran T, Bomyea K, Kahn M. Treatment of iodinated contrast material extravasation with hyaluronidase. *Acad Radiol*. 2002;9.

13. Cho K, Lee S, Burm J, et al. Successful combined treatment with total parenteral nutrition fluid extravasation injuries in preterm infants. *J Korean Med Sci*. 2007;22(3):588.

14. Onesti M, Carella S, Maruccia M, et al. The use of hyalomatrix PA in the treatment of extravasation affecting premature neonates. *Plast Reconstr Surg*. 2012;129(1):219e-21e.

15. Firat C, Erbatur S, Aytekin A. Management of extravasation injuries: a retrospective study. *J Plast Surg Hand Surg*. 2013;47(1):60.

16. Ching L, Wong K, Milroy C. Paediatric extravasation injuries: a review of 69 consecutive

For peer review only - http://bmjopen.bmj.com/site/about/guidelines.xhtml

patients. *Int J Surg*. 2014;12(10):1036.

17. Sivrioğlu N, Irkören S. Versajet hydrosurgery system in the debridement of skin necrosis after calcium gluconate extravasation: report of 9 infantile cases. *Acta Orthop Traumatol Turc*. 2014;48(1):6.

18. Yan Y, Fan Q, Li A, et al. Treatment of cutaneous injuries of neonates induced by drug extravasation with hyaluronidase and hirudoid. *Iran J Pediatr*. 2014;24(4):352-358.

19. Ghanem M, Mansour A, Exton R, et al. Childhood extravasation injuries: improved outcome following the introduction of hospital-wide guidelines. *J Plast Reconstr Aesthetic Surg*. 2015;68(4):505.

20. Kostogloudis N, Demiri E, Tsimponis A, et al. Severe Extravasation Injuries in Neonates: A Report of 34 Cases. *Pediatr Dermatol*. 2015;32(6):830.

21. Sung K-Y, Lee S-Y. Nonoperative Management of Extravasation Injuries Associated With Neonatal Parenteral Nutrition Using Multiple Punctures and a Hydrocolloid Dressing. *Wounds A Compend Clin Res Pract*. 2016;28(5):145-152.

22. Odom B, Lowe L, Yates C. Peripheral Infiltration and Extravasation Injury Methodology: A Retrospective Study. *J Infus Nurs*. 2018;41(4):247-252.

23. Pacheco Compaña F, Midón Míguez J, de Toro Santos F. Lesions Associated With Calcium Gluconate Extravasation. *Ann Plast Surg*. 2017;79(5):444-449.

24. Harris P, Bradley S, Moss A. Limiting the damage of iatrogenic extravasation injury in neonates. *Plast Reconstr Surg*. 2001;107(3):893-894. doi:https://doi.org/10.1097/00006534-200103000-00053

25. Andrés A, Burgos L, López Gutiérrez J, et al. Treatment protocol for extravasation lesions. *Cir Pediatr*. 2006;19(3):136-139.

26. Linder R, Upton J, Osteen R. Management of extensive doxorubicin hydrochloride extravasation injuries. *J Hand Surg Am*. 1983;8(1):32-38. doi:https://doi.org/10.1016/S0363-5023(83)80048-3

27. Linder R, Upton J. Prevention of extravasation injuries secondary to doxorubicin. *Postgrad Med J*. 1985;77(4):105-9,112,114. doi:https://doi.org/10.1080/00325481.1985.11698920

28. Upton J, Mulliken J, Murray J. Major intravenous extravasation injuries. *Am J Surg*. 1979;137(4):497-506. doi:https://doi.org/10.1016/0002-9610(79)90121-1

29. von Heimburg D, Pallua N. Early and late treatment of iatrogenic injection damage. *Chirurg*. 1998;69(12):1378-1382. doi:https://doi.org/10.1007/s001040050588

30. Weiss Y, Ackerman C, Shmilovitz L. Localized necrosis of scalp in neonates due to calcium gluconate infusions: a cautionary note. *Pediatrics*. 1975;56(6):1084-1086.

31. Hanrahan K. Hyaluronidase for treatment of intravenous extravasations: implementation of an evidence-based guideline in a pediatric population. *J Spec Pediatr Nurs*.

For peer review only - http://bmjopen.bmj.com/site/about/guidelines.xhtml

2013;18(3):253-262. doi:https://doi.org/10.1111/jspn.12035

32. Boyar V, Galiczewski C. Efficacy of Dehydrated Human Amniotic Membrane Allograft for the Treatment of Severe Extravasation Injuries in Preterm Neonates. *Wounds*. 2018;30(8):224-228.

33. Yan Y, Gong M, Chen J, et al. Incidence, risk factors and treatment outcomes of drug extravasation in pediatric patients in China. *Turk J Pediatr*. 2017;(59):162-168.

34. Myers P, Krasniak P, Bell D. Managing intravenous infiltration injuries in the neonatal intensive care unit. *J Burn Care Res*. 2018;39(9):S151.

35. Loth T, Eversmann W. Extravasation injuries in the upper extremity. *Clin Orthop Relat Res*. 1991;(272):48.

36. Scuderi N, Onesti M. Antitumor agents: extravasation, management, and surgical treatment. *Ann Plast Surg*. 1994;32(1):39.

37. Langstein N, Duman H, Seelig D, et al. Retrospective study of the management of chemotherapeutic extravasation injury. *Ann Plast Surg*. 2002;49(4):369.

38. D'Andrea F, Onesti M, Nicoletti G, et al. Surgical treatment of ulcers caused by extravasation of cytotoxic drugs. *Scand J Plast Reconstr Surg Hand Surg*. 2004;38(5):288-292.

39. Rose R, Felix R, Crawford-Sykes A, et al. Extravasation injuries. *West Indian Med J*. 2008;57(1):40.

40. Sbitany H, Koltz F, Mays C, et al. CT contrast extravasation in the upper extremity: strategies for management. *Int J Surg*. 2010;8(5):384.

41. Kim S, Cook K, Lee I, et al. Computed tomography contrast media extravasation: treatment algorithm and immediate treatment by squeezing with multiple slit incisions. *Int Wound J*. 2017;14(2):430-434.

42. Onesti M, Carella S, Fioramonti P, et al. Chemotherapy Extravasation Management: 21-Year Experience. *Ann Plast Surg*. 2017;79(5):450-457.

43. Millam D. Managing complications of IV therapy. *Nursing (Lond)*. 1988;(18):34-43.

44. Flemmer L, Chan J. A pediatric protocol for management of extravasation injuries. *Pediatr Nurs*. 1993;18:44-47.

For peer review only

Search Results

Table of Contents

Search History .... page 2

1. Thoracic and abdominal extravasation: a complication of hyperalimentation in infants. .... page 4

2. [Traumatic cerebral aneurysm showing remarkable extravasation into the lateral ventricle--report of a case in an infant (author's transl)]. .... page 4

3. Urinary ascites in children owing to extravasation of urine from the bladder. .... page 4

4. Bone scintigraphy for neonatal osteomyelitis: simulation by extravasation of intravenous calcium. .... page 4

5. Perirenal urinary extravasation associated with urethral valves in infants. .... page 4

6. Nafcillin extravasation injury. Use of hyaluronidase as an antidote. .... page 4

7. Cutaneous calcinosis in a neonate following extravasation of calcium gluconate. .... page 4

8. Urinary extravasation in the newborn male with posterior urethral valves. .... page 4

9. Intrathoracic extravasation of sclerosing agents associated with central venous catheters. .... page 5

10. Nonoperative management of full-thickness intravenous extravasation injuries in premature neonates using enzymatic debridement. .... page 5

For peer review only

Search History

1. AMED, EMBASE, Medline, CINAHL; p?ediatric*.ti,ab; 2702402 results.
2. AMED, EMBASE, Medline, CINAHL; neonat*.ti,ab; 508216 results.
3. AMED, EMBASE, Medline, CINAHL; infant*.ti,ab; 761110 results.
4. AMED, EMBASE, Medline, CINAHL; child.ti,ab; 737276 results.
5. AMED, EMBASE, Medline, CINAHL; 1 OR 2 OR 3 OR 4; 4209881 results.
6. AMED, EMBASE, Medline, CINAHL; extra?vasat*.ti,ab; 19189 results.
7. AMED, EMBASE, Medline, CINAHL; extravasation.ti,ab; 30514 results.
8. AMED, EMBASE, Medline, CINAHL; 6 OR 7; 32931 results.
9. AMED, EMBASE, Medline, CINAHL; 5 AND 8; 1849 results.
10. AMED, EMBASE, Medline, CINAHL; outcome*.ti,ab; 2946939 results.
11. AMED, EMBASE, Medline, CINAHL; PROM*.ti,ab; 2921492 results.
12. AMED, EMBASE, Medline, CINAHL; patient?related*.ti,ab; 131 results.
13. AMED, EMBASE, Medline, CINAHL; patient?report*.ti,ab; 689 results.
14. AMED, EMBASE, Medline, CINAHL; 10 OR 11 OR 12 OR 13; 5654365 results.
15. AMED, EMBASE, Medline, CINAHL; conservative*.ti,ab; 231035 results.
16. AMED, EMBASE, Medline, CINAHL; non?operative*.ti,ab; 11082 results.
17. AMED, EMBASE, Medline, CINAHL; non?surg*.ti,ab; 14737 results.
18. AMED, EMBASE, Medline, CINAHL; cold*.ti,ab; 225847 results.
19. AMED, EMBASE, Medline, CINAHL; cool*.ti,ab; 101309 results.
20. AMED, EMBASE, Medline, CINAHL; hot*.ti,ab; 160999 results.
21. AMED, EMBASE, Medline, CINAHL; warm*.ti,ab; 110969 results.
22. AMED, EMBASE, Medline, CINAHL; elevat*.ti,ab; 1359688 results.
23. AMED, EMBASE, Medline, CINAHL; dress*.ti,ab; 53707 results.
24. AMED, EMBASE, Medline, CINAHL; 15 OR 16 OR 17 OR 18 OR 19 OR 20 OR 21 OR 22 OR 23; 2170626 results.
25. AMED, EMBASE, Medline, CINAHL; medic*.ti,ab; 3892625 results.
26. AMED, EMBASE, Medline, CINAHL; steroid*.ti,ab; 449723 results.
27. AMED, EMBASE, Medline, CINAHL; cortico?steroid*.ti,ab; 114994 results.
28. AMED, EMBASE, Medline, CINAHL; corticosteroid*.ti,ab; 206312 results.
29. AMED, EMBASE, Medline, CINAHL; sub?cut*.ti,ab; 245741 results.
30. AMED, EMBASE, Medline, CINAHL; subcut*.ti,ab; 189955 results.
31. AMED, EMBASE, Medline, CINAHL; inject*.ti,ab; 1404121 results.
32. AMED, EMBASE, Medline, CINAHL; topical*.ti,ab; 199318 results.
33. AMED, EMBASE, Medline, CINAHL; cream*.ti,ab; 38332 results.
34. AMED, EMBASE, Medline, CINAHL; ointment*.ti,ab; 24526 results.
35. AMED, EMBASE, Medline, CINAHL; emollient*.ti,ab; 3696 results.
36. AMED, EMBASE, Medline, CINAHL; antidote*.ti,ab; 11910 results.
37. AMED, EMBASE, Medline, CINAHL; Sodium?thiosulfate*.ti,ab; 6 results.
38. AMED, EMBASE, Medline, CINAHL; mechlorethamine*.ti,ab; 1486 results.
39. AMED, EMBASE, Medline, CINAHL; Dexrazoxane*.ti,ab; 1175 results.
40. AMED, EMBASE, Medline, CINAHL; N?acetylcysteine*.ti,ab; 1604942 results.
41. AMED, EMBASE, Medline, CINAHL; vitamin?C*.ti,ab; 422 results.
42. AMED, EMBASE, Medline, CINAHL; ascorbic*.ti,ab; 58559 results.
43. AMED, EMBASE, Medline, CINAHL; basic AND fibroblast AND growth AND factor.ti,ab; 29979 results.
44. AMED, EMBASE, Medline, CINAHL; granulocyte AND macrophage AND colony AND stimulating AND factor.ti,ab; 37389 results.
45. AMED, EMBASE, Medline, CINAHL; hyaluroni*.ti,ab; 42841 results.
46. AMED, EMBASE, Medline, CINAHL; salin*.ti,ab; 375104 results.
47. AMED, EMBASE, Medline, CINAHL; 25 OR 26 OR 27 OR 28 OR 29 OR 30 OR 31 OR 32 OR 33 OR 34 OR 35 OR 36 OR 37 OR 38 OR 39 OR 40 OR 41 OR 42 OR 43 OR 44 OR 45 OR 46; 7833762 results.
48. AMED, EMBASE, Medline, CINAHL; squeeze*.ti,ab; 9290 results.
49. AMED, EMBASE, Medline, CINAHL; surg*.ti,ab; 3592311 results.
50. AMED, EMBASE, Medline, CINAHL; intervention*.ti,ab; 1719913 results.
51. AMED, EMBASE, Medline, CINAHL; irrigation*.ti,ab; 45442 results.
52. AMED, EMBASE, Medline, CINAHL; excis*.ti,ab; 326226 results.
53. AMED, EMBASE, Medline, CINAHL; infiltrat*.ti,ab; 404118 results.
54. AMED, EMBASE, Medline, CINAHL; therapeut*.ti,ab; 1901755 results.
55. AMED, EMBASE, Medline, CINAHL; manage*.ti,ab; 2431717 results.
56. AMED, EMBASE, Medline, CINAHL; treatment*.ti,ab; 8388485 results.

For peer review only

57. AMED, EMBASE, Medline, CINAHL; wash?out*.ti,ab; 28666 results.
58. AMED, EMBASE, Medline, CINAHL; operat*.ti,ab; 2000359 results.
59. AMED, EMBASE, Medline, CINAHL; bedside*.ti,ab; 57042 results.
60. AMED, EMBASE, Medline, CINAHL; liposuct*.ti,ab; 5719 results.
61. AMED, EMBASE, Medline, CINAHL; fasciotom*.ti,ab; 5310 results.
62. AMED, EMBASE, Medline, CINAHL; 48 OR 49 OR 50 OR 51 OR 52 OR 53 OR 54 OR 55 OR 56 OR 57 OR 58 OR 59 OR 60 OR 61; 15163731 results.
63. AMED, EMBASE, Medline, CINAHL; 9 AND 24; 222 results.
64. AMED, EMBASE, Medline, CINAHL; 9 AND 47; 982 results.
65. AMED, EMBASE, Medline, CINAHL; 9 AND 62; 1130 results.
66. AMED, EMBASE, Medline, CINAHL; 24 OR 47 OR 62; 20658308 results.
67. AMED, EMBASE, Medline, CINAHL; 9 AND 66; 1455 results.
68. AMED, EMBASE, Medline, CINAHL; 67 NOT animal; 1176 results.
69. AMED, EMBASE, Medline, CINAHL; 68 NOT laboratory; 1117 results.
70. AMED, EMBASE, Medline, CINAHL; 69 NOT adult; 804 results.

For peer review only

For peer review only - http://bmjopen.bmj.com/site/about/guidelines.xhtml

1. Thoracic and abdominal extravasation: a complication of hyperalimentation in infants.

Citation: AJR. American journal of roentgenology, Mar 1977, vol. 128, no. 3, p. 419-422, 0361-803X (March 1977)

Author(s): Spriggs, D W; Brantley, R E

Source: Medline

2. [Traumatic cerebral aneurysm showing remarkable extravasation into the lateral ventricle--report of a case in an infant (author's transl)].

Citation: No shinkei geka. Neurological surgery, Apr 1977, vol. 5, no. 4, p. 371-378, 0301-2603 (April 1977)

Author(s): Nakamura, T; Matsuoka, Y; Nishimura, S

Source: Medline

3. Urinary ascites in children owing to extravasation of urine from the bladder.

Citation: The Journal of urology, Sep 1979, vol. 122, no. 3, p. 409-411, 0022-5347 (September 1979)

Author(s): Redman, J F; Seibert, J J; Arnold, W

Source: Medline

4. Bone scintigraphy for neonatal osteomyelitis: simulation by extravasation of intravenous calcium.

Citation: Radiology, Apr 1980, vol. 135, no. 1, p. 185-186, 0033-8419 (April 1980)

Author(s): Balsam, D; Goldfarb, C R; Stringer, B; Farruggia, S

Source: Medline

5. Perirenal urinary extravasation associated with urethral valves in infants.

Citation: The Journal of urology, Nov 1980, vol. 124, no. 5, p. 688-691, 0022-5347 (November 1980)

Author(s): Mitchell, M E; Garrett, R A

Source: Medline

6. Nafcillin extravasation injury. Use of hyaluronidase as an antidote.

Citation: American journal of diseases of children (1960), Dec 1981, vol. 135, no. 12, p. 1113-1114, 0002-922X (December 1981)

Author(s): Zenk, K E; Dungy, C I; Greene, G R

Source: Medline

7. Cutaneous calcinosis in a neonate following extravasation of calcium gluconate.

Citation: Journal of the American Academy of Dermatology, Mar 1982, vol. 6, no. 3, p. 392-395, 0190-9622 (March 1982)

Author(s): Hironaga, M; Fujigaki, T; Tanaka, S

Source: Medline

8. Urinary extravasation in the newborn male with posterior urethral valves.

Citation: Journal of pediatric surgery, Dec 1982, vol. 17, no. 6, p. 751-756, 0022-3468 (December 1982)

Author(s): Greenfield, S P; Hensle, T W; Berdon, W E; Geringer, A M

For peer review only

Source: Medline

9. Intrathoracic extravasation of sclerosing agents associated with central venous catheters.

Citation: The American journal of pediatric hematology/oncology, Jan 1988, vol. 10, no. 3, p. 249-251, 0192-8562 (1988)

Author(s): Watterson, J; Heisel, M; Cich, J A; Priest, J R

Source: Medline

Full Text: Available from *Ovid* in *American Journal of Pediatric Hematology/Oncology*

10. Nonoperative management of full-thickness intravenous extravasation injuries in premature neonates using enzymatic debridement.

Citation: Annals of plastic surgery, Feb 1989, vol. 22, no. 2, p. 146-149, 0148-7043 (February 1989)

Author(s): Falcone, P A; Barrall, D T; Jeyarajah, D R; Grossman, J A

Source: Medline

Full Text: Available from *Ovid* in *Annals of Plastic Surgery*

For peer review only

Search Results

Table of Contents

Search History .... page 2

1. Hepatic and splenic blush on computed tomography in children following blunt abdominal trauma: Is intervention necessary? .... page 3

2. Safety and effectiveness of intravenous pentamidine for prophylaxis of Pneumocystis jirovecii pneumonia in pediatric hematology/oncology patients .... page 3

3. Efficacy, Safety, and Pharmacokinetics of a New 10 % Liquid Intravenous Immunoglobulin Containing High Titer Neutralizing Antibody to RSV and Other Respiratory Viruses in Subjects with Primary Immunodeficiency Disease .... page 3

4. A multicenter, randomized, observer-blinded, active-controlled study evaluating the safety and effectiveness of ceftaroline compared with ceftriaxone plus vancomycin in pediatric patients with complicated community-acquired bacterial pneumonia .... page 3

5. Predictive factors for conservative treatment failure in grade IV pediatric blunt renal trauma .... page 3

6. Clinical negligence claims in pediatric surgery in England: Pattern and trends .... page 3

7. Outcomes of peripherally inserted double lumen central catheter in very low birth weight infants .... page 4

8. Pelvic Artery Embolization in the Management of Obstetrical Hemorrhage: Predictive Factors for Clinical Outcomes .... page 4

9. The role of interventional radiology for pediatric blunt renal trauma .... page 4

10. Safety and efficacy of gadoteric acid in pediatric magnetic resonance imaging: overview of clinical trials and post-marketing studies .... page 4

For peer review only

For peer review only - http://bmjopen.bmj.com/site/about/guidelines.xhtml

Evidence Services | library.nhs.uk

Search History

1. EMBASE; exp PEDIATRICS/; 87850 results.
2. EMBASE; exp CHILD/; 2256483 results.
3. EMBASE; exp INFANT/; 930470 results.
4. EMBASE; exp ADOLESCENT/; 1367630 results.
5. EMBASE; exp NEWBORN/; 495069 results.
6. EMBASE; exp CONTRAST MEDIUM EXTRAVASATION/ OR exp DRUG EXTRAVASATION/ OR exp EXTRAVASATION/ OR exp INJECTION SITE EXTRAVASATION/; 11979 results.
7. EMBASE; exp OUTCOME MEASUREMENT/ OR exp OUTCOMES RESEARCH/ OR exp EVIDENCE BASED MEDICINE/ OR exp TREATMENT OUTCOME/; 1809683 results.
8. EMBASE; 1 OR 2 OR 3 OR 4 OR 5; 2973897 results.
9. EMBASE; 6 AND 8; 958 results.
10. EMBASE; 7 AND 9; 150 results.

For peer review only

1. Hepatic and splenic blush on computed tomography in children following blunt abdominal trauma: Is intervention necessary?

**Citation:**	Journal of Trauma and Acute Care Surgery, 2016, vol./is. 81/2(266-270), 2163-0755;2163-0763 (2016)
**Author(s):**	Ingram M.-C.E.; Siddharthan R.V.; Morris A.D.; Hill S.J.; Travers C.D.; McKracken C.E.; Heiss K.F.; Raval M.V.; Santore M.T.
**Source:**	EMBASE
**Full Text:**	Available from *Ovid* in *Journal of Trauma and Acute Care Surgery*

2. Safety and effectiveness of intravenous pentamidine for prophylaxis of Pneumocystis jirovecii pneumonia in pediatric hematology/oncology patients

**Citation:**	Journal of Pediatric Hematology/Oncology, July 2016, vol./is. 38/6(e180-e185), 1077-4114;1536-3678 (26 Jul 2016)
**Author(s):**	Solodokin L.J.; Klejmont L.M.; Scipione M.R.; Dubrovskaya Y.; Lighter-Fisher J.; Papadopoulos J.
**Source:**	EMBASE
**Full Text:**	Available from *Ovid* in *Journal of Pediatric Hematology/Oncology*

3. Efficacy, Safety, and Pharmacokinetics of a New 10 % Liquid Intravenous Immunoglobulin Containing High Titer Neutralizing Antibody to RSV and Other Respiratory Viruses in Subjects with Primary Immunodeficiency Disease

**Citation:**	Journal of Clinical Immunology, August 2016, vol./is. 36/6(590-599), 0271-9142;1573-2592 (01 Aug 2016)
**Author(s):**	Wasserman R.L.; Lumry W.; Harris J.; Levy R.; Stein M.; Forbes L.; Cunningham-Rundles C.; Melamed I.; Kobayashi A.L.; Du W.; Kobayashi R.
**Source:**	EMBASE
**Full Text:**	Available from *Springer Link Journals* in *Journal of Clinical Immunology*

4. A multicenter, randomized, observer-blinded, active-controlled study evaluating the safety and effectiveness of ceftaroline compared with ceftriaxone plus vancomycin in pediatric patients with complicated community-acquired bacterial pneumonia

**Citation:**	Pediatric Infectious Disease Journal, July 2016, vol./is. 35/7(760-766), 0891-3668;1532-0987 (01 Jul 2016)
**Author(s):**	Blumer J.L.; Ghonghadze T.; Cannavino C.; O'Neal T.; Jandourek A.; Friedland H.D.; Bradley J.S.
**Source:**	EMBASE
**Full Text:**	Available from *Ingenta* in *Pediatric Infectious Disease Journal, The*<br>Available from *Ovid* in *Pediatric Infectious Disease Journal*

5. Predictive factors for conservative treatment failure in grade IV pediatric blunt renal trauma

**Citation:**	Journal of Pediatric Urology, April 2016, vol./is. 12/2(93.e1-93.e7), 1477-5131;1873-4898 (01 Apr 2016)
**Author(s):**	Lee J.N.; Lim J.K.; Woo M.J.; Kwon S.Y.; Kim B.S.; Kim H.T.; Kim T.-H.; Yoo E.S.; Chung S.K.
**Source:**	EMBASE

6. Clinical negligence claims in pediatric surgery in England: Pattern and trends

**Citation:**	European Journal of Pediatric Surgery, February 2015, vol./is. 25/1(66-70), 0939-7248;1439-359X (February 2015)

For peer review only

Author(s): Thyoka M.

Source: EMBASE

7. Outcomes of peripherally inserted double lumen central catheter in very low birth weight infants

Citation: Journal of Neonatal-Perinatal Medicine, March 2016, vol./is. 9/1(99-105), 1934-5798;1878-4429 (24 Mar 2016)

Author(s): Cheong S.M.; Totsu S.; Nakanishi H.; Uchiyama A.; Kusuda S.

Source: EMBASE

8. Pelvic Artery Embolization in the Management of Obstetrical Hemorrhage: Predictive Factors for Clinical Outcomes

Citation: CardioVascular and Interventional Radiology, December 2015, vol./is. 38/6(1477-1486), 0174-1551;1432-086X (01 Dec 2015)

Author(s): Zhang E.; Liu L.; Owen R.

Source: EMBASE

Full Text: Available from *Springer Link Journals* in *CardioVascular and Interventional Radiology*

9. The role of interventional radiology for pediatric blunt renal trauma

Citation: Italian Journal of Pediatrics, October 2015, vol./is. 41/1(no pagination), 1720-8424;1824-7288 (15 Oct 2015)

Author(s): Lin W.-C.; Lin C.-H.

Source: EMBASE

Full Text: Available from *National Library of Medicine* in *Italian Journal of Pediatrics*
Available from *BioMed Central* in *Italian Journal of Pediatrics*
Available from *National Library of Medicine* in *Italian Journal of Pediatrics*
Available from *ProQuest* in *Italian Journal of Pediatrics*

10. Safety and efficacy of gadoteric acid in pediatric magnetic resonance imaging: overview of clinical trials and post-marketing studies

Citation: Pediatric Radiology, November 2015, vol./is. 45/12(1831-1841), 0301-0449;1432-1998 (01 Nov 2015)

Author(s): Balassy C.; Roberts D.; Miller S.F.

Source: EMBASE

Full Text: Available from *Springer Link Journals* in *Pediatric Radiology*

For peer review only

Search Results

heRsr ul tu1.r1.E

]reiAn ,yE.uix gggggggggggggggggggggggggggggggggggggggggggggggggggggggggggggggggggggggggggggggggggg

cg 5Criar1Ax .ire.Cr1. ul eAAyor1.es y1lwEyu1 sre-ear y1 .nr 1r(Rui1g gggggggggggggggggggggggggggggggggggggggggg

mg tnysonuuo rf.iedeEe.yu1 y1kwiyrEb yC:iudro uw.AuCr lussu(y1a .nr y1.iuowA.yu1 ul nuE:y.es)(yor awyorsy1rEg gggggggggggggggggggggggggggggggggggggggggggggggggggggggggggggggggggggggggggggggggggggggggggggggear 0ggggggggggg

0g jhire.Cr1. :iu.uAus lui rf.iedeEe.yu1 srEyu1E g gggggggggggggggggggggggggggggggggggggggggggggggggggggggggg

Ng hnr wEr ul Ar1.ies dr1uwE Ae.nr.riE y1 :eroye.iyA u1Ausuax))e Aew.yu1eix .esrg gggggggggggggggggggggggggggggg

Fg T 1r(e::iueAn .u Ce1earCr1. ul y1.iedr1uwE y1lys.ie.yu1 y1 :roye.iyA :e.yr1.Eb :e.nu:nxEyusuax2 AseEEylyAe.yu12 e1o .ire.Cr1.g gggggggggggggggggggggggggggggggggggggggggggggggggggggggggggggggggggggggggggggggggggggggg

9g]esy1r yiiyae.yu1 lui .nr Ce1earCr1. ul E-y1 rf.iedeEe.yu1 y1kwix y1 1ru1e.rEg gggggggggggggggggggggggggggggggggggg

g 5f.iedeEe.yu1 y1kwiyrEg gggggggggggggggggggggggggggggggggggggggggggggggggggggggggggggggggggggggggggggg

vg jtr1.ies dr1uwE Ae.nr.riE y1 1r(Rui1 y1le1.Eg pui- 3iuw: Seir1.ries [w.iy.yu1 ul .nr Sriy1e.usuax]rA.yu1 ul .nr 8w.An Sroye.iyA]uAyr.x g ggggggggggggggggggggggggggggggggggggggggggggggggggggggggggggggggggggggg

4g 5f.iedeEe.yu1 ul :eir1.ries esyCr1.e.yu1 lswyo y1.u .nr ir1es :rsdyE))e AuC:syAe.yu1 ul Ar1.ies dr1uwE Ae.nr.ri y1 e 1ru1e.rg ggggggggggggggggggggggggggggggggggggggggggggggggggggggggggggggggggggggggggggggggggggggg

c g SriAw.e1ruwE Ar1.ies sy1r rf.iedeEe.yu1 CeE wrieoy1a eE e1 eREArEEg gggggggggggggggggggggggggggggggggggggg

For peer review only

Search History

1. Medline; exp PEDIATRICS/; 49428 results.
2. Medline; exp CHILD/; 1666146 results.
3. Medline; exp INFANT/; 1008023 results.
4. Medline; exp INFANT, NEWBORN/; 535610 results.
5. Medline; 1 OR 2 OR 3 OR 4; 2185064 results.
6. Medline; exp EXTRAVASATION OF DIAGNOSTIC AND THERAPEUTIC MATERIALS/; 3098 results.
7. Medline; exp "OUTCOME ASSESSMENT (HEALTH CARE)"/ OR exp PATIENT OUTCOME ASSESSMENT/ OR exp TREATMENT OUTCOME/; 815946 results.
8. Medline; exp EARLY MEDICAL INTERVENTION/; 1559 results.
9. Medline; exp THERAPEUTIC IRRIGATION/ OR exp THERAPEUTICS/; 5011881 results.
10. Medline; exp SPECIALTIES, SURGICAL/ OR exp SURGICAL PROCEDURES, OPERATIVE/; 2809893 results.
11. Medline; 8 OR 9 OR 10; 6354238 results.
12. Medline; 5 AND 6; 345 results.
13. Medline; 7 AND 12; 36 results.
14. Medline; 11 AND 12; 248 results.
15. Medline; 12 OR 13 OR 14; 345 results.

For peer review only

1. **Emergency treatment of accidental infusion leakage in the newborn.**

Citation: iy.yEn kuwi1es ul :seE.yA Ewiarix2 e1 m m2 dusg FF2 1ug c2 :g v4g2)cmm9 m m

Author(s): GuEr2 yA.uiye7 hwi1ri2 6e..nr(7 ,eiiyE2 Sews T7 6uEE2 T1.nu1x D ,

Source: 6rosy1r

Full Text: TdeyseRsr liuGSear y1chRsual Rtr 1.lEtsumgluae. ngmy.mo

2. **Childhood extravasation injuries: improved outcome following the introduction of hospital-wide guidelines.**

Citation: uwi1es ul :seE.yA2 irAu1E.iwA.ydr erE.nr.yA Ewiarix b SGT]2 T:i m cF2 dusg 9v2 1ug F F)Fcv2 cv v) F04 T:iys m cF

Author(s): 3ne1rC2 Tsy 67 6e1Euwi2 TRowsieR7 5f.u12 GrRrAAe7 Su(rss2 u1e.ne17 6eEnneoy2]xro7 wsE.iuor2 [rys7]Cy.n2 3yssye1

Source: 6rosy1r

3. **[Treatment protocol for extravasation lesions].**

Citation: tyiway e :roye .iyAe b uiae1u ulyAyes or se]uAyroeo 5E:e1 use or tyiway e Sroye .iyAe2 m 92 dusg c42 1ug 02 :g c09)c042 mcN)cmmc wsx m 9

Author(s): T1oi E2 T 67 wiauE2 D7 D :r 3w.y iir 2 t7 51Ay1eE2 D7 8 e 2 67 GydeE2]7 G

Source: 6rosy1r

4. **The use of central venous catheters in paediatric oncology--a cautionary tale.**

Citation:]Au..yEn CroyAes kuwi1es2 qrR c44 2 dusg 0F2 1ug c2 :g cc)cN2 09)400 qrRiweix c44

Author(s): GwEErss2 D7 tieya2 W7 6eA-y1sex2 3 T7 5or12

Source: 6rosy1r

5. **A new approach to management of intravenous infiltration in pediatric patients: pathophysiology, classification, and treatment.**

Citation: uwi1es ul y1lwEyu1 1wiEy1a b .nr ullyAyes :wRsyAe.yu1 ul .nr W1lwEyu1 [wiErE]uAyr.x2 m cc2 dusg 0N2 1ug N2 :g mNm)mN42 cF04) 99 m cc ws)Twa

Author(s): TCkeo2 WRienyC7 6wi:nx2 hiedyE7 [xse1ori),uwEnusori2 Dy1oe7 Ge1l.2 TCe1oe

Source: 6rosy1r

Full Text: TdeyseRsr liuGSear y1fEgmtRh Ei dtigsaEt kgmsaty

6. **Saline irrigation for the management of skin extravasation injury in neonates.**

Citation: hnr tuAnie1r oe.eReEr ul ExE.rCe.yA irdyr(E2 e1 m cm2 1ug m2 :g t8 vN Ng2 cN94)N4 m cm

Author(s): 3u:ese-iyEn1e12 S [7 3urs2 [7 e1rikrr2]wkux

Source: 6rosy1r

Full Text: TdeyseRsr liuGFEwt bah.o Rtr nEtsy1CElwmRt. :aAmRmo( )w.

7. **Extravasation injuries.**

Citation: iy.yEn kuwi1es ul :seE.yA Ewiarix2 6ei c4402 dusg N92 1ug m2 :g 4c)492)cmm9 6ei c440

Author(s): 3ews.2 8 h

Source: 6rosy1r

Full Text: TdeyseRsr liuGSear y1chRsual Rtr 1.lEtsumgluae. ngmy.mo

For peer review only

8. [Central venous catheters in newborn infants. Work Group Parenteral Nutrition of the Perinatology Section of the Dutch Pediatric Society].

**Citation:**	hykoEAniyl. duui -y1oriar1rrE-w1or2 w1 c4402 dusg 9c2 1ug 02 :g 9)vm2 0 9) NNm c440
**Author(s):**	de1 Dy1ar12 G T7 DyrC2 87 iroyr.2 h 3
**Source:**	6rosy1r

9. Extravasation of parenteral alimentation fluid into the renal pelvis--a complication of central venous catheter in a neonate.

**Citation:**	uwi1es ul :riy1e.usuax b ullyAyes kuwi1es ul .nr tesylui1ye Sriy1e.es TEEuAye.yu12 A. m dusg mc2 1ug 2 :g N9F)N992 N0)v0N9 m c A.)[ud
**Author(s):**	[eoiuu2 T 67 es)]u(eysrC2 T 6
**Source:**	6rosy1r
**Full Text:**	TdeyseRsr liuCkRugm. cgAhaswaty FmyEg1lfEgmtRh Ei c.matRuEhEyo<br>TdeyseRsr liuCkRugm. cgAhaswaty FmyEg1lfEgmtRh Ei c.matRuEhEyo<br>TdeyseRsr liuCcmExg.suy1fEgmtRh Ei c.matRuEhEyo

10. Percutaneous central line extravasation masquerading as an abscess.

**Citation:**	W1oye1 :roye.iyAE2 T:i m cN2 dusg Fc2 1ug N2 :g 0 4)0c 2 4 N) FF4 T:iys m cN
**Author(s):**	3udy1o2 y1w7 hr.r2 Sie-eEn Wa1eAr7 hnuCeE2 [yie1ke1
**Source:**	6rosy1r
**Full Text:**	TdeyseRsr liuGnTmaty.m :at2 fEgmtRhdtraRt c.raRumals

For peer review only

Supplementary 2
Study Attrition Process

Level 1 - Is a clinical study of paediatric extravasation injury?

If yes then proceed to level 2

Level 2 - Does it report on one or more of the following outcomes?

- Short-term outcomes:
  - Time to complete tissue healing
  - Infection
  - Hypothermia
  - Anaphylactic reactions
  - Fluid and electrolyte disturbance
  - Any other adverse effects reported in the studies;
- Long-term outcomes (preferably at least three months from the injury):
  - Severity of the scar including contractures, functional impairment, disfigurement and need for further surgical procedures.

Level 3 – is it an original clinical study or systematic review of clinical studies?

If yes then proceed to level 4

Level 4 - In which of the following groups of the study types does it belong?

Group 1

- RCT
- Quasi-randomised study (e.g. alternation rather than randomisation used)
- Systematic reviews of RCTs or quasi-randomised studies

Group 2

- Cross-sectional study
- Case-series
- Systematic reviews of cross-sectional studies or case-series

If yes then retrieve full article for consideration for inclusion

For peer review only

Supplementary 3
Review-Specific Proforma

Author	Patients (n)	Age (mean)	Gender		Comorbidities				Cannula location			Cannula type	
			M	F	Premature	Sepsis	Malignancy	Other	Upper limb	Lower limb	Scalp	Periph eral	Central

Author	Vesicants							Medical Management	Surgical Management	Control (Y/N)
	TPN	≥5% Dextrose	Other IV Fluids	Calcium-containing fluids	Antibiotics	Contrast	Other			

Author	Surgical injuries								Wound outcome	Functional outcome
	Swelling/ Erythema	Partial thickness skin injury	Full thickness skin injury	Tendon injury	Nerve injury	Vascular injury	Compartment Syndrome	Amputation		

For peer review only

PRISMA 2009 Checklist – Surgical Intervention for Paediatric Extravasation

Section/topic	#	Checklist item	Reported on page #
**TITLE**			
Title	1	Identify the report as a systematic review, meta-analysis, or both.	1
**ABSTRACT**			
Structured summary	2	Provide a structured summary including, as applicable: background; objectives; data sources; study eligibility criteria, participants, and interventions; study appraisal and synthesis methods; results; limitations; conclusions and implications of key findings; systematic review registration number.	2
**INTRODUCTION**			
Rationale	3	Describe the rationale for the review in the context of what is already known.	4
Objectives	4	Provide an explicit statement of questions being addressed with reference to participants, interventions, comparisons, outcomes, and study design (PICOS).	5
**METHODS**			
Protocol and registration	5	Indicate if a review protocol exists, if and where it can be accessed (e.g., Web address), and, if available, provide registration information including registration number.	6
Eligibility criteria	6	Specify study characteristics (e.g., PICOS, length of follow-up) and report characteristics (e.g., years considered, language, publication status) used as criteria for eligibility, giving rationale.	6
Information sources	7	Describe all information sources (e.g., databases with dates of coverage, contact with study authors to identify additional studies) in the search and date last searched.	6,7,8
Search	8	Present full electronic search strategy for at least one database, including any limits used, such that it could be repeated.	Supplementary-1
Study selection	9	State the process for selecting studies (i.e., screening, eligibility, included in systematic review, and, if applicable, included in the meta-analysis).	7
Data collection process	10	Describe method of data extraction from reports (e.g., piloted forms, independently, in duplicate) and any processes for obtaining and confirming data from investigators.	Supplementary-2
Data items	11	List and define all variables for which data were sought (e.g., PICOS, funding sources) and any assumptions and simplifications made.	Supplementary-2
Risk of bias in individual studies	12	Describe methods used for assessing risk of bias of individual studies (including specification of whether this was done at the study or outcome level), and how this information is to be used in any data synthesis.	7
Summary measures	13	State the principal summary measures (e.g., risk ratio, difference in means).	N/A
Synthesis of results	14	Describe the methods of handling data and combining results of studies, if done, including measures of consistency (e.g., I^2) for each meta-analysis.	N/A

For peer review only

PRISMA 2009 Checklist – Surgical Intervention for Paediatric Extravasation

Section/topic	#	Checklist item	Reported on page #
Risk of bias across studies	15	Specify any assessment of risk of bias that may affect the cumulative evidence (e.g., publication bias, selective reporting within studies).	N/A
Additional analyses	16	Describe methods of additional analyses (e.g., sensitivity or subgroup analyses, meta-regression), if done, indicating which were pre-specified.	N/A
**RESULTS**			
Study selection	17	Give numbers of studies screened, assessed for eligibility, and included in the review, with reasons for exclusions at each stage, ideally with a flow diagram.	8
Study characteristics	18	For each study, present characteristics for which data were extracted (e.g., study size, PICOS, follow-up period) and provide the citations.	Table 1 + Table 2
Risk of bias within studies	19	Present data on risk of bias of each study and, if available, any outcome level assessment (see item 12).	Table 5
Results of individual studies	20	For all outcomes considered (benefits or harms), present, for each study: (a) simple summary data for each intervention group (b) effect estimates and confidence intervals, ideally with a forest plot.	Table 4
Synthesis of results	21	Present results of each meta-analysis done, including confidence intervals and measures of consistency.	N/A
Risk of bias across studies	22	Present results of any assessment of risk of bias across studies (see Item 15).	N/A
Additional analysis	23	Give results of additional analyses, if done (e.g., sensitivity or subgroup analyses, meta-regression [see Item 16]).	N/A
**DISCUSSION**			
Summary of evidence	24	Summarize the main findings including the strength of evidence for each main outcome; consider their relevance to key groups (e.g., healthcare providers, users, and policy makers).	14-17
Limitations	25	Discuss limitations at study and outcome level (e.g., risk of bias), and at review-level (e.g., incomplete retrieval of identified research, reporting bias).	13
Conclusions	26	Provide a general interpretation of the results in the context of other evidence, and implications for future research.	19
**FUNDING**			
Funding	27	Describe sources of funding for the systematic review and other support (e.g., supply of data); role of funders for the systematic review.	1

From: Moher D, Liberati A, Tetzlaff J, Altman DG, The PRISMA Group (2009). Preferred Reporting Items for Systematic Reviews and Meta-Analyses: The PRISMA Statement. PLoS Med 6(6): e1000097. doi:10.1371/journal.pmed1000097

For more information, visit: **www.prisma-statement.org**.

For peer review only - http://bmjopen.bmj.com/site/about/guidelines.xhtml

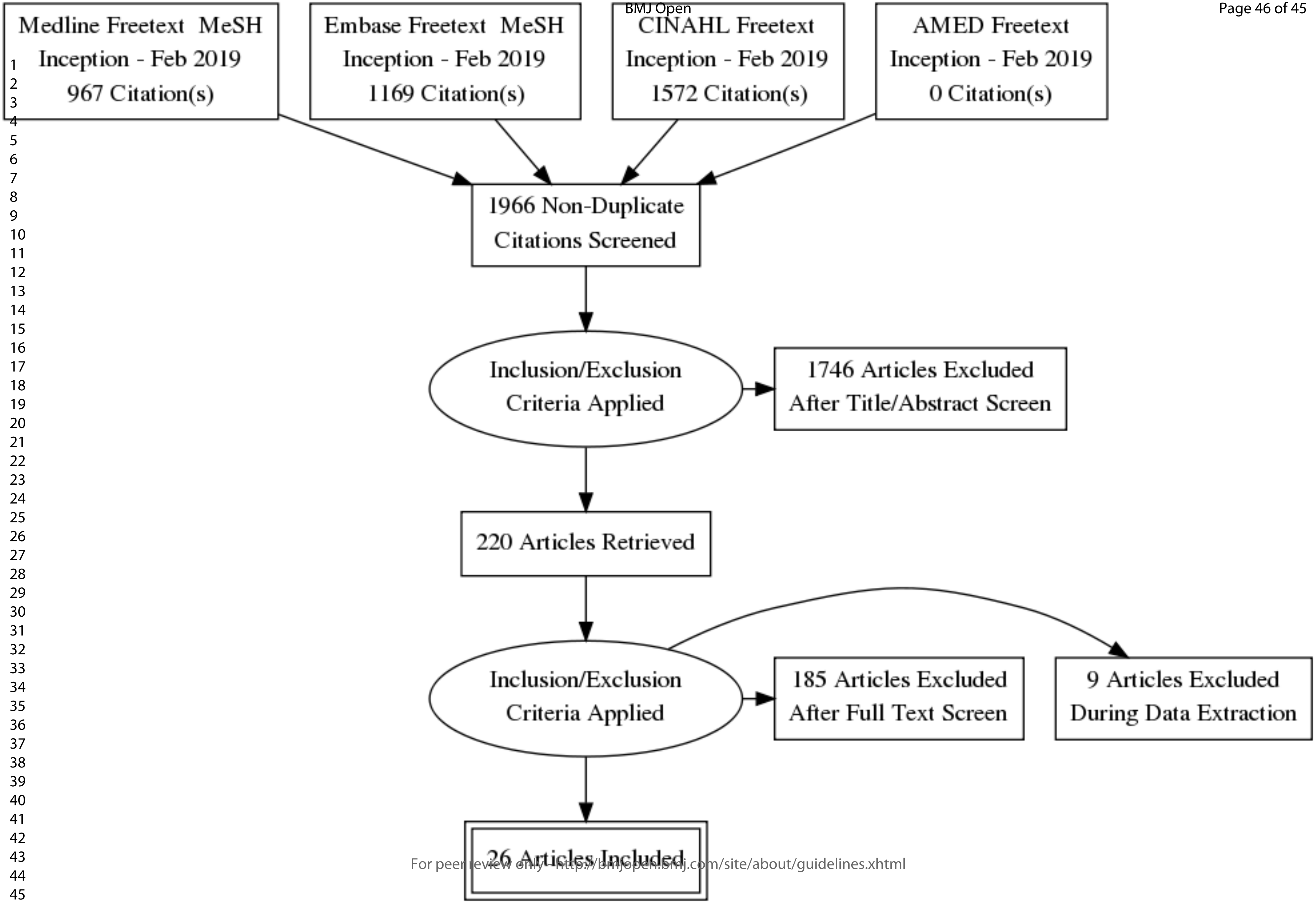
Medline Freetext MeSH
Inception - Feb 2019
967 Citation(s)
Embase Freetext MeSH
Inception - Feb 2019
1169 Citation(s)
CINAHL Freetext
Inception - Feb 2019
1572 Citation(s)
AMED Freetext
Inception - Feb 2019
0 Citation(s)
1966 Non-Duplicate Citations Screened
Inclusion/Exclusion Criteria Applied
1746 Articles Excluded After Title/Abstract Screen
220 Articles Retrieved
Inclusion/Exclusion Criteria Applied
185 Articles Excluded After Full Text Screen
9 Articles Excluded During Data Extraction
26 Articles Included

BMJ Open

Surgical Intervention for Paediatric Infusion-Related Extravasation Injury: A Systematic Review

Journal:	*BMJ Open*
Manuscript ID	bmjopen-2019-034950.R1
Article Type:	Original research
Date Submitted by the Author:	11-Mar-2020
Complete List of Authors:	Little, Max; Whittington Hospital NHS Trust, General Surgery<br>Dupré, Sophie; University of Oxford Nuffield Department of Orthopaedics Rheumatology and Musculoskeletal Sciences<br>Wormald, Justin; University of Oxford Nuffield Department of Orthopaedics Rheumatology and Musculoskeletal Sciences; Stoke Mandeville Hospital, Plastic & Reconstructive Surgery<br>Gardiner, Matthew; University of Oxford Nuffield Department of Orthopaedics Rheumatology and Musculoskeletal Sciences; Wexham Park Hospital, Plastic & Reconstructive Surgery<br>Gale, Chris; Imperial College London, Neonatal Medicine, School of Public Health<br>Jain, Abhilash; University of Oxford Nuffield Department of Orthopaedics Rheumatology and Musculoskeletal Sciences, Plastic Surgery; Imperial College Healthcare NHS Trust, Plastic & Reconstructive Surgery
**Primary Subject Heading**:	Surgery
Secondary Subject Heading:	Paediatrics, Evidence based practice, Intensive care
Keywords:	Paediatric plastic & reconstructive surgery < PAEDIATRIC SURGERY, PLASTIC & RECONSTRUCTIVE SURGERY, Paediatric intensive & critical care < PAEDIATRICS, Neonatal intensive & critical care < INTENSIVE & CRITICAL CARE

SCHOLARONE™
Manuscripts

I, the Submitting Author has the right to grant and does grant on behalf of all authors of the Work (as defined in the below author licence), an exclusive licence and/or a non-exclusive licence for contributions from authors who are: i) UK Crown employees; ii) where BMJ has agreed a CC-BY licence shall apply, and/or iii) in accordance with the terms applicable for US Federal Government officers or employees acting as part of their official duties; on a worldwide, perpetual, irrevocable, royalty-free basis to BMJ Publishing Group Ltd ("BMJ") its licensees and where the relevant Journal is co-owned by BMJ to the co-owners of the Journal, to publish the Work in this journal and any other BMJ products and to exploit all rights, as set out in our licence.

The Submitting Author accepts and understands that any supply made under these terms is made by BMJ to the Submitting Author unless you are acting as an employee on behalf of your employer or a postgraduate student of an affiliated institution which is paying any applicable article publishing charge ("APC") for Open Access articles. Where the Submitting Author wishes to make the Work available on an Open Access basis (and intends to pay the relevant APC), the terms of reuse of such Open Access shall be governed by a Creative Commons licence – details of these licences and which Creative Commons licence will apply to this Work are set out in our licence referred to above.

Other than as permitted in any relevant BMJ Author's Self Archiving Policies, I confirm this Work has not been accepted for publication elsewhere, is not being considered for publication elsewhere and does not duplicate material already published. I confirm all authors consent to publication of this Work and authorise the granting of this licence.

For peer review only

Surgical Intervention for Paediatric Infusion-Related Extravasation Injury: A Systematic Review

Little, Max[1]; Dupré, Sophie[2]; Wormald, Justin[3]; Gardiner, Matthew[4]; Gale, Chris[5]; Jain, Abhilash[2,6]

1. Department of Surgery, The Whittington Hospital, Magdala Avenue, London, UK, N19 5NF
2. Nuffield Department of Orthopaedics, Rheumatology and Musculoskeletal Sciences, University of Oxford, Oxford, UK, OX1 2DJ
3. Department of Plastic Surgery, Stoke Mandeville Hospital, Aylesbury, Buckinghamshire, UK, HP21 8AL
4. Department of Plastic Surgery, Wexham Park Hospital, Wexham St, Slough, UK, SL2 4HL
5. Neonatal Medicine, Department of Medicine, Imperial College London, Chelsea and Westminster Campus, London, UK, SW10 9NH
6. Department of Plastic and Reconstructive Surgery, Imperial College Healthcare NHS Trust, St Mary's Hospital, Praed St, London, UK, W2 1NY

Mr Max Little MBBS MRCS
Core Surgical Trainee, The Whittington Hospital NHS Trust, London, UK

Dr Sophie Dupré MBBS BSc (Hons)
Academic Foundation Doctor, University of Oxford, UK

Mr Justin C R Wormald MBBS MRes MRCS
NIHR Academic Clinical Fellow and Specialist Registrar in Plastic and Reconstructive Surgery, University of Oxford, UK

Mr Matthew Gardiner MA PhD FRCS (Plast)
Hand Fellow, NIHR Senior Clinical Lecturer and Specialist Registrar in Plastic and Reconstructive Surgery, University of Oxford, UK

Dr Chris Gale MBBS MSc PhD MRCPCH *
Clinical Senior Lecturer in Neonatal Medicine and Consultant Neonatologist, Imperial College London, UK

Professor Abhilash Jain MBBS MRCS MSc PhD FRCS (Plast) *
Associate Professor of Plastic and Reconstructive Surgery, University of Oxford and Imperial College NHS Trust, London, UK
*Co-Senior Authors

Corresponding author:
Dr Chris Gale
christopher.gale@imperial.ac.uk
+44 (0)20 3315 3519
Academic Neonatal Medicine, H4.4
Chelsea and Westminster Hospital
Chelsea and Westminster Campus

Running Head: Paediatric extravasation injury

Keywords: Extravasation; Infiltration; Paediatric; Necrosis; Gault

Word Count: 3,980 (including abstract and summary)

For peer review only

Abstract

Objectives

This systematic review aims to assess the quality of literature supporting surgical interventions for paediatric extravasation injury and to determine whether there is sufficient evidence to support invasive techniques in children.

Methods

We performed a systematic review by searching Ovid MEDLINE and EMBASE as well as AMED, CINAHL, Cochrane Central Register of Controlled Trials (CENTRAL), Cochrane Database of Systematic Reviews and clinicaltrials.gov from inception to February 2019. Studies other than case reports were eligible for inclusion if the population was younger than 18 years old, there was a surgical intervention aimed at treating extravasation injury and they reported on outcomes. Study quality was graded according to the National Institutes of Health (NIH) study quality assessment tools.

Results

26 studies involving 728 children were included – one before-and-after study and 25 case series. Extravasation injuries were mainly confined to skin and subcutaneous tissues but severe complications were also encountered, including amputation (one toe and one below elbow). Of the surgical treatments described, the technique of multiple puncture wounds and instillation of saline and/or hyaluronidase was the most commonly used. However, there were no studies in which its effectiveness was tested against another treatment or a control and details of functional and aesthetic outcomes were generally lacking.

Conclusion

Surgical management is commonly reported in the literature in cases where there is significant soft tissue injury but as there are no comparative studies, it is unclear whether this is optimal. Further observational and experimental research evaluating extravasation injuries, including a centralized extravasation register using a universal grading scheme and core outcome set with adequate follow-up, are required to provide evidence to guide clinician decision-making.

For peer review only

Strengths and Limitations

- A systematic review was performed in accordance with the Preferred Reporting Items for Systematic Reviews and Meta-Analyses (PRISMA) statement and using methodology outlined in the Cochrane Handbook for Systematic Reviews of Interventions. It was registered on PROSPERO.
- Two authors used a bespoke inclusion/exclusion form to independently assess study eligibility
- Studies were eligible for inclusion if the population was younger than 18 years old, if there was a surgical intervention aimed at treating extravasation injury in any setting and if they reported on short- or long-term outcomes
- Two researchers also independently assessed the included studies' risk of methodological bias using the National Institutes of Health (NIH) study quality assessment tools
- 18 years old may represent a relatively arbitrary cut-off age to differentiate between 'paediatric' and 'adult' in terms of extravasation injury

For peer review only

For peer review only - http://bmjopen.bmj.com/site/about/guidelines.xhtml

Introduction

Extravasation is the inadvertent leakage of a vesicant solution from its intended vascular pathway, commonly a peripheral or central vein, into the surrounding tissue with the potential to harm a patient. A vesicant is any medicine or fluid with the potential to cause blisters, severe tissue injury or necrosis if there is leakage. [1] If left untreated, extravasations may result in delayed healing, scarring and functional morbidity, including amputation in severe cases. Children requiring intravenous (IV) therapy often have multiple risk factors for extravasation injuries, and neonates are at even greater risk of more serious injury due to poor venous integrity, capillary leakage, and expandable subcutaneous tissue that accommodates relatively large volumes of fluid. [2] The reported incidence in adult and paediatric inpatients ranges between 0.1% - 6.5% across studies from the UK, USA and Canada, but inconsistent reporting and documentation means that the true figure is likely to be higher. [2–4]

Prevention of extravasation is the foremost priority. However, when injury does occur, multiple treatments have been described. The “Gault technique” [5] utilizes multiple small incisions made around the injury site. These are subsequently used to instill saline and/or hyaluronidase to wash out the extravasation fluid. This method remains a commonly described technique for the treatment of more severe paediatric extravasations. However, there is a lack of high quality comparative studies in which treatments are evaluated against each other and/or controls. It is therefore not known which of these treatments are effective, harmful or at the very least, necessary; a conclusion supported by a recent scoping review of interventions. [6] That review highlighted the fact that the lack of consensus extended to published guidelines. Their survey also showed wide variation in the frequency of use of the saline washout techniques in neonatal units across the National Health Service (NHS) in the UK. This systematic review aims to evaluate the quality of the literature and to summarize the published outcome data of the surgical management of paediatric extravasation injuries.

For peer review only - http://bmjopen.bmj.com/site/about/guidelines.xhtml

Methods

Our aim was to assess the quality of literature supporting surgical interventions for paediatric extravasation injury and to determine whether there is sufficient evidence to support invasive techniques in children. We performed a systematic review of original clinical studies or systematic reviews (excluding case reports) of paediatric extravasation injury, reported in accordance with the PRISMA [7] statement and using methodology outlined in the Cochrane Handbook for Systematic Reviews of Interventions [8] where applicable. The protocol was developed prospectively, locally peer-reviewed and registered on the PROSPERO database (CRD42016045647). Variability in baseline data, intervention data and outcome data precluded formal meta-analysis. Results are therefore presented as a narrative comparison of different invasive techniques with summary statistics.

Search methods

Studies were identified through a systematic literature search in Ovid MEDLINE and Ovid EMBASE as well as AMED, CINAHL, Cochrane Central Register of Controlled Trials (CENTRAL), Cochrane Database of Systematic Reviews and clinicaltrials.gov from inception to February 2019. To identify grey literature, Web of Science was searched using bespoke searches. Internet-wide searches using Google were employed to identify clinical guidelines describing the management of extravasation in the UK. All of these strategies comprised modifications of a common set of terms to identify the relevant clinical area. Key search terms in this common set included the Medical Subject Headings (MeSH) “extravasation of diagnostic and therapeutic materials", “infant”, “child”, and “preschool”. These MeSH terms were combined with appropriate Boolean operators [Supplementary 1]. The index search terms were then combined with free text terms to identify appropriate clinical studies. No language restrictions were imposed. The search results were merged and, after screening, duplicate citations were discarded along with studies unrelated to the research objective.

For peer review only

Criteria for selecting studies

Study selection criteria were determined in advance during the protocol stage. Two authors used a bespoke inclusion/exclusion form to independently assess eligibility of the title and abstracts of the search results. [Supplementary 2] Randomized Control Trials (RCTs), quasi-experimental and cross-sectional studies and case-series were eligible for inclusion if the population was younger than 18 years old, if there was a surgical intervention aimed at treating extravasation injury in any setting and if they reported on short- or long-term outcomes. Hyaluronidase injections were considered to be a surgical intervention for the purposes of this review firstly, because they are an invasive procedure and secondly, to allow clinically useful comparison to the combination of Gault's technique with hyaluronidase. Any disparities regarding inclusion of articles were discussed between the authors and a joint decision was made based on the inclusion criteria.

Data analysis

Data extraction was performed by two authors independently and is displayed in Tables 1-5. Descriptive analysis for each group of studies was performed, including the number of children with skin necrosis at presentation as well as the difference in outcomes between those who received an intervention and those who received no intervention.

Study quality assessment

Two researchers also independently assessed the quality of the included studies using the National Institutes of Health (NIH) study quality assessment tools [9] for "case series" and for "before-after studies with no control group" – the two types of included studies. Discrepancies were resolved by discussions with the other authors.

Patient and Public Involvement

No patients involved

For peer review only - http://bmjopen.bmj.com/site/about/guidelines.xhtml

Results

Search strategy

Our search strategy identified a total of 1,966 research articles of which 220 were potentially relevant to the research question. Of the 220 papers 185 did not meet the inclusion criteria. Thirty-five papers were deemed eligible for inclusion after studies with conservative management or no interventions were excluded. The requisite data were immediately available in 26 study reports [4,10–34] and the authors of the remaining nine studies [5,35–42] were contacted twice with additional requests, mainly for exclusively paediatric extravasation data, and given at least two months to reply. Unfortunately, none of the authors were able to provide these data and those studies were excluded. The data were extracted from the remaining 26 studies [Supplementary PRISMA flow-diagram] using a pre-specified (review-specific) proforma [Supplementary 3] by two researchers, with discrepancies resolved by a third.

Study Characteristics

The included studies were published between 1975 and 2018. The mean sample size was 32 with a range of 4-147 participants. One study was a before-and-after comparative study, [24] two were prospective case series, [11,16] and the remaining 23 were retrospective case series. The follow-up periods were not reported in 14 included studies. In the other studies, the follow-up periods ranged from three days to 15 months [Table 1].

For peer review only - http://bmjopen.bmj.com/site/about/guidelines.xhtml

Table 1 – Included studies

Rf	Author	Year	Country	Title	n	Methods	Period	Mean follow-up
4	Murphy	2017	Australia	Extravasation injury in a paediatric population	43	Retrospective Series	-	-
10	Falcone	1989	USA	Nonoperative management of full-thickness intravenous extravasation injuries in premature neonates using enzymatic debridement	15	Retrospective Series	-	6 months
19	Ghanem	2015	UK	Childhood extravasation injuries: improved outcome following the introduction of hospital-wide guidelines	48	Prospective Series	1 year	-
20	Kostogloudis	2015	Greece	Severe Extravasation Injuries in Neonates: A Report of 34 Cases	34	Retrospective Series	2 years	15 months
21	Sung	2016	Korea	Nonoperative Management of Extravasation Injuries Associated With Neonatal Parenteral Nutrition Using Multiple Punctures and a Hydrocolloid Dressing.	12	Retrospective Series	4 years	10 months
22	Odom	2018	USA	Peripheral Infiltration and Extravasation Injury Methodology: A Retrospective Study.	147	Retrospective Series	-	-
23	Compaña	2017	Spain	Lesions Associated With Calcium Gluconate Extravasation	4	Retrospective Series	-	2 months
24	Harris	2001	UK	Limiting the damage of iatrogenic extravasation injury in neonates	56	Prospective Series	3 years	-
25	Andrés	2006	Spain	Treatment protocol for extravasation lesions	15	Retrospective Series	6 years	-
26<br>27	Linder	1983<br>1985	USA	Management of extensive doxorubicin hydrochloride extravasation injuries<br>Prevention of extravasation injuries secondary to doxorubicin	18	Retrospective Series	-	3 months
28	Upton	1979	USA	Major intravenous extravasation injuries	7	Retrospective Series	10 years	-
11	Casanova	2001	France	Emergency treatment of accidental infusion leakage in the newborn: Report of 14 cases	14	Retrospective Series	-	-
29	von Heimburg	1998	Germany	Early and late treatment of iatrogenic injection damage	5	Retrospective Series	-	-
30	Weiss	1975	Israel	Localized necrosis of scalp in neonates due to calcium gluconate infusions: a cautionary note	4	Retrospective Series	-	-
31	Hanrahan	2013	USA	Hyaluronidase for treatment of intravenous extravasations: implementation of an evidence-based guideline in a pediatric population	113	Before-and-after study	4 years	-
32	Boyar	2018	USA	Efficacy of Dehydrated Human Amniotic Membrane Allograft for the Treatment of Severe Extravasation Injuries in Preterm Neonates	4	Retrospective Series	-	1-2 months
33	Yan	2017	China	Incidence, risk factors and treatment outcomes of drug extravasation in pediatric patients in China	18	Retrospective Series	6 months	1– 5 months
34	Myers	2018	USA	Managing intravenous infiltration injuries in the neonatal intensive care unit	28	Retrospective Series	7 years	-
12	Cochran	2002	USA	Treatment of iodinated contrast material extravasation with hyaluronidase	8	Retrospective Series	7 years	-
13	Cho	2007	Korea	Successful combined treatment with total parenteral nutrition fluid extravasation injuries in preterm infants	5	Retrospective Series	4 months	-
14	Onesti	2012	Italy	The use of hyalomatrix PA in the treatment of extravasation affecting premature neonates	26	Retrospective Series	6 years	14 months
15	Firat	2013	Turkey	Management of extravasation injuries: a retrospective study	13	Retrospective Series	2 years	-
16	Ching	2014	UK	Paediatric extravasation injuries: a review of 69 consecutive patients	69	Retrospective Series	1 year	3 days
17	Sivrioglu	2014	Turkey	Versajet hydrosurgery system in the debridement of skin necrosis after Ca gluconate extravasation: report of 9 infantile cases	9	Cohort Study	-	1 year
18	Yan	2014	China	Treatment of cutaneous injuries of neonates induced by drug extravasation with hyaluronidase and hirudoid	13	Retrospective Series	2 years	3 months

For peer review only

For peer review only - http://bmjopen.bmj.com/site/about/guidelines.xhtml

Patient Characteristics

Across the 26 included studies, there were 728 children. The median age of the 68 children for whom the individual data were available was 0.66 months (interquartile range (IQR) = 5.05). The mean age of 14 months (range: neonatal to 17 years old) was calculated using the data for 322 children across 16 included studies. There was a small difference in sex, with 187 males (57%) and 142 females (43%) (data on sex was lacking for 399 children). 19 of the 26 included studies recorded the sites of the implicated cannula for 553 children. The most common site was the upper limb (n=343; 62%), followed by the lower limb (n=177; 32%) and then the scalp (n=21; 4%). Ten studies specifically stated whether the cannula was a peripheral (n=314; 98%) or central line (n=7; 2%). [Table 2]

For peer review only

Table 2 – Demographics

Ref	Author	n	Mean age (months)	Male	Female	N upper limb	N lower limb	N Scalp	N other	Peripheral Cannula	Central Cannula
**Gault Technique Studies**											
4	Murphy	43	-	-	-	32	9	-	-	-	-
11	Ghanem	48	38.4	-	-	48	25	4	5	73	7
12	Kostogloudis	34	0.6	-	-	6	28	-	-	34	-
17	Andrés	15	36	-	-	14	-	1	-	15	-
21	Casanova	14	1	-	-	4	9	1	-	-	-
32	Ching	69	0.7	32	37	45	17	-	7	-	-
24	Harris	56	-	-	-	-	-	-	-	-	-
	**Total**	**279**	**15.3 (mean)**	**32**	**37**	**149**	**88**	**6**	**12**	**122**	**7**
**Debridement + further surgery**											
10	Falcone	15	0.6	7	8	12	2	1	-	-	-
13	Sung	12	-	6	6	7	5	-	-	-	-
15	Compaña	4	2.6	3	1	2	2	-	-	4	-
17	Andrés (2)	15	36	-	-	14	-	1	-	15	-
20	Upton	7	67.2	-	-	-	-	-	-	-	-
23	Weiss	4	-	-	-	-	-	4	-	4	-
25	Boyar	4	1.1	3	1	3	1	-	-	4	-
29	Cho	5	0.6	-	-	4	-	-	-	6	-
30	Onesti	26	0.6	17	9	14	10	2	-	-	-
31	Firat	13	50	3	10	6	4	3	-	-	-
33	Sivrioglu	9	0.9	-	-	5	3	1	-	9	-
26 27	Linder	18	-	-	-	-	-	-	-	-	-
29	Von Heimburg	5	-	-	-	-	-	-	-	-	-
	**Total**	**137**	**17.7 (mean)**	**46**	**28**	**67**	**27**	**12**	**0**	**42**	**0**
**Hyaluronidase injections**											
14	Odom	147	-	87	60	106	40	2	-	147	-
26	Yan (2017)	18	39.7	10	8	12	6	-	-	18	-
27	Myers	28	1.3	-	-	14	13	1	-	-	-
28	Cochran	8	-	4	4	-	-	-	-	-	-
34	Yan (2014)	13	0.9	8	5	9	3	1	-	-	-
31	Hanrahan	113	-	-	-	-	-	-	-	-	-
	**Total**	**327**	**14.0 (mean)**	**109**	**77**	**141**	**62**	**4**	**0**	**165**	**0**
	**Overall Totals**	**728**	**15.13 (mean)**	**187**	**142**	**343**	**177**	**21**	**12**	**314**	**7**

For peer review only

For peer review only - http://bmjopen.bmj.com/site/about/guidelines.xhtml

Of the 16 studies that provided data on comorbidities for 167 children, prematurity was the most common comorbidity in 11 (n=121; 72%). Malignancy (n=25; 15%) was the main comorbidity in three studies.

Of the 20 studies that reported on them, the most commonly extravasated substances affecting 363 children were [Table 3]:

1) Total Parenteral Nutrition (TPN) – 122 children (34%); most common in eight studies.
2) Intravenous maintenance fluids other than those specifically listed – 70 children (19%); most common in three studies. Unfortunately the data was not available for the outcomes of different types of maintenance fluids so they have been grouped together.
3) Calcium-containing products – 32 children (9%); most common in four studies.
4) Dextrose of concentration ≥5% - 28 children (8%); most common in one study.
5) Intravenous antimicrobials – 20 children (6%); most common in no studies. Of those 20 extravasations, five were due to Flucloxacillin – with one injury taking 60 days – to heal and five were due to unspecified cephalosporin antibiotics, three of which required skin grafting. One case of severe contractures and extensor loss on the dorsum of a child's hand was due to an unspecified tetracycline antibiotic. There were no individual outcomes recorded for the seven extravasations of Aciclovir, the single extravasations of Gentamicin, Ganciclovir or the remaining extravasations involving Flucloxacillin and cephalosporin antibiotics.

For peer review only

Table 3 – Comorbidities and extravasated materials in those papers that recorded them

		Comorbidities			Vesicant Type								
Ref	Author	Prematurity	Sepsis	Malignancy	TPN	≥5% Dextrose	Other IV Fluids	Calcium-containing	Antimicrobials	Chemo	Contrast	Dopamine	Other
**Gault Technique Studies**													
4	Murphy	-	-	-	10	11	6	2	6	-	2	-	5
19	Ghanem	14	-	-	22	10	6	-	8	-	-	1	1
20	Kostogloudis	34	-	-	28	4	-	-	2	-	-	-	-
25	Andrés	-	1	5	7	-	-	4	-	4	-	-	-
11	Casanova	-	-	-	-	-	-	2	-	-	-	9	3
16	Ching	"Majority"	-	-	16	-	22	-	-	-	-	-	31
	**Total**	**48**	**1**	**5**	**83**	**25**	**34**	**8**	**16**	**4**	**2**	**10**	**40**
**Debridement + further surgery**													
10	Falcone	15	-	-	9	1	3	1	-	-	-	-	2
21	Sung	9	-	-	12	-	-	-	-	-	-	-	-
23	Compaña	2	-	-	-	-	-	4	-	-	-	-	-
25	Andrés (2)	-	1	5	7	-	-	4	-	4	-	-	-
26 27	Linder	-	-	18	-	-	-	-	-	18	-	-	-
28	Upton	-	-	1	-	1	2	1	1	2	-	-	-
30	Weiss	4	-	-	-	-	-	4	-	-	-	-	-
32	Boyar	4	-	-	4	-	-	-	-	-	-	-	-
13	Cho	5	-	-	4	-	-	-	-	-	-	-	1
14	Onesti	26	-	-	-	-	26	-	-	-	-	-	-
15	Firat	-	1	1	-	1	4	3	3	1	-	-	1
17	Sivrioglu	3	-	-	-	-	-	9	-	-	-	-	-
	**Total**	**68**	**2**	**25**	**36**	**3**	**35**	**26**	**4**	**25**	**0**	**0**	**4**
**Hyaluronidase injections**													
33	Yan (2017)	-	-	-	1	-	-	1	-	-	-	-	-
12	Cochran	-	-	-	-	-	-	-	-	-	8	-	-
18	Yan (2014)	5	-	-	9	-	1	1	-	-	-	-	2
	**Total**	**5**	**0**	**0**	**10**	**0**	**1**	**2**	**0**	**0**	**8**	**0**	**2**
	**Overall Totals**	**121**	**2**	**25**	**122**	**28**	**70**	**32**	**20**	**25**	**10**	**10**	**46**

For peer review only

Interventions

Uncertainty remains over whether there is a difference in the outcomes of children who are treated conservatively or surgically and the case series that exist on conservative management cover a number of treatments for a range of injuries from swelling to full-thickness defects. However, a recent scoping review found that the outcomes used and results detailed were generally limited, [6] making it difficult for clinicians to rely upon those studies in deciding who to treat conservatively. This is why we have focused solely on studies describing invasive interventions: the Gault technique, debridement and further surgery, and hyaluronidase injections. We have still reported on the outcomes of children within each group of studies who received no treatment in order to provide a clinically useful comparison to each intervention.

<ins>The Gault technique</ins>

279 children with an average age of 15.3 months were involved in the seven studies looking at the Gault technique. No patients had skin necrosis at presentation.

107 were treated using the Gault technique with saline, of which nine (8%) developed full thickness skin injury / skin necrosis and one (1%) developed compartment syndrome requiring fasciotomies. Of those with necrosis, six required no surgical intervention and three were debrided and treated with artificial dermis.

76 were treated using the Gault technique with hyaluronidase, of which three (4%) developed skin necrosis and were all managed conservatively.

One child received only hyaluronidase injections and developed necrosis, which healed spontaneously.

95 children received no treatment, of which ten (11%) developed necrosis (one of whom went on to lose three toes), three (3%) developed an associated infection, one (1%) had an ischaemic toe requiring amputation and one (1%) developed calcinosis cutis. [Table 4]

For peer review only - http://bmjopen.bmj.com/site/about/guidelines.xhtml

Table 4 – Interventions

Ref	Author	Intervention Summary	Outcomes
**Gault Technique Studies**			
4	Murphy	Gault's + saline (11% of patients).	Three patients (7%) suffered injuries, which led to significant tissue necrosis, delayed healing and prolonged morbidity. None of these were washed out due to delayed referral.
20	Kostogloudis	Gault's + saline (100% of patients).	All wounds healed within 25 days. One case of distal foot ischaemia resolved after treatment.
24	Harris	Gault's + saline (100% of patients).	No episodes of skin or soft tissue loss were recorded and no reconstructive surgery was required.
25	Andrés	Gault's + saline (67% of patients) + Debridement + dermal substitute in 53%	Seven of ten treated with Gault's technique avoided necrosis and recovered fully. Three developed necrosis and, with the remaining five, were debrided and received artificial skin and obtained satisfactory outcomes.
11	Casanova	Gault's + hyaluronidase (79% of patients) / + saline (14%) with liposuction	No skin involvement in ten patients; Blistering healed in one; Necrosis resolved in three
19	Ghanem	Gault's + hyaluronidase (46% of patients) with liposuction	Three patients had tissue necrosis (one of whom received wash out). There was satisfactory healing with no requirement for surgical intervention.
16	Ching	Gault's + hyaluronidase (62% of patients).	Of the 62% of patients washed out, none developed complications. Two other significant complications.
**Debridement + further surgery**			
10	Falcone	Topical fibrinolysin/deoxyribonuclease ointment then debridement.	All wounds healed completely with no infections and no functional scar contractions at up to 16 months follow-up. No skin grafts were required.
21	Sung	Multiple punctures using a scalpel blade + hydrocolloid dressing. Then debridement.	All patients showed favourable results without functional deficits or conspicuous scars
23	Compaña	Topical steroids, Burow's Solution and Silver Sulfadiazine. 3 debridements followed by split-thickness skin grafts.	Of four patients, two successfully healed, one was lost to follow-up and one died of other causes.
25	Andrés (2)	Gault's + saline (67% of patients) + debridement in 33% + artificial dermis.	Seven of the ten patients treated with Gault's technique avoided necrosis and recovered fully. Three developed necrosis. The five who were debrided and received artificial skin obtained satisfactory outcomes.
30	Weiss	Wet dressings and repeated economical debridement.	Wounds healed well in 15 to 40 days. Scars were visible but without discolouration
32	Boyar	Enzymatic or autolytic debridement before mechanical debridement and application of dehydrated human amniotic membrane allograft (dHAMA)	Complete closure of significant wounds with minimal soft scars and normal pigmentation
13	Cho	Topical antibiotic + anti-inflammatory herbal mixture. 1 debridement. 1 escharotomy.	One patient had pigmentation at four weeks, one had a small-sized contracture at 50 days, one had small linear scar at two years.
14	Onesti	Topical collagenase, then debridement, and then Hyalomatrix PA (dermal substitute).	18 patients healed fully after 21 days. Four had pathological scars and four had debilitating scar contractures needing secondary surgery.
15	Firat	Topical hirudin and antibiotics, then 3% boric acid, then repetitive debridement.	Seven patients required split-thickness skin grafting and two required fasciocutaneous flaps. All recovered well with scar development in four. Minor functional loss in the hands or feet as a result of scar formation was managed by physiotherapy and pressure garments.
26 27	Linder	Debridement and wound closure: mostly split-thickness skin grafts or delayed primary closure. All patients had at least 2 operations.	The mean time for wound closure was 49 days (range 10–85 days). Three patients died before wound closure. At least one patient needed a split-thickness skin graft. One child developed sympathetic dystrophy syndrome. Some children developed permanent joint stiffness.
28	Upton	Debridement and skin grafts. All patients needed two or more operations.	Two patients experienced contractures, two had extensor loss, one had hair loss, one had loss of motion and one required further reconstruction.
29	von Heimburg	Debridement, allogeneic donor tissue grafts and autologous split-thickness skin grafts.	After 15 days there was full healing in all five infants
17	Sivrioğlu	Versajet hydrosurgery for all patients. 1 sharp debridement.	Minimal scar formation with no hypertrophic scarring in any patient.
**Hyaluronidase injections**			
22	Odom	Injection of hyaluronidase or Phentolamine without incisions.	No patients required surgical intervention for wound healing or had an infection.
31	Hanrahan	Injection of hyaluronidase.	Mean harm scores were similar between the group receiving hyaluronidase and the group not receiving it.
33	Yan (2017)	Injection of hyaluronidase in 33% (1 patient requiring surgical excision of a lesion)	All healed and had "good outcomes"
34	Myers	Injection of hyaluronidase in 50%	Time to healing averaged 16.2 days (range 1-82 days). No patient required surgical intervention.
12	Cochran	Injection of hyaluronidase in 25%	One patient had a prolonged course with swelling and skin peeling of the hand.
18	Yan (2014)	Injection of hyaluronidase and application of hirudoid.	Three patients lost to follow-up. Negligible loss of functional movements. One case of scarring and readmission with calcinosis.

For peer review only - http://bmjopen.bmj.com/site/about/guidelines.xhtml

Debridement and further surgery

There were 137 children with an average age of 17.7 months across the 14 studies in the debridement and further surgery group.

Nine studies (98 children) used management plans comprising debridement and further surgery in combination with conservative measures, details of which are shown in Table 4. [10,13,15,17,23,25,29–31] Of those 98 children, 34 (35%) had established full thickness skin injury / skin necrosis at presentation and 28 (29%) developed necrosis after conservative measures. 84 children (86%) underwent mechanical debridement as part of their treatment. The vast majority of children's wounds healed with minimal scarring and no functional deficit but four (4%) had debilitating scar contractures necessitating further surgery, ten (10%) required skin grafts, and two (2%) required fasciocutaneous flaps.

The other five studies (39 children) used only debridement and further surgery. All of those 39 children had full thickness skin injury / skin necrosis at presentation. 30 (77%) underwent mechanical debridement, of whom 22 (56%) required further operations including skin grafts and fasciocutaneous flaps and two developed contractures. The remaining nine children (23%), all from one study, underwent Versajet hydrosurgery with good healing in all children and one requiring sharp debridement.

In total, of the 137 children in the debridement and further surgery group, 103 (75%) developed skin necrosis, all of whom (in addition to some of those who were yet to develop necrosis, total n=123 (89%)) received either mechanical or water jet debridement. At least 38 children (28%) went on to require further operations.

Hyaluronidase injections

The final group of studies used hyaluronidase injections at the extravasation site without the need for incisions or washouts. This group comprised six studies and 327 children. [14,24,26–28,34]

97 children received hyaluronidase injections, one (1%) of whom had skin necrosis at presentation that resolved, and one (1%) developed necrosis requiring further surgery. Of the remaining 230 children that did not receive hyaluronidase, none developed skin necrosis at any stage, although reporting on the extent of skin damage and eventual outcomes across the studies was generally poor.

For peer review only

Comparison of the interventions

The Gault technique

Among the studies included in this review, Ghanem et al [11] provides the closest approximation of a comparative study of the Gault technique in children: 48 extravasations were diagnosed and children were divided into early (<24 hours, n=45) and later (>24 hours, n=3) referrals. Among early referrals, 22 were deemed to be 'at risk' injuries and received washout using Gault's technique. Skin necrosis occurred in one of 45 early referrals but in two of three later referrals. The conclusion of this study, that the incidence of necrosis is higher in the later referral group, is limited by low methodological quality including the use of an arbitrary 24-hour cut-off, the small size of the later referral group and a lack of data describing the types of vesicants. It is important to note that the necrosis resolved with conservative management in all three cases.

Of the 183 children treated with a variation of the Gault technique across the included studies, 12 (7%) developed skin necrosis (none had skin necrosis at presentation). This compares favourably to the 11% (10/95) of children who received no treatment across those same studies. However, 73% (69/95) of the untreated group were late referrals and may be expected to have worse outcomes related to time of presentation. The only study to apply the Gault technique in which plastic surgery review time and patient ages were similar was Ching, Wong and Milroy. [32] In that study, none of the 43 children treated with Gault's technique and hyaluronidase developed any complications whereas there were three cases of associated infection, one ischaemic toe requiring amputation and one case of calcinosis cutis amongst the 26 children that received no treatment.

The degree of injury was only recorded for 36 (13%) of the 279 children in the included studies describing the Gault technique, of which 22 (8%) were full-thickness skin injuries.

Debridement and further surgery

73 children (53%) of the 137 in the debridement and further surgery studies had at least skin necrosis / full thickness skin injury at presentation

For peer review only

In contrast to the Gault as well as the hyaluronidase injection studies, the degree of injury was documented for 93% of the children (128/137).

Hyaluronidase injections

One (0.3%) of the 327 children reported in studies describing hyaluronidase injections had skin necrosis at presentation, and one (0.3%) went on to develop it. The degree of injury was recorded for only 49 (15%) of the 327 children. The reporting of patient outcomes was particularly scarce in this group.

Injuries

The types of injury were recorded for 212 of the 728 children across the included studies. Injuries were limited to swelling, pain or erythema (n=63; 30%), partial-thickness skin injury such as superficial blistering in others (n=29; 14%) and full-thickness skin injury such as necrosis in the largest number of children (n=110; 52%). However, these figures are skewed by the fact that 10 studies only looked at extravasation injuries causing at least skin necrosis. [10,12,18–20,22,23,25,31,33] One study described a transient episode of foot ischaemia which resolved with conservative management and one compartment syndrome of the left forearm requiring fasciotomies. [12] Two studies described toe amputations secondary to ischaemia. [4,32] One study of major injuries with full-thickness tissue loss had several casualties including a below elbow amputation and two episodes of extensor loss. [20] Table 5 demonstrates the huge range of injury severity encountered and the number of each in the different intervention groups.

For peer review only - http://bmjopen.bmj.com/site/about/guidelines.xhtml

Table 5 – Injuries in those papers that recorded them

		Skin changes			Other injuries				
**Ref**	**Author**	**Pain/swelling/erythema**	**Partial thickness skin injury**	**Full thickness skin injury**	**Tendon**	**Nerve**	**Vascular**	**Compartment Syndrome**	**Amputation**
**Gault Technique Studies**									
4	Murphy	-	-	3	-	-	-	-	1
11	Casanova	10	1	3	-	-	-	-	-
16	Ching	-	-	2	-	-	-	-	1
19	Ghanem	-	-	3	-	-	-	-	
20	Kostogloudis	-	-	6	-	-	1	1	-
25	Andrés	-	-	8	-	-	-	-	-
	**Total**	**10**	**1**	**25**	**0**	**0**	**1**	**1**	**2**
**Debridement + further surgery**									
10	Falcone	-	-	16	-	-	-	-	-
13	Cho	1	2	3	-	-	-	-	-
14	Onesti	-	18	8	-	-	-	-	-
15	Firat	-	-	9	-	-	-	-	-
17	Sivrioglu	-	-	9	-	-	-	-	-
21	Sung	1	2	9	-	-	-	-	-
23	Compaña	-	1	3	-	-	-	-	-
25	Andrés (2)	-	-	8	-	-	-	-	-
26 27	Linder	-	-	18	-	-	-	-	-
28	Upton	-	-	7	2	-	-	-	1
29	von Heimburg	-	-	5	-	-	-	-	-
30	Weiss	-	-	4	-	-	-	-	-
32	Boyar	-	-	4	-	-	-	-	
	**Total**	**2**	**23**	**103**	**2**	**0**	**0**	**0**	**1**
**Hyaluronidase injections**									
12	Cochran	21	1	-	-	-	-	-	-
18	Yan (2014)	5	3	1	-	-	-	-	-
33	Yan (2017)	17	-	1	-	-	-	-	-
	**Total**	**43**	**4**	**2**	**0**	**0**	**0**	**0**	**0**
	**Overall Totals**	**55**	**28**	**122**	**2**	**0**	**1**	**1**	**3**

For peer review only

For peer review only - http://bmjopen.bmj.com/site/about/guidelines.xhtml

Millam's grading system for extravasation injuries

Several of the included studies [12,14,21,26,29,33] make reference to the four stages of extravasation/infiltration severity, which was first published by Millam in 1988. [43] This focuses on presence of pain, erythema, oedema, capillary refill, skin temperature and breakdown and it has been proposed that stages III & IV may require intervention. [44]

In one case series, [26] treatment was determined by the severity of extravasation injury based on a grading system similar to Millam's. Of the 18 children, the seven with Grade I and II injuries were managed conservatively. Ten children had grade III injuries – five were managed conservatively but the other five who had also developed skin erythema received active treatment with hyaluronidase injections. The one child with a grade IV injury required excision of the lesion – the sole surgical intervention in that study – after having been injected with hyaluronidase. All wounds healed with 'good outcomes'.

Study quality assessment

The one before-and-after study [24] was evaluated using a separate NIH tool from the case series. [9] It was given an overall 'fair' rating by both authors as it had clearly described objectives, interventions and outcome measures with a large sample size but lacked details on participant eligibility and individual level data.

In our review, for a case series to achieve a rating of 'good', it must include a clearly defined study population with comparable subjects as well as clearly described interventions, outcome measures and results with adequate follow-up (questions 2, 4, 5, 6, 7 and 9 in Table 6). If the study lacked one of these criteria, it was rated as 'fair' but if it lacked two or more, it was deemed 'poor' and at high risk of methodological bias.

11 of the 25 included case series and the Gault study itself were rated 'poor', six were 'fair' and eight 'good'.

[Table 6]

For peer review only - http://bmjopen.bmj.com/site/about/guidelines.xhtml

Table 6 – Quality assessment for included studies

Key	Y	N	–
	Yes	No	N/A, Not Recorded, or Cannot Determine

Case series

		Questions										
Ref	Study	1	2	3	4	5	6	7	8	9	Overall rating	Level of evidence
4	Murphy	Y	Y	N	Y	Y	Y	Y	–	Y	Good	4
10	Falcone	Y	Y	N	Y	Y	N	Y	–	Y	Fair	4
11	Casanova	Y	Y	N	Y	Y	N	N	–	Y	Poor	4
12	Cochran	Y	N	N	Y	Y	N	N	–	N	Poor	4
13	Cho	Y	Y	N	Y	Y	N	Y	–	Y	Fair	4
14	Onesti	N	Y	N	Y	Y	Y	Y	–	Y	Good	4
15	Firat	Y	Y	N	Y	Y	N	Y	–	Y	Fair	4
16	Ching	Y	Y	Y	Y	Y	N	N	–	Y	Poor	4
17	Sivrioglu	Y	Y	N	Y	Y	N	Y	–	Y	Fair	4
18	Yan 2014	Y	Y	N	Y	Y	Y	Y	–	Y	Good	4
19	Ghanem	Y	Y	N	Y	N	N	Y	–	Y	Poor	4
20	Kostogloudis	Y	Y	N	Y	Y	Y	Y	–	Y	Good	4
21	Sung	Y	Y	N	Y	Y	N	Y	–	Y	Fair	4
22	Odom	Y	Y	N	Y	N	Y	N	–	Y	Poor	4
23	Compaña	N	N	N	Y	N	N	–	–	Y	Poor	4
24	Harris	N	N	Y	–	Y	N	–	–	Y	Poor	4
25	Andrés	Y	N	Y	N	Y	Y	–	–	Y	Poor	4
26	Linder 1983	N	Y	Y	Y	Y	Y	Y	–	Y	Good	4
27	Linder 1985	N	Y	Y	Y	Y	Y	Y	–	Y	Good	4
28	Upton	Y	N	Y	Y	Y	N	Y	–	Y	Poor	4
29	von Heimburg	N	N	Y	N	Y	N	Y	–	Y	Poor	4
30	Weiss	N	Y	Y	Y	Y	N	Y	–	Y	Fair	4
32	Boyar	Y	Y	N	Y	Y	Y	Y	–	Y	Good	4
33	Yan 2017	Y	N	Y	–	Y	N	Y	Y	Y	Poor	4
34	Myers	Y	Y	Y	Y	Y	Y	Y	–	Y	Good	4
5	Gault	N	N	Y	N	Y	Y	N	–	Y	Poor	4

Before-and-after study

Ref	Study	1	2	3	4	5	6	7	8	9	10	11	12	Overall rating	Level of evidence
31	Hanrahan	Y	N	–	–	Y	Y	Y	N	–	Y	N	N	Fair	3

Case series questions

1. Was the study question or objective clearly stated?
2. Was the study population clearly and fully described, including a case definition?
3. Were the cases consecutive?
4. Were the subjects comparable?
5. Was the intervention clearly described?
6. Were the outcome measures clearly defined, valid, reliable, and implemented consistently across all study participants?
7. Was the length of follow-up adequate?
8. Were the statistical methods well described?
9. Were the results well described?

Before-and-after study questions

1. Was the study question or objective clearly stated?
2. Were eligibility/selection criteria for the study population prespecified and clearly described?
3. Were the participants in the study representative of those who would be eligible for the test/service/intervention in the general or clinical population of interest?
4. Were all eligible participants that met the prespecified entry criteria enrolled?
5. Was the sample size sufficiently large to provide confidence in the findings?
6. Was the test/service/intervention clearly described and delivered consistently across the study population?
7. Were the outcome measures prespecified, clearly defined, valid, reliable, and assessed consistently across all study participants?
8. Were the people assessing the outcomes blinded to the participants' exposures/interventions?
9. Was the loss to follow-up after baseline 20% or less? Were those lost to follow-up accounted for in the analysis?
10. Did the statistical methods examine changes in outcome measures from before to after the intervention? Were statistical tests done that provided p values for the pre-to-post changes?
11. Were outcome measures of interest taken multiple times before the intervention and multiple times after the intervention (i.e., did they use an interrupted time-series design)?
12. If the intervention was conducted at a group level (e.g., a whole hospital, a community, etc.) did the statistical analysis take into account the use of individual-level data to determine effects at the group level?

For peer review only

Discussion

This paper describes a systematic review of characteristics and operative management of paediatric extravasation injuries. Our review highlighted three key issues. Firstly, surgical management is commonly reported in the literature in cases where there is significant soft tissue injury but as there are no comparative studies, it is unclear whether this is optimal. Secondly, there are no data to support one surgical management approach over another, or over no treatment. Thirdly, there are no data on the adverse events caused by the different surgical interventions.

A variation of the Gault technique was used in the largest number of children to receive surgical treatment across the included studies. Gault [4] described a retrospective case series of 96 patients, ranging in age from newborn to 70 years, who were split into two groups: those seen within 24 hours of the extravasation and those seen later than this. Patients seen within 24 hours had treatment as per the Gault technique while those seen after 24 hours had no treatment. The original Gault paper was not included in our review because there were no separate paediatric data available but a separate study quality assessment by two authors rated the study as 'poor' due to the lack of a clearly defined objective or population, subjects that were not comparable and an inadequate length of follow-up. [Table 6] Despite the inherent limitations in the original Gault study, the method he described is widely used, as demonstrated by this review where it was a commonly described technique to manage extravasation injuries. This approach is not without downsides: it is an invasive procedure with associated morbidity such as pain and possible tendon and nerve damage, and takes considerable time to perform.

Evidence for the continuing use of the "Gault technique"

The degree of injury was recorded for 13% of the children included in the Gault technique studies, 93% of those in the debridement and further surgery group and 15% of those in the hyaluronidase injection group. These figures demonstrate some of the difficulties faced in drawing comparisons between different

For peer review only - http://bmjopen.bmj.com/site/about/guidelines.xhtml

interventions, both due to the poor recording of injuries and because of the fact that accurate documentation of injury severity appears to be more consistently applied following more severe injuries.

It was not possible to perform pooled analysis from included studies of Gault and alternative techniques due to study heterogeneity and incomplete outcome reporting.

All but one of the included studies were case series and the one before-and-after study [24] primarily examined the effect of new extravasation treatment guidelines on knowledge of hyaluronidase, extravasation incident reporting and time from extravasation discovery to treatment. Therefore, even though they found similar harm scores for the hyaluronidase and the non-hyaluronidase groups, the results could not be relied upon to assess the effectiveness of the intervention.

In addition, inclusion criteria varied considerably, details of the injuries were poorly described, and no studies made a comparison to another intervention. Finally, detailed outcome measures following interventions were generally lacking – many studies used subjective descriptions (such as ‘healed completely’, ‘healed fully’ or ‘no hypertrophic scarring’) on which it is difficult to base decisions in clinical practice. In summarizing the included studies, it can be noted that despite widespread use, there is no high-quality evidence to support the Gault approach or its modifications.

The future of extravasation management

A key conclusion from this review is that resolution of extravasation injuries is common regardless of the treatment applied. In the absence of data to support one treatment modality over another, it is imperative that high quality methodology randomized comparisons are undertaken; however, given the high rate of resolution with conservative treatment, any such trial will require a large, collaborative and multidisciplinary approach. Although many of the in the included studies required minimal intervention, full-thickness skin injury was commonplace; this has the potential for lifelong scarring in patients with an immature skin barrier such as children and preterm infants in particular. More serious complications were rare in but given that only a minority of studies reported on longer-term outcomes, these may underestimate the true burden of

For peer review only - http://bmjopen.bmj.com/site/about/guidelines.xhtml

extravasation injuries. We suggest the creation of a core outcome set for extravasation injury treatment and that future studies use longer-term follow-up to monitor those outcomes.

Creation of a centralized extravasation register would permit larger-scale assessment of the available interventions and the use of a universal grading scheme (such as Millam's [43]) to assess the initial extent of injuries would also aid in their comparison. However, this requires meticulous documentation of the appearance of the extravasated site as well as the extent of tissue damage.

This review was limited by the low methodological quality of included studies and inconsistent outcome reporting. Variability in baseline data, intervention data and outcome data precluded formal meta-analysis. These limitations prevent the authors from providing definitive clinical recommendations either for or against paediatric extravasation, based on the current evidence.

Conclusion

There is a paucity of evidence to inform surgical treatment of paediatric and neonatal extravasation injuries. The Gault technique is a commonly described approach, but we did not identify any evaluation of its effectiveness compared to other approaches or conservative management. A centralized extravasation register, use of a standardized and agreed grading scheme, development of a core outcome set and adequate follow-up of extravasation injuries are required to provide incidence and outcome data for this condition. This would inform a much-needed, definitive trial of therapeutic approaches to extravasation injuries in children and neonates.

For peer review only

Author Contributions

Max Little was one of the authors to independently assess eligibility of the title and abstracts of the search results. He then aided in extraction and analysis of the relevant data from included studies, performed the study quality assessments and drafted all of the versions of the article leading up to this submission.

Sophie Dupré also aided in the extraction and analysis of the relevant data from included studies, performed the study quality assessments and assisted in the drafting of each version of the article.

Justin Wormald made substantial contributions to the design of the study, aided in the extraction and analysis of the relevant data, as well as reviewing and critically editing each version of the article.

Matthew Gardiner made substantial contributions to the design of the study as well as reviewing and critically editing each version of the article.

Chris Gale acted as one of the co-senior authors, lending his expertise in paediatrics. He reviewed and critically edited the later versions of the article, substantially changing the text and ensuring its accuracy.

Abhilash Jain acted as the other co-senior author, lending his expertise in plastic surgery. He made substantial contributions to the design of the study, aided in the analysis and interpretation of the data from included studies and critically reviewed each version of the article.

Conflict of interest: CG has received support from Chiesi Pharmaceuticals to attend an educational conference; in the past 5 years he been investigator on received research grants from Medical Research Council, National Institute of Health Research, Canadian Institute of Health Research, Department of Health in England, Mason Medical Research Foundation, Westminster Medical School Research Trust and Chiesi Pharmaceuticals; he declares no other conflict of interest.

Funding: JW is an NIHR Academic Clinical Fellow; Chris Gale is funded by a Medical Research Council (MRC) Clinician Scientist Fellowship. This research is supported by the National Institute for Health Research (NIHR) infrastructure at NDORMS. The views expressed are those of the author(s) and not necessarily those

For peer review only

of the NHS, the NIHR or the Department of Health. No funding was directly received for the development of this manuscript.

Data availability: All data are included in the article or uploaded as supplementary information. No additional data are available.

For peer review only

For peer review only - http://bmjopen.bmj.com/site/about/guidelines.xhtml

References

1. Doellman D, Hadaway L, Bowe-Geddes LA, et al. Infiltration and extravasation: Update on prevention and management. *J Infus Nurs*. 2009;32(4):203-211. doi:10.1097/NAN.0b013e3181aac042
2. Paquette V, McGloin R, Northway T, DeZorzi P, Singh A, Carr R. Describing intravenous extravasation in children (DIVE study). *Can J Hosp Pharm*. 2011;64(5):340-345.
3. Wilkins C, Emmerson A. Extravasation injuries on regional neonatal units. *Arch Dis Child Fetal neonatal Ed*. 2004;89(3).
4. Murphy A, Gilmour R, Coombs C. Extravasation injury in a paediatric population. *ANZ J Surg*. 2017.
5. Gault D. Extravasation injuries. *Br J Plast Surg*. 1993;46(2):91.
6. Corbett M, Marshall D, Harden M, Sam O, Phillips R, McGuire W. Treatment of extravasation injuries in infants and young children: a scoping review and survey. Southampton (UK): NIHR Journals Library. *Health Technol Assess (Rockv)*. 2018;22(46). doi:10.3310/hta22460
7. Moher D, Liberati A, Tetziaff J, Altman D. Preferred Reporting Items for Systematic Reviews and Meta-Analyses. The PRISMA Statement. *PLoS Med*. 2009;6(7):e1000097. doi:10.1371/journal.pmed1000097
8. Higgins J, Green S. Cochrane Handnook for Systematic Reviews of Interventions Version 5.1.0 [updated March 2011]. *Cochrane Collab*. 2011. http://handbook.cochrane.org.
9. NIH Study Quality Assessment Tools. https://www.nhlbi.nih.gov/health-topics/study-quality-assessment-tools. Accessed March 25, 2019.
10. Falcone P, Barrall D, Jeyarajah D, Grossman J. Nonoperative management of full-thickness intravenous extravasation injuries in premature neonates using enzymatic debridementtle. *Ann Plast Surg*. 1989;22(2):146.
11. Ghanem M, Mansour A, Exton R, et al. Childhood extravasation injuries: improved outcome following the introduction of hospital-wide guidelines. *J Plast Reconstr Aesthetic Surg*. 2015;68(4):505.
12. Kostogloudis N, Demiri E, Tsimponis A, et al. Severe Extravasation Injuries in Neonates: A Report of 34 Cases. *Pediatr Dermatol*. 2015;32(6):830.
13. Sung K-Y, Lee S-Y. Nonoperative Management of Extravasation Injuries Associated With Neonatal Parenteral Nutrition Using Multiple Punctures and a Hydrocolloid Dressing. *Wounds A Compend Clin Res Pract*. 2016;28(5):145-152.
14. Odom B, Lowe L, Yates C. Peripheral Infiltration and Extravasation Injury Methodology: A Retrospective Study. *J Infus Nurs*. 2018;41(4):247-252.
15. Pacheco Compaña F, Midón Míguez J, de Toro Santos F. Lesions Associated With Calcium Gluconate Extravasation. *Ann Plast Surg*. 2017;79(5):444-449.
16. Harris P, Bradley S, Moss A. Limiting the damage of iatrogenic extravasation injury in neonates. *Plast Reconstr Surg*. 2001;107(3):893-894. doi:https://doi.org/10.1097/00006534-200103000-00053
17. Andrés A, Burgos L, López Gutiérrez J, et al. Treatment protocol for extravasation lesions. *Cir Pediatr*. 2006;19(3):136-139.
18. Linder R, Upton J, Osteen R. Management of extensive doxorubicin hydrochloride extravasation injuries. *J Hand Surg Am*. 1983;8(1):32-38. doi:https://doi.org/10.1016/S0363-5023(83)80048-3
19. Linder R, Upton J. Prevention of extravasation injuries secondary to doxorubicin. *Postgrad Med J*. 1985;77(4):105-9,112,114. doi:https://doi.org/10.1080/00325481.1985.11698920
20. Upton J, Mulliken J, Murray J. Major intravenous extravasation injuries. *Am J Surg*. 1979;137(4):497-506. doi:https://doi.org/10.1016/0002-9610(79)90121-1

For peer review only - http://bmjopen.bmj.com/site/about/guidelines.xhtml

21. Casanova D, Bardot J, Magalon G. Emergency treatment of accidental infusion leakage in the newborn: Report of 14 cases. *Br J Plast Surg*. 2001;54(5):396-399.
22. von Heimburg D, Pallua N. Early and late treatment of iatrogenic injection damage. *Chirurg*. 1998;69(12):1378-1382. doi:https://doi.org/10.1007/s001040050588
23. Weiss Y, Ackerman C, Shmilovitz L. Localized necrosis of scalp in neonates due to calcium gluconate infusions: a cautionary note. *Pediatrics*. 1975;56(6):1084-1086.
24. Hanrahan K. Hyaluronidase for treatment of intravenous extravasations: implementation of an evidence-based guideline in a pediatric population. *J Spec Pediatr Nurs*. 2013;18(3):253-262. doi:https://doi.org/10.1111/jspn.12035
25. Boyar V, Galiczewski C. Efficacy of Dehydrated Human Amniotic Membrane Allograft for the Treatment of Severe Extravasation Injuries in Preterm Neonates. *Wounds*. 2018;30(8):224-228.
26. Yan Y, Gong M, Chen J, Li D, Xu T, Zou H. Incidence, risk factors and treatment outcomes of drug extravasation in pediatric patients in China. *Turk J Pediatr*. 2017;(59):162-168.
27. Myers P, Krasniak P, Bell D. Managing intravenous infiltration injuries in the neonatal intensive care unit. *J Burn Care Res*. 2018;39(9):S151.
28. Cochran T, Bomyea K, Kahn M. Treatment of iodinated contrast material extravasation with hyaluronidase. *Acad Radiol*. 2002;9.
29. Cho K, Lee S, Burm J, Park E. Successful combined treatment with total parenteral nutrition fluid extravasation injuries in preterm infants. *J Korean Med Sci*. 2007;22(3):588.
30. Onesti M, Carella S, Maruccia M, Ciotti M, Scuderi N. The use of hyalomatrix PA in the treatment of extravasation affecting premature neonates. *Plast Reconstr Surg*. 2012;129(1):219e-21e.
31. Firat C, Erbatur S, Aytekin A. Management of extravasation injuries: a retrospective study. *J Plast Surg Hand Surg*. 2013;47(1):60.
32. Ching L, Wong K, Milroy C. Paediatric extravasation injuries: a review of 69 consecutive patients. *Int J Surg*. 2014;12(10):1036.
33. Sivrioğlu N, Irkören S. Versajet hydrosurgery system in the debridement of skin necrosis after calcium gluconate extravasation: report of 9 infantile cases. *Acta Orthop Traumatol Turc*. 2014;48(1):6.
34. Yan Y, Fan Q, Li A, Chen J, Dong F, Gong M. Treatment of cutaneous injuries of neonates induced by drug extravasation with hyaluronidase and hirudoid. *Iran J Pediatr*. 2014;24(4):352-358.
35. Loth T, Eversmann W. Extravasation injuries in the upper extremity. *Clin Orthop Relat Res*. 1991;(272):48.
36. Scuderi N, Onesti M. Antitumor agents: extravasation, management, and surgical treatment. *Ann Plast Surg*. 1994;32(1):39.
37. Langstein N, Duman H, Seelig D, Butler E, Evans R. Retrospective study of the management of chemotherapeutic extravasation injury. *Ann Plast Surg*. 2002;49(4):369.
38. D'Andrea F, Onesti M, Nicoletti G, et al. Surgical treatment of ulcers caused by extravasation of cytotoxic drugs. *Scand J Plast Reconstr Surg Hand Surg*. 2004;38(5):288-292.
39. Rose R, Felix R, Crawford-Sykes A, Venugopal R, Wharfe G, Arscott G. Extravasation injuries. *West Indian Med J*. 2008;57(1):40.
40. Sbitany H, Koltz F, Mays C, Girotto A, Langstein N. CT contrast extravasation in the upper extremity: strategies for management. *Int J Surg*. 2010;8(5):384.
41. Kim S, Cook K, Lee I, Park D. Computed tomography contrast media extravasation: treatment algorithm and immediate treatment by squeezing with multiple slit incisions. *Int Wound J*. 2017;14(2):430-434.
42. Onesti M, Carella S, Fioramonti P, Scuderi N. Chemotherapy Extravasation Management: 21-Year Experience. *Ann Plast Surg*. 2017;79(5):450-457.

For peer review only

43. Millam D. Managing complications of IV therapy. *Nursing (Lond)*. 1988;(18):34-43.
44. Flemmer L, Chan J. A pediatric protocol for management of extravasation injuries. *Pediatr Nurs*. 1993;18:44-47.

For peer review only

Search Results

Table of Contents

Search History .... page 2

1. Thoracic and abdominal extravasation: a complication of hyperalimentation in infants. .... page 4

2. [Traumatic cerebral aneurysm showing remarkable extravasation into the lateral ventricle--report of a case in an infant (author's transl)]. .... page 4

3. Urinary ascites in children owing to extravasation of urine from the bladder. .... page 4

4. Bone scintigraphy for neonatal osteomyelitis: simulation by extravasation of intravenous calcium. .... page 4

5. Perirenal urinary extravasation associated with urethral valves in infants. .... page 4

6. Nafcillin extravasation injury. Use of hyaluronidase as an antidote. .... page 4

7. Cutaneous calcinosis in a neonate following extravasation of calcium gluconate. .... page 4

8. Urinary extravasation in the newborn male with posterior urethral valves. .... page 4

9. Intrathoracic extravasation of sclerosing agents associated with central venous catheters. .... page 5

10. Nonoperative management of full-thickness intravenous extravasation injuries in premature neonates using enzymatic debridement. .... page 5

For peer review only

Search History

1. AMED, EMBASE, Medline, CINAHL; p?ediatric*.ti,ab; 2702402 results.
2. AMED, EMBASE, Medline, CINAHL; neonat*.ti,ab; 508216 results.
3. AMED, EMBASE, Medline, CINAHL; infant*.ti,ab; 761110 results.
4. AMED, EMBASE, Medline, CINAHL; child.ti,ab; 737276 results.
5. AMED, EMBASE, Medline, CINAHL; 1 OR 2 OR 3 OR 4; 4209881 results.
6. AMED, EMBASE, Medline, CINAHL; extra?vasat*.ti,ab; 19189 results.
7. AMED, EMBASE, Medline, CINAHL; extravasation.ti,ab; 30514 results.
8. AMED, EMBASE, Medline, CINAHL; 6 OR 7; 32931 results.
9. AMED, EMBASE, Medline, CINAHL; 5 AND 8; 1849 results.
10. AMED, EMBASE, Medline, CINAHL; outcome*.ti,ab; 2946939 results.
11. AMED, EMBASE, Medline, CINAHL; PROM*.ti,ab; 2921492 results.
12. AMED, EMBASE, Medline, CINAHL; patient?related*.ti,ab; 131 results.
13. AMED, EMBASE, Medline, CINAHL; patient?report*.ti,ab; 689 results.
14. AMED, EMBASE, Medline, CINAHL; 10 OR 11 OR 12 OR 13; 5654365 results.
15. AMED, EMBASE, Medline, CINAHL; conservative*.ti,ab; 231035 results.
16. AMED, EMBASE, Medline, CINAHL; non?operative*.ti,ab; 11082 results.
17. AMED, EMBASE, Medline, CINAHL; non?surg*.ti,ab; 14737 results.
18. AMED, EMBASE, Medline, CINAHL; cold*.ti,ab; 225847 results.
19. AMED, EMBASE, Medline, CINAHL; cool*.ti,ab; 101309 results.
20. AMED, EMBASE, Medline, CINAHL; hot*.ti,ab; 160999 results.
21. AMED, EMBASE, Medline, CINAHL; warm*.ti,ab; 110969 results.
22. AMED, EMBASE, Medline, CINAHL; elevat*.ti,ab; 1359688 results.
23. AMED, EMBASE, Medline, CINAHL; dress*.ti,ab; 53707 results.
24. AMED, EMBASE, Medline, CINAHL; 15 OR 16 OR 17 OR 18 OR 19 OR 20 OR 21 OR 22 OR 23; 2170626 results.
25. AMED, EMBASE, Medline, CINAHL; medic*.ti,ab; 3892625 results.
26. AMED, EMBASE, Medline, CINAHL; steroid*.ti,ab; 449723 results.
27. AMED, EMBASE, Medline, CINAHL; cortico?steroid*.ti,ab; 114994 results.
28. AMED, EMBASE, Medline, CINAHL; corticosteroid*.ti,ab; 206312 results.
29. AMED, EMBASE, Medline, CINAHL; sub?cut*.ti,ab; 245741 results.
30. AMED, EMBASE, Medline, CINAHL; subcut*.ti,ab; 189955 results.
31. AMED, EMBASE, Medline, CINAHL; inject*.ti,ab; 1404121 results.
32. AMED, EMBASE, Medline, CINAHL; topical*.ti,ab; 199318 results.
33. AMED, EMBASE, Medline, CINAHL; cream*.ti,ab; 38332 results.
34. AMED, EMBASE, Medline, CINAHL; ointment*.ti,ab; 24526 results.
35. AMED, EMBASE, Medline, CINAHL; emollient*.ti,ab; 3696 results.
36. AMED, EMBASE, Medline, CINAHL; antidote*.ti,ab; 11910 results.
37. AMED, EMBASE, Medline, CINAHL; Sodium?thiosulfate*.ti,ab; 6 results.
38. AMED, EMBASE, Medline, CINAHL; mechlorethamine*.ti,ab; 1486 results.
39. AMED, EMBASE, Medline, CINAHL; Dexrazoxane*.ti,ab; 1175 results.
40. AMED, EMBASE, Medline, CINAHL; N?acetylcysteine*.ti,ab; 1604942 results.
41. AMED, EMBASE, Medline, CINAHL; vitamin?C*.ti,ab; 422 results.
42. AMED, EMBASE, Medline, CINAHL; ascorbic*.ti,ab; 58559 results.
43. AMED, EMBASE, Medline, CINAHL; basic AND fibroblast AND growth AND factor.ti,ab; 29979 results.
44. AMED, EMBASE, Medline, CINAHL; granulocyte AND macrophage AND colony AND stimulating AND factor.ti,ab; 37389 results.
45. AMED, EMBASE, Medline, CINAHL; hyaluroni*.ti,ab; 42841 results.
46. AMED, EMBASE, Medline, CINAHL; salin*.ti,ab; 375104 results.
47. AMED, EMBASE, Medline, CINAHL; 25 OR 26 OR 27 OR 28 OR 29 OR 30 OR 31 OR 32 OR 33 OR 34 OR 35 OR 36 OR 37 OR 38 OR 39 OR 40 OR 41 OR 42 OR 43 OR 44 OR 45 OR 46; 7833762 results.
48. AMED, EMBASE, Medline, CINAHL; squeeze*.ti,ab; 9290 results.
49. AMED, EMBASE, Medline, CINAHL; surg*.ti,ab; 3592311 results.
50. AMED, EMBASE, Medline, CINAHL; intervention*.ti,ab; 1719913 results.
51. AMED, EMBASE, Medline, CINAHL; irrigation*.ti,ab; 45442 results.
52. AMED, EMBASE, Medline, CINAHL; excis*.ti,ab; 326226 results.
53. AMED, EMBASE, Medline, CINAHL; infiltrat*.ti,ab; 404118 results.
54. AMED, EMBASE, Medline, CINAHL; therapeut*.ti,ab; 1901755 results.
55. AMED, EMBASE, Medline, CINAHL; manage*.ti,ab; 2431717 results.
56. AMED, EMBASE, Medline, CINAHL; treatment*.ti,ab; 8388485 results.

For peer review only

57. AMED, EMBASE, Medline, CINAHL; wash?out*.ti,ab; 28666 results.
58. AMED, EMBASE, Medline, CINAHL; operat*.ti,ab; 2000359 results.
59. AMED, EMBASE, Medline, CINAHL; bedside*.ti,ab; 57042 results.
60. AMED, EMBASE, Medline, CINAHL; liposuct*.ti,ab; 5719 results.
61. AMED, EMBASE, Medline, CINAHL; fasciotom*.ti,ab; 5310 results.
62. AMED, EMBASE, Medline, CINAHL; 48 OR 49 OR 50 OR 51 OR 52 OR 53 OR 54 OR 55 OR 56 OR 57 OR 58 OR 59 OR 60 OR 61; 15163731 results.
63. AMED, EMBASE, Medline, CINAHL; 9 AND 24; 222 results.
64. AMED, EMBASE, Medline, CINAHL; 9 AND 47; 982 results.
65. AMED, EMBASE, Medline, CINAHL; 9 AND 62; 1130 results.
66. AMED, EMBASE, Medline, CINAHL; 24 OR 47 OR 62; 20658308 results.
67. AMED, EMBASE, Medline, CINAHL; 9 AND 66; 1455 results.
68. AMED, EMBASE, Medline, CINAHL; 67 NOT animal; 1176 results.
69. AMED, EMBASE, Medline, CINAHL; 68 NOT laboratory; 1117 results.
70. AMED, EMBASE, Medline, CINAHL; 69 NOT adult; 804 results.

For peer review only

Evidence Services | library.nhs.uk

1. Thoracic and abdominal extravasation: a complication of hyperalimentation in infants.

**Citation:**	AJR. American journal of roentgenology, Mar 1977, vol. 128, no. 3, p. 419-422, 0361-803X (March 1977)
**Author(s):**	Spriggs, D W; Brantley, R E
**Source:**	Medline

2. [Traumatic cerebral aneurysm showing remarkable extravasation into the lateral ventricle--report of a case in an infant (author's transl)].

**Citation:**	No shinkei geka. Neurological surgery, Apr 1977, vol. 5, no. 4, p. 371-378, 0301-2603 (April 1977)
**Author(s):**	Nakamura, T; Matsuoka, Y; Nishimura, S
**Source:**	Medline

3. Urinary ascites in children owing to extravasation of urine from the bladder.

**Citation:**	The Journal of urology, Sep 1979, vol. 122, no. 3, p. 409-411, 0022-5347 (September 1979)
**Author(s):**	Redman, J F; Seibert, J J; Arnold, W
**Source:**	Medline

4. Bone scintigraphy for neonatal osteomyelitis: simulation by extravasation of intravenous calcium.

**Citation:**	Radiology, Apr 1980, vol. 135, no. 1, p. 185-186, 0033-8419 (April 1980)
**Author(s):**	Balsam, D; Goldfarb, C R; Stringer, B; Farruggia, S
**Source:**	Medline

5. Perirenal urinary extravasation associated with urethral valves in infants.

**Citation:**	The Journal of urology, Nov 1980, vol. 124, no. 5, p. 688-691, 0022-5347 (November 1980)
**Author(s):**	Mitchell, M E; Garrett, R A
**Source:**	Medline

6. Nafcillin extravasation injury. Use of hyaluronidase as an antidote.

**Citation:**	American journal of diseases of children (1960), Dec 1981, vol. 135, no. 12, p. 1113-1114, 0002-922X (December 1981)
**Author(s):**	Zenk, K E; Dungy, C I; Greene, G R
**Source:**	Medline

7. Cutaneous calcinosis in a neonate following extravasation of calcium gluconate.

**Citation:**	Journal of the American Academy of Dermatology, Mar 1982, vol. 6, no. 3, p. 392-395, 0190-9622 (March 1982)
**Author(s):**	Hironaga, M; Fujigaki, T; Tanaka, S
**Source:**	Medline

8. Urinary extravasation in the newborn male with posterior urethral valves.

**Citation:**	Journal of pediatric surgery, Dec 1982, vol. 17, no. 6, p. 751-756, 0022-3468 (December 1982)
**Author(s):**	Greenfield, S P; Hensle, T W; Berdon, W E; Geringer, A M

For peer review only

Source: Medline

9. Intrathoracic extravasation of sclerosing agents associated with central venous catheters.

Citation: The American journal of pediatric hematology/oncology, Jan 1988, vol. 10, no. 3, p. 249-251, 0192-8562 (1988)

Author(s): Watterson, J; Heisel, M; Cich, J A; Priest, J R

Source: Medline

Full Text: Available from *Ovid* in *American Journal of Pediatric Hematology/Oncology*

10. Nonoperative management of full-thickness intravenous extravasation injuries in premature neonates using enzymatic debridement.

Citation: Annals of plastic surgery, Feb 1989, vol. 22, no. 2, p. 146-149, 0148-7043 (February 1989)

Author(s): Falcone, P A; Barrall, D T; Jeyarajah, D R; Grossman, J A

Source: Medline

Full Text: Available from *Ovid* in *Annals of Plastic Surgery*

For peer review only

Search Results

Table of Contents

Search History .... page 2

1. Hepatic and splenic blush on computed tomography in children following blunt abdominal trauma: Is intervention necessary? .... page 3

2. Safety and effectiveness of intravenous pentamidine for prophylaxis of Pneumocystis jirovecii pneumonia in pediatric hematology/oncology patients .... page 3

3. Efficacy, Safety, and Pharmacokinetics of a New 10 % Liquid Intravenous Immunoglobulin Containing High Titer Neutralizing Antibody to RSV and Other Respiratory Viruses in Subjects with Primary Immunodeficiency Disease .... page 3

4. A multicenter, randomized, observer-blinded, active-controlled study evaluating the safety and effectiveness of ceftaroline compared with ceftriaxone plus vancomycin in pediatric patients with complicated community-acquired bacterial pneumonia .... page 3

5. Predictive factors for conservative treatment failure in grade IV pediatric blunt renal trauma .... page 3

6. Clinical negligence claims in pediatric surgery in England: Pattern and trends .... page 3

7. Outcomes of peripherally inserted double lumen central catheter in very low birth weight infants .... page 4

8. Pelvic Artery Embolization in the Management of Obstetrical Hemorrhage: Predictive Factors for Clinical Outcomes .... page 4

9. The role of interventional radiology for pediatric blunt renal trauma .... page 4

10. Safety and efficacy of gadoteric acid in pediatric magnetic resonance imaging: overview of clinical trials and post-marketing studies .... page 4

For peer review only

For peer review only - http://bmjopen.bmj.com/site/about/guidelines.xhtml

Search History

1. EMBASE; exp PEDIATRICS/; 87850 results.
2. EMBASE; exp CHILD/; 2256483 results.
3. EMBASE; exp INFANT/; 930470 results.
4. EMBASE; exp ADOLESCENT/; 1367630 results.
5. EMBASE; exp NEWBORN/; 495069 results.
6. EMBASE; exp CONTRAST MEDIUM EXTRAVASATION/ OR exp DRUG EXTRAVASATION/ OR exp EXTRAVASATION/ OR exp INJECTION SITE EXTRAVASATION/; 11979 results.
7. EMBASE; exp OUTCOME MEASUREMENT/ OR exp OUTCOMES RESEARCH/ OR exp EVIDENCE BASED MEDICINE/ OR exp TREATMENT OUTCOME/; 1809683 results.
8. EMBASE; 1 OR 2 OR 3 OR 4 OR 5; 2973897 results.
9. EMBASE; 6 AND 8; 958 results.
10. EMBASE; 7 AND 9; 150 results.

For peer review only

For peer review only - http://bmjopen.bmj.com/site/about/guidelines.xhtml

1. Hepatic and splenic blush on computed tomography in children following blunt abdominal trauma: Is intervention necessary?

Citation: Journal of Trauma and Acute Care Surgery, 2016, vol./is. 81/2(266-270), 2163-0755;2163-0763 (2016)

Author(s): Ingram M.-C.E.; Siddharthan R.V.; Morris A.D.; Hill S.J.; Travers C.D.; McKracken C.E.; Heiss K.F.; Raval M.V.; Santore M.T.

Source: EMBASE

Full Text: Available from *Ovid* in *Journal of Trauma and Acute Care Surgery*

2. Safety and effectiveness of intravenous pentamidine for prophylaxis of Pneumocystis jirovecii pneumonia in pediatric hematology/oncology patients

Citation: Journal of Pediatric Hematology/Oncology, July 2016, vol./is. 38/6(e180-e185), 1077-4114;1536-3678 (26 Jul 2016)

Author(s): Solodokin L.J.; Klejmont L.M.; Scipione M.R.; Dubrovskaya Y.; Lighter-Fisher J.; Papadopoulos J.

Source: EMBASE

Full Text: Available from *Ovid* in *Journal of Pediatric Hematology/Oncology*

3. Efficacy, Safety, and Pharmacokinetics of a New 10 % Liquid Intravenous Immunoglobulin Containing High Titer Neutralizing Antibody to RSV and Other Respiratory Viruses in Subjects with Primary Immunodeficiency Disease

Citation: Journal of Clinical Immunology, August 2016, vol./is. 36/6(590-599), 0271-9142;1573-2592 (01 Aug 2016)

Author(s): Wasserman R.L.; Lumry W.; Harris J.; Levy R.; Stein M.; Forbes L.; Cunningham-Rundles C.; Melamed I.; Kobayashi A.L.; Du W.; Kobayashi R.

Source: EMBASE

Full Text: Available from *Springer Link Journals* in *Journal of Clinical Immunology*

4. A multicenter, randomized, observer-blinded, active-controlled study evaluating the safety and effectiveness of ceftaroline compared with ceftriaxone plus vancomycin in pediatric patients with complicated community-acquired bacterial pneumonia

Citation: Pediatric Infectious Disease Journal, July 2016, vol./is. 35/7(760-766), 0891-3668;1532-0987 (01 Jul 2016)

Author(s): Blumer J.L.; Ghonghadze T.; Cannavino C.; O'Neal T.; Jandourek A.; Friedland H.D.; Bradley J.S.

Source: EMBASE

Full Text: Available from *Ingenta* in *Pediatric Infectious Disease Journal, The*
Available from *Ovid* in *Pediatric Infectious Disease Journal*

5. Predictive factors for conservative treatment failure in grade IV pediatric blunt renal trauma

Citation: Journal of Pediatric Urology, April 2016, vol./is. 12/2(93.e1-93.e7), 1477-5131;1873-4898 (01 Apr 2016)

Author(s): Lee J.N.; Lim J.K.; Woo M.J.; Kwon S.Y.; Kim B.S.; Kim H.T.; Kim T.-H.; Yoo E.S.; Chung S.K.

Source: EMBASE

6. Clinical negligence claims in pediatric surgery in England: Pattern and trends

Citation: European Journal of Pediatric Surgery, February 2015, vol./is. 25/1(66-70), 0939-7248;1439-359X (February 2015)

For peer review only

Author(s): Thyoka M.

Source: EMBASE

7. Outcomes of peripherally inserted double lumen central catheter in very low birth weight infants

Citation: Journal of Neonatal-Perinatal Medicine, March 2016, vol./is. 9/1(99-105), 1934-5798;1878-4429 (24 Mar 2016)

Author(s): Cheong S.M.; Totsu S.; Nakanishi H.; Uchiyama A.; Kusuda S.

Source: EMBASE

8. Pelvic Artery Embolization in the Management of Obstetrical Hemorrhage: Predictive Factors for Clinical Outcomes

Citation: CardioVascular and Interventional Radiology, December 2015, vol./is. 38/6(1477-1486), 0174-1551;1432-086X (01 Dec 2015)

Author(s): Zhang E.; Liu L.; Owen R.

Source: EMBASE

Full Text: Available from *Springer Link Journals* in *CardioVascular and Interventional Radiology*

9. The role of interventional radiology for pediatric blunt renal trauma

Citation: Italian Journal of Pediatrics, October 2015, vol./is. 41/1(no pagination), 1720-8424;1824-7288 (15 Oct 2015)

Author(s): Lin W.-C.; Lin C.-H.

Source: EMBASE

Full Text: Available from *National Library of Medicine* in *Italian Journal of Pediatrics*
Available from *BioMed Central* in *Italian Journal of Pediatrics*
Available from *National Library of Medicine* in *Italian Journal of Pediatrics*
Available from *ProQuest* in *Italian Journal of Pediatrics*

10. Safety and efficacy of gadoteric acid in pediatric magnetic resonance imaging: overview of clinical trials and post-marketing studies

Citation: Pediatric Radiology, November 2015, vol./is. 45/12(1831-1841), 0301-0449;1432-1998 (01 Nov 2015)

Author(s): Balassy C.; Roberts D.; Miller S.F.

Source: EMBASE

Full Text: Available from *Springer Link Journals* in *Pediatric Radiology*

For peer review only

Search Results

Table of Contents

Search History ..... page 2

1. Emergency treatment of accidental infusion leakage in the newborn. ..... page 3

2. Childhood extravasation injuries: improved outcome following the introduction of hospital-wide guidelines. ..... page 3

3. [Treatment protocol for extravasation lesions]. ..... page 3

4. The use of central venous catheters in paediatric oncology--a cautionary tale. ..... page 3

5. A new approach to management of intravenous infiltration in pediatric patients: pathophysiology, classification, and treatment. ..... page 3

6. Saline irrigation for the management of skin extravasation injury in neonates. ..... page 3

7. Extravasation injuries. ..... page 3

8. [Central venous catheters in newborn infants. Work Group Parenteral Nutrition of the Perinatology Section of the Dutch Pediatric Society]. ..... page 4

9. Extravasation of parenteral alimentation fluid into the renal pelvis--a complication of central venous catheter in a neonate. ..... page 4

10. Percutaneous central line extravasation masquerading as an abscess. ..... page 4

For peer review only

For peer review only - http://bmjopen.bmj.com/site/about/guidelines.xhtml

Search History

1. Medline; exp PEDIATRICS/; 49428 results.
2. Medline; exp CHILD/; 1666146 results.
3. Medline; exp INFANT/; 1008023 results.
4. Medline; exp INFANT, NEWBORN/; 535610 results.
5. Medline; 1 OR 2 OR 3 OR 4; 2185064 results.
6. Medline; exp EXTRAVASATION OF DIAGNOSTIC AND THERAPEUTIC MATERIALS/; 3098 results.
7. Medline; exp "OUTCOME ASSESSMENT (HEALTH CARE)"/ OR exp PATIENT OUTCOME ASSESSMENT/ OR exp TREATMENT OUTCOME/; 815946 results.
8. Medline; exp EARLY MEDICAL INTERVENTION/; 1559 results.
9. Medline; exp THERAPEUTIC IRRIGATION/ OR exp THERAPEUTICS/; 5011881 results.
10. Medline; exp SPECIALTIES, SURGICAL/ OR exp SURGICAL PROCEDURES, OPERATIVE/; 2809893 results.
11. Medline; 8 OR 9 OR 10; 6354238 results.
12. Medline; 5 AND 6; 345 results.
13. Medline; 7 AND 12; 36 results.
14. Medline; 11 AND 12; 248 results.
15. Medline; 12 OR 13 OR 14; 345 results.

For peer review only

For peer review only - http://bmjopen.bmj.com/site/about/guidelines.xhtml

1. Emergency treatment of accidental infusion leakage in the newborn.

Citation: British journal of plastic surgery, Jan 2002, vol. 55, no. 1, p. 89., 0007-1226 (January 2002)

Author(s): Rose, Victoria; Turner, Matthew; Harris, Paul A; Moss, Anthony L H

Source: Medline

Full Text: Available from *Ovid* in *Plastic and Reconstructive Surgery*

2. Childhood extravasation injuries: improved outcome following the introduction of hospital-wide guidelines.

Citation: Journal of plastic, reconstructive & aesthetic surgery : JPRAS, Apr 2015, vol. 68, no. 4, p. 505-518, 1878-0539 (April 2015)

Author(s): Ghanem, Ali M; Mansour, Abdulrab; Exton, Rebecca; Powell, Jonathan; Mashhadi, Syed; Bulstrode, Neil; Smith, Gillian

Source: Medline

3. [Treatment protocol for extravasation lesions].

Citation: Cirugía pediátrica : organo oficial de la Sociedad Española de Cirugía Pediátrica, Jul 2006, vol. 19, no. 3, p. 136-139, 0214-1221 (July 2006)

Author(s): Andrés, A M; Burgos, L; López Gutiérrez, J C; Encinas, J L; Díaz, M; Rivas, S; Ros, Z

Source: Medline

4. The use of central venous catheters in paediatric oncology--a cautionary tale.

Citation: Scottish medical journal, Feb 1990, vol. 35, no. 1, p. 11-14, 0036-9330 (February 1990)

Author(s): Russell, L; Craig, J I; Mackinlay, G A; Eden, O B

Source: Medline

5. A new approach to management of intravenous infiltration in pediatric patients: pathophysiology, classification, and treatment.

Citation: Journal of infusion nursing : the official publication of the Infusion Nurses Society, Jul 2011, vol. 34, no. 4, p. 242-249, 1539-0667 (2011 Jul-Aug)

Author(s): Amjad, Ibrahim; Murphy, Travis; Nylander-Housholder, Linda; Ranft, Amanda

Source: Medline

Full Text: Available from *Ovid* in *Journal of Infusion Nursing*

6. Saline irrigation for the management of skin extravasation injury in neonates.

Citation: The Cochrane database of systematic reviews, Jan 2012, no. 2, p. CD008404., 1469-493X (2012)

Author(s): Gopalakrishnan, P N; Goel, N; Banerjee, Sujoy

Source: Medline

Full Text: Available from *John Wiley and Sons* in *Cochrane Library, The*

7. Extravasation injuries.

Citation: British journal of plastic surgery, Mar 1993, vol. 46, no. 2, p. 91-96, 0007-1226 (March 1993)

Author(s): Gault, D T

Source: Medline

Full Text: Available from *Ovid* in *Plastic and Reconstructive Surgery*

For peer review only

8. [Central venous catheters in newborn infants. Work Group Parenteral Nutrition of the Perinatology Section of the Dutch Pediatric Society].

**Citation:**	Tijdschrift voor kindergeneeskunde, Jun 1993, vol. 61, no. 3, p. 76-82, 0376-7442 (June 1993)
**Author(s):**	van Lingen, R A; Liem, K D; Krediet, T G
**Source:**	Medline

9. Extravasation of parenteral alimentation fluid into the renal pelvis--a complication of central venous catheter in a neonate.

**Citation:**	Journal of perinatology : official journal of the California Perinatal Association, Oct 2001, vol. 21, no. 7, p. 465-466, 0743-8346 (2001 Oct-Nov)
**Author(s):**	Nadroo, A M; al-Sowailem, A M
**Source:**	Medline
**Full Text:**	Available from *Nature Publishing Group* in *Journal of Perinatology*<br>Available from *Nature Publishing Group* in *Journal of Perinatology*<br>Available from *ProQuest* in *Journal of Perinatology*

10. Percutaneous central line extravasation masquerading as an abscess.

**Citation:**	Indian pediatrics, Apr 2014, vol. 51, no. 4, p. 309-310, 0974-7559 (April 2014)
**Author(s):**	Govind, Binu; Tete, Prakash Ignace; Thomas, Niranjan
**Source:**	Medline
**Full Text:**	Available from *Springer Link Journals* in *Indian Pediatrics*

For peer review only

Supplementary 2
Study Attrition Process

Level 1 - Is a clinical study of paediatric extravasation injury?

If yes then proceed to level 2

Level 2 - Does it report on one or more of the following outcomes?

- Short-term outcomes:
    - Time to complete tissue healing
    - Infection
    - Hypothermia
    - Anaphylactic reactions
    - Fluid and electrolyte disturbance
    - Any other adverse effects reported in the studies;
- Long-term outcomes (preferably at least three months from the injury):
    - Severity of the scar including contractures, functional impairment, disfigurement and need for further surgical procedures.

Level 3 – is it an original clinical study or systematic review of clinical studies?

If yes then proceed to level 4

Level 4 - In which of the following groups of the study types does it belong?

Group 1

- RCT
- Quasi-randomised study (e.g. alternation rather than randomisation used)
- Systematic reviews of RCTs or quasi-randomised studies

Group 2

- Cross-sectional study
- Case-series
- Systematic reviews of cross-sectional studies or case-series

If yes then retrieve full article for consideration for inclusion

For peer review only

Supplementary 3
Review-Specific Proforma

Author	Patients (n)	Age (mean)	Gender		Comorbidities				Cannula location			Cannula type	
			M	F	Premature	Sepsis	Malignancy	Other	Upper limb	Lower limb	Scalp	Peripheral	Central

Author	Vesicants							Medical Management	Surgical Management	Control (Y/N)
	TPN	≥5% Dextrose	Other IV Fluids	Calcium-containing fluids	Antibiotics	Contrast	Other			

Author	Surgical injuries								Wound outcome	Functional outcome
	Swelling/ Erythema	Partial thickness skin injury	Full thickness skin injury	Tendon injury	Nerve injury	Vascular injury	Compartment Syndrome	Amputation		

For peer review only

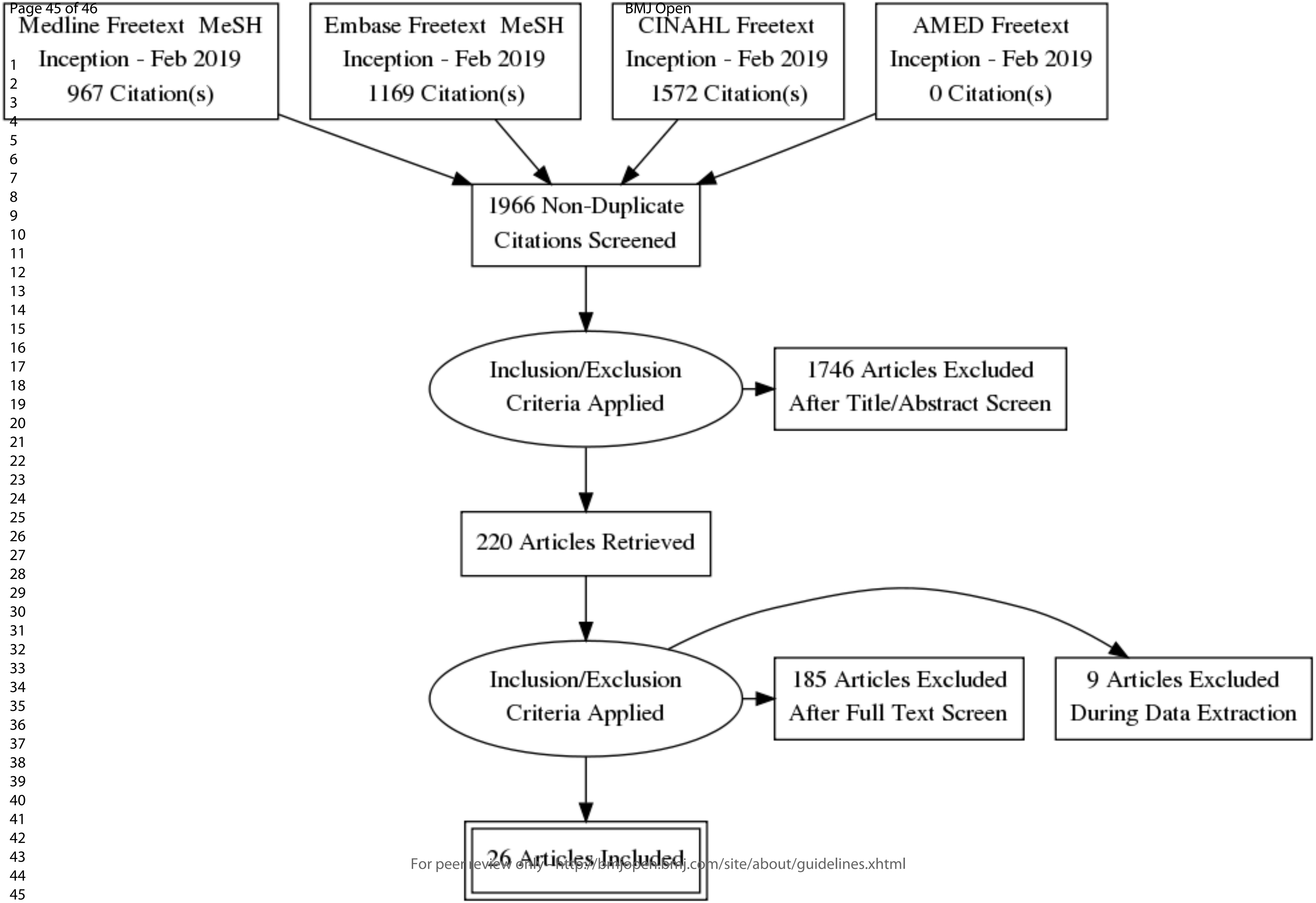
Medline Freetext MeSH
Inception - Feb 2019
967 Citation(s)
Embase Freetext MeSH
Inception - Feb 2019
1169 Citation(s)
CINAHL Freetext
Inception - Feb 2019
1572 Citation(s)
AMED Freetext
Inception - Feb 2019
0 Citation(s)
1966 Non-Duplicate
Citations Screened
Inclusion/Exclusion
Criteria Applied
1746 Articles Excluded
After Title/Abstract Screen
220 Articles Retrieved
Inclusion/Exclusion
Criteria Applied
185 Articles Excluded
After Full Text Screen
9 Articles Excluded
During Data Extraction
26 Articles Included

PRISMA 2009 Checklist – Surgical Intervention for Paediatric Extravasation

Section/topic	#	Checklist item	Reported on page #
**TITLE**			
Title	1	Identify the report as a systematic review, meta-analysis, or both.	1
**ABSTRACT**			
Structured summary	2	Provide a structured summary including, as applicable: background; objectives; data sources; study eligibility criteria, participants, and interventions; study appraisal and synthesis methods; results; limitations; conclusions and implications of key findings; systematic review registration number.	2
**INTRODUCTION**			
Rationale	3	Describe the rationale for the review in the context of what is already known.	4
Objectives	4	Provide an explicit statement of questions being addressed with reference to participants, interventions, comparisons, outcomes, and study design (PICOS).	5
**METHODS**			
Protocol and registration	5	Indicate if a review protocol exists, if and where it can be accessed (e.g., Web address), and, if available, provide registration information including registration number.	6
Eligibility criteria	6	Specify study characteristics (e.g., PICOS, length of follow-up) and report characteristics (e.g., years considered, language, publication status) used as criteria for eligibility, giving rationale.	6
Information sources	7	Describe all information sources (e.g., databases with dates of coverage, contact with study authors to identify additional studies) in the search and date last searched.	6,7,8
Search	8	Present full electronic search strategy for at least one database, including any limits used, such that it could be repeated.	Supplementary-1
Study selection	9	State the process for selecting studies (i.e., screening, eligibility, included in systematic review, and, if applicable, included in the meta-analysis).	7
Data collection process	10	Describe method of data extraction from reports (e.g., piloted forms, independently, in duplicate) and any processes for obtaining and confirming data from investigators.	Supplementary-2
Data items	11	List and define all variables for which data were sought (e.g., PICOS, funding sources) and any assumptions and simplifications made.	Supplementary-2
Risk of bias in individual studies	12	Describe methods used for assessing risk of bias of individual studies (including specification of whether this was done at the study or outcome level), and how this information is to be used in any data synthesis.	7
Summary measures	13	State the principal summary measures (e.g., risk ratio, difference in means).	N/A
Synthesis of results	14	Describe the methods of handling data and combining results of studies, if done, including measures of consistency (e.g., I^2) for each meta-analysis.	N/A

For peer review only

PRISMA 2009 Checklist – Surgical Intervention for Paediatric Extravasation

Section/topic	#	Checklist item	Reported on page #
Risk of bias across studies	15	Specify any assessment of risk of bias that may affect the cumulative evidence (e.g., publication bias, selective reporting within studies).	N/A
Additional analyses	16	Describe methods of additional analyses (e.g., sensitivity or subgroup analyses, meta-regression), if done, indicating which were pre-specified.	N/A
**RESULTS**			
Study selection	17	Give numbers of studies screened, assessed for eligibility, and included in the review, with reasons for exclusions at each stage, ideally with a flow diagram.	8
Study characteristics	18	For each study, present characteristics for which data were extracted (e.g., study size, PICOS, follow-up period) and provide the citations.	Table 1 + Table 2
Risk of bias within studies	19	Present data on risk of bias of each study and, if available, any outcome level assessment (see item 12).	Table 5
Results of individual studies	20	For all outcomes considered (benefits or harms), present, for each study: (a) simple summary data for each intervention group (b) effect estimates and confidence intervals, ideally with a forest plot.	Table 4
Synthesis of results	21	Present results of each meta-analysis done, including confidence intervals and measures of consistency.	N/A
Risk of bias across studies	22	Present results of any assessment of risk of bias across studies (see Item 15).	N/A
Additional analysis	23	Give results of additional analyses, if done (e.g., sensitivity or subgroup analyses, meta-regression [see Item 16]).	N/A
**DISCUSSION**			
Summary of evidence	24	Summarize the main findings including the strength of evidence for each main outcome; consider their relevance to key groups (e.g., healthcare providers, users, and policy makers).	14-17
Limitations	25	Discuss limitations at study and outcome level (e.g., risk of bias), and at review-level (e.g., incomplete retrieval of identified research, reporting bias).	13
Conclusions	26	Provide a general interpretation of the results in the context of other evidence, and implications for future research.	19
**FUNDING**			
Funding	27	Describe sources of funding for the systematic review and other support (e.g., supply of data); role of funders for the systematic review.	1

From: Moher D, Liberati A, Tetzlaff J, Altman DG, The PRISMA Group (2009). Preferred Reporting Items for Systematic Reviews and Meta-Analyses: The PRISMA Statement. PLoS Med 6(6): e1000097. doi:10.1371/journal.pmed1000097

For more information, visit: **www.prisma-statement.org**.

For peer review only - http://bmjopen.bmj.com/site/about/guidelines.xhtml

BMJ Open

Surgical Intervention for Paediatric Infusion-Related Extravasation Injury: A Systematic Review

Journal:	*BMJ Open*
Manuscript ID	bmjopen-2019-034950.R2
Article Type:	Original research
Date Submitted by the Author:	21-Apr-2020

Complete List of Authors:	Little, Max; Whittington Hospital NHS Trust, General Surgery<br>Dupré, Sophie; University of Oxford Nuffield Department of Orthopaedics Rheumatology and Musculoskeletal Sciences<br>Wormald, Justin; University of Oxford Nuffield Department of Orthopaedics Rheumatology and Musculoskeletal Sciences; Stoke Mandeville Hospital, Plastic & Reconstructive Surgery<br>Gardiner, Matthew; University of Oxford Nuffield Department of Orthopaedics Rheumatology and Musculoskeletal Sciences; Wexham Park Hospital, Plastic & Reconstructive Surgery<br>Gale, Chris; Imperial College London, Neonatal Medicine, School of Public Health<br>Jain, Abhilash; University of Oxford Nuffield Department of Orthopaedics Rheumatology and Musculoskeletal Sciences, Plastic Surgery; Imperial College Healthcare NHS Trust, Plastic & Reconstructive Surgery

**Primary Subject Heading**:	Surgery
Secondary Subject Heading:	Paediatrics, Evidence based practice, Intensive care
Keywords:	Paediatric plastic & reconstructive surgery < PAEDIATRIC SURGERY, PLASTIC & RECONSTRUCTIVE SURGERY, Paediatric intensive & critical care < PAEDIATRICS, Neonatal intensive & critical care < INTENSIVE & CRITICAL CARE

SCHOLARONE™
Manuscripts

I, the Submitting Author has the right to grant and does grant on behalf of all authors of the Work (as defined in the below author licence), an exclusive licence and/or a non-exclusive licence for contributions from authors who are: i) UK Crown employees; ii) where BMJ has agreed a CC-BY licence shall apply, and/or iii) in accordance with the terms applicable for US Federal Government officers or employees acting as part of their official duties; on a worldwide, perpetual, irrevocable, royalty-free basis to BMJ Publishing Group Ltd ("BMJ") its licensees and where the relevant Journal is co-owned by BMJ to the co-owners of the Journal, to publish the Work in this journal and any other BMJ products and to exploit all rights, as set out in our licence.

The Submitting Author accepts and understands that any supply made under these terms is made by BMJ to the Submitting Author unless you are acting as an employee on behalf of your employer or a postgraduate student of an affiliated institution which is paying any applicable article publishing charge ("APC") for Open Access articles. Where the Submitting Author wishes to make the Work available on an Open Access basis (and intends to pay the relevant APC), the terms of reuse of such Open Access shall be governed by a Creative Commons licence – details of these licences and which Creative Commons licence will apply to this Work are set out in our licence referred to above.

Other than as permitted in any relevant BMJ Author's Self Archiving Policies, I confirm this Work has not been accepted for publication elsewhere, is not being considered for publication elsewhere and does not duplicate material already published. I confirm all authors consent to publication of this Work and authorise the granting of this licence.

For peer review only

Surgical Intervention for Paediatric Infusion-Related Extravasation Injury: A Systematic Review

Little, Max[1]; Dupré, Sophie[2]; Wormald, Justin[3]; Gardiner, Matthew[4]; Gale, Chris[5]; Jain, Abhilash[2,6]

1. Department of Surgery, The Whittington Hospital, Magdala Avenue, London, UK, N19 5NF
2. Nuffield Department of Orthopaedics, Rheumatology and Musculoskeletal Sciences, University of Oxford, Oxford, UK, OX1 2DJ
3. Department of Plastic Surgery, Stoke Mandeville Hospital, Aylesbury, Buckinghamshire, UK, HP21 8AL
4. Department of Plastic Surgery, Wexham Park Hospital, Wexham St, Slough, UK, SL2 4HL
5. Neonatal Medicine, Department of Medicine, Imperial College London, Chelsea and Westminster Campus, London, UK, SW10 9NH
6. Department of Plastic and Reconstructive Surgery, Imperial College Healthcare NHS Trust, St Mary's Hospital, Praed St, London, UK, W2 1NY

Mr Max Little MBBS MRCS
Core Surgical Trainee, The Whittington Hospital NHS Trust, London, UK

Dr Sophie Dupré MBBS BSc (Hons)
Academic Foundation Doctor, University of Oxford, UK

Mr Justin C R Wormald MBBS MRes MRCS
NIHR Academic Clinical Fellow and Specialist Registrar in Plastic and Reconstructive Surgery, University of Oxford, UK

Mr Matthew Gardiner MA PhD FRCS (Plast)
Hand Fellow, NIHR Senior Clinical Lecturer and Specialist Registrar in Plastic and Reconstructive Surgery, University of Oxford, UK

Dr Chris Gale MBBS MSc PhD MRCPCH *
Clinical Senior Lecturer in Neonatal Medicine and Consultant Neonatologist, Imperial College London, UK

Professor Abhilash Jain MBBS MRCS MSc PhD FRCS (Plast) *
Associate Professor of Plastic and Reconstructive Surgery, University of Oxford and Imperial College NHS Trust, London, UK
*Co-Senior Authors

Corresponding author:
Dr Chris Gale
christopher.gale@imperial.ac.uk
+44 (0)20 3315 3519
Academic Neonatal Medicine, H4.4
Chelsea and Westminster Hospital
Chelsea and Westminster Campus

Running Head: Paediatric extravasation injury

Keywords: Extravasation; Infiltration; Paediatric; Necrosis; Gault

Word Count: 3,980 (including abstract and summary)

For peer review only

Abstract

Objectives

This systematic review aims to assess the quality of literature supporting surgical interventions for paediatric extravasation injury and to determine whether there is sufficient evidence to support invasive techniques in children.

Methods

We performed a systematic review by searching Ovid MEDLINE and EMBASE as well as AMED, CINAHL, Cochrane Central Register of Controlled Trials (CENTRAL), Cochrane Database of Systematic Reviews and clinicaltrials.gov from inception to February 2019. Studies other than case reports were eligible for inclusion if the population was younger than 18 years old, there was a surgical intervention aimed at treating extravasation injury and they reported on outcomes. Study quality was graded according to the National Institutes of Health (NIH) study quality assessment tools.

Results

26 studies involving 728 children were included – one before-and-after study and 25 case series. Extravasation injuries were mainly confined to skin and subcutaneous tissues but severe complications were also encountered, including amputation (one toe and one below elbow). Of the surgical treatments described, the technique of multiple puncture wounds and instillation of saline and/or hyaluronidase was the most commonly used. However, there were no studies in which its effectiveness was tested against another treatment or a control and details of functional and aesthetic outcomes were generally lacking.

Conclusion

Surgical management is commonly reported in the literature in cases where there is significant soft tissue injury but as there are no comparative studies, it is unclear whether this is optimal. Further observational and experimental research evaluating extravasation injuries, including a centralized extravasation register using a universal grading scheme and core outcome set with adequate follow-up, are required to provide evidence to guide clinician decision-making.

For peer review only

Strengths and Limitations

- A systematic review was performed in accordance with the Preferred Reporting Items for Systematic Reviews and Meta-Analyses (PRISMA) statement and using methodology outlined in the Cochrane Handbook for Systematic Reviews of Interventions. It was registered on PROSPERO.
- Two authors used a bespoke inclusion/exclusion form to independently assess study eligibility
- Studies were eligible for inclusion if the population was younger than 18 years old, if there was a surgical intervention aimed at treating extravasation injury in any setting and if they reported on short- or long-term outcomes
- Two researchers also independently assessed the included studies' risk of methodological bias using the National Institutes of Health (NIH) study quality assessment tools
- 18 years old may represent a relatively arbitrary cut-off age to differentiate between 'paediatric' and 'adult' in terms of extravasation injury

For peer review only

Introduction

Extravasation is the inadvertent leakage of a vesicant solution from its intended vascular pathway, commonly a peripheral or central vein, into the surrounding tissue with the potential to harm a patient. A vesicant is any medicine or fluid with the potential to cause blisters, severe tissue injury or necrosis if there is leakage. [1] If left untreated, extravasations may result in delayed healing, scarring and functional morbidity, including amputation in severe cases. Children requiring intravenous (IV) therapy often have multiple risk factors for extravasation injuries, and neonates are at even greater risk of more serious injury due to poor venous integrity, capillary leakage, and expandable subcutaneous tissue that accommodates relatively large volumes of fluid. [2] The reported incidence in adult and paediatric inpatients ranges between 0.1% - 6.5% across studies from the UK, USA and Canada, but inconsistent reporting and documentation means that the true figure is likely to be higher. [2–4]

Prevention of extravasation is the foremost priority. However, when injury does occur, multiple treatments have been described. The “Gault technique” [5] utilizes multiple small incisions made around the injury site. These are subsequently used to instill saline and/or hyaluronidase to wash out the extravasation fluid. This method remains a commonly described technique for the treatment of more severe paediatric extravasations. However, there is a lack of high quality comparative studies in which treatments are evaluated against each other and/or controls. It is therefore not known which of these treatments are effective, harmful or at the very least, necessary; a conclusion supported by a recent scoping review of interventions. [6] That review highlighted the fact that the lack of consensus extended to published guidelines. Their survey also showed wide variation in the frequency of use of the saline washout techniques in neonatal units across the National Health Service (NHS) in the UK. This systematic review aims to evaluate the quality of the literature and to summarize the published outcome data of the surgical management of paediatric extravasation injuries.

For peer review only - http://bmjopen.bmj.com/site/about/guidelines.xhtml

Methods

Our aim was to assess the quality of literature supporting surgical interventions for paediatric extravasation injury and to determine whether there is sufficient evidence to support invasive techniques in children. We performed a systematic review of original clinical studies or systematic reviews (excluding case reports) of paediatric extravasation injury, reported in accordance with the PRISMA [7] statement and using methodology outlined in the Cochrane Handbook for Systematic Reviews of Interventions [8] where applicable. The protocol was developed prospectively, locally peer-reviewed and registered on the PROSPERO database (CRD42016045647). Variability in baseline data, intervention data and outcome data precluded formal meta-analysis. Results are therefore presented as a narrative comparison of different invasive techniques with summary statistics.

Search methods

Studies were identified through a systematic literature search in Ovid MEDLINE and Ovid EMBASE as well as AMED, CINAHL, Cochrane Central Register of Controlled Trials (CENTRAL), Cochrane Database of Systematic Reviews and clinicaltrials.gov from inception to February 2019. To identify grey literature, Web of Science was searched using bespoke searches. Internet-wide searches using Google were employed to identify clinical guidelines describing the management of extravasation in the UK. All of these strategies comprised modifications of a common set of terms to identify the relevant clinical area. Key search terms in this common set included the Medical Subject Headings (MeSH) “extravasation of diagnostic and therapeutic materials", “infant”, “child”, and “preschool”. These MeSH terms were combined with appropriate Boolean operators [Supplementary 1]. The index search terms were then combined with free text terms to identify appropriate clinical studies. No language restrictions were imposed. The search results were merged and, after screening, duplicate citations were discarded along with studies unrelated to the research objective.

For peer review only - http://bmjopen.bmj.com/site/about/guidelines.xhtml

Criteria for selecting studies

Study selection criteria were determined in advance during the protocol stage. Two authors used a bespoke inclusion/exclusion form to independently assess eligibility of the title and abstracts of the search results. [Supplementary 2] Randomized Control Trials (RCTs), quasi-experimental and cross-sectional studies and case-series were eligible for inclusion if the population was younger than 18 years old, if there was a surgical intervention aimed at treating extravasation injury in any setting and if they reported on short- or long-term outcomes. Hyaluronidase injections were considered to be a surgical intervention for the purposes of this review firstly, because they are an invasive procedure and secondly, to allow clinically useful comparison to the combination of Gault's technique with hyaluronidase. Any disparities regarding inclusion of articles were discussed between the authors and a joint decision was made based on the inclusion criteria.

Data analysis

Data extraction was performed by two authors independently and is displayed in Tables 1-5. Descriptive analysis for each group of studies was performed, including the number of children with skin necrosis at presentation as well as the difference in outcomes between those who received an intervention and those who received no intervention.

Study quality assessment

Two researchers also independently assessed the quality of the included studies using the National Institutes of Health (NIH) study quality assessment tools [9] for "case series" and for "before-after studies with no control group" – the two types of included studies. Discrepancies were resolved by discussions with the other authors.

In our review, for a case series to achieve a rating of 'good', it must include a clearly defined study population with comparable subjects as well as clearly described interventions, outcome measures and results with adequate follow-up (questions 2, 4, 5, 6, 7 and 9 in Table 6). If the

For peer review only - http://bmjopen.bmj.com/site/about/guidelines.xhtml

study lacked one of these criteria, it was rated as ‘fair’ but if it lacked two or more, it was deemed ‘poor’ and at high risk of methodological bias. A similar scoring system was used for the one before-and-after study, incorporating the different questions in that tool.

Patient and Public Involvement

No patients involved

For peer review only

For peer review only - http://bmjopen.bmj.com/site/about/guidelines.xhtml

Results

Search strategy

Our search strategy identified a total of 1,966 research articles of which 220 were potentially relevant to the research question. Of the 220 papers 185 did not meet the inclusion criteria. Thirty-five papers were deemed eligible for inclusion after studies with conservative management or no interventions were excluded. The requisite data were immediately available in 26 study reports [4,10–34] and the authors of the remaining nine studies [5,35–42] were contacted twice with additional requests, mainly for exclusively paediatric extravasation data, and given at least two months to reply. Unfortunately, none of the authors were able to provide these data and those studies were excluded. The data were extracted from the remaining 26 studies [Supplementary PRISMA flow-diagram] using a pre-specified (review-specific) proforma [Supplementary 3] by two researchers, with discrepancies resolved by a third.

Study Characteristics

The included studies were published between 1975 and 2018. The mean sample size was 32 with a range of 4-147 participants. One study was a before-and-after comparative study, [24] two were prospective case series, [11,16] and the remaining 23 were retrospective case series. The follow-up periods were not reported in 14 included studies. In the other studies, the follow-up periods ranged from three days to 15 months [Table 1].

For peer review only

For peer review only - http://bmjopen.bmj.com/site/about/guidelines.xhtml

Table 1 – Included studies

Rf	Author	Year	Country	Title	n	Methods	Period	Mean follow-up
4	Murphy	2017	Australia	Extravasation injury in a paediatric population	43	Retrospective Series	-	-
10	Falcone	1989	USA	Nonoperative management of full-thickness intravenous extravasation injuries in premature neonates using enzymatic debridement	15	Retrospective Series	-	6 months
11	Ghanem	2015	UK	Childhood extravasation injuries: improved outcome following the introduction of hospital-wide guidelines	48	Prospective Series	1 year	-
12	Kostogloudis	2015	Greece	Severe Extravasation Injuries in Neonates: A Report of 34 Cases	34	Retrospective Series	2 years	15 months
13	Sung	2016	Korea	Nonoperative Management of Extravasation Injuries Associated With Neonatal Parenteral Nutrition Using Multiple Punctures and a Hydrocolloid Dressing.	12	Retrospective Series	4 years	10 months
14	Odom	2018	USA	Peripheral Infiltration and Extravasation Injury Methodology: A Retrospective Study.	147	Retrospective Series	-	-
15	Compaña	2017	Spain	Lesions Associated With Calcium Gluconate Extravasation	4	Retrospective Series	-	2 months
16	Harris	2001	UK	Limiting the damage of iatrogenic extravasation injury in neonates	56	Prospective Series	3 years	-
17	Andrés	2006	Spain	Treatment protocol for extravasation lesions	15	Retrospective Series	6 years	-
18<br>19	Linder	1983<br>1985	USA	Management of extensive doxorubicin hydrochloride extravasation injuries<br>Prevention of extravasation injuries secondary to doxorubicin	18	Retrospective Series	-	3 months
20	Upton	1979	USA	Major intravenous extravasation injuries	7	Retrospective Series	10 years	-
21	Casanova	2001	France	Emergency treatment of accidental infusion leakage in the newborn: Report of 14 cases	14	Retrospective Series	-	-
22	von Heimburg	1998	Germany	Early and late treatment of iatrogenic injection damage	5	Retrospective Series	-	-
23	Weiss	1975	Israel	Localized necrosis of scalp in neonates due to calcium gluconate infusions: a cautionary note	4	Retrospective Series	-	-
24	Hanrahan	2013	USA	Hyaluronidase for treatment of intravenous extravasations: implementation of an evidence-based guideline in a pediatric population	113	Before-and-after study	4 years	-
25	Boyar	2018	USA	Efficacy of Dehydrated Human Amniotic Membrane Allograft for the Treatment of Severe Extravasation Injuries in Preterm Neonates	4	Retrospective Series	-	1-2 months
26	Yan	2017	China	Incidence, risk factors and treatment outcomes of drug extravasation in pediatric patients in China	18	Retrospective Series	6 months	1– 5 months
27	Myers	2018	USA	Managing intravenous infiltration injuries in the neonatal intensive care unit	28	Retrospective Series	7 years	-
28	Cochran	2002	USA	Treatment of iodinated contrast material extravasation with hyaluronidase	8	Retrospective Series	7 years	-
29	Cho	2007	Korea	Successful combined treatment with total parenteral nutrition fluid extravasation injuries in preterm infants	5	Retrospective Series	4 months	-
30	Onesti	2012	Italy	The use of hyalomatrix PA in the treatment of extravasation affecting premature neonates	26	Retrospective Series	6 years	14 months
31	Firat	2013	Turkey	Management of extravasation injuries: a retrospective study	13	Retrospective Series	2 years	-
32	Ching	2014	UK	Paediatric extravasation injuries: a review of 69 consecutive patients	69	Retrospective Series	1 year	3 days
33	Sivrioglu	2014	Turkey	Versajet hydrosurgery system in the debridement of skin necrosis after Ca gluconate extravasation: report of 9 infantile cases	9	Cohort Study	-	1 year
34	Yan	2014	China	Treatment of cutaneous injuries of neonates induced by drug extravasation with hyaluronidase and hirudoid	13	Retrospective Series	2 years	3 months

For peer review only - http://bmjopen.bmj.com/site/about/guidelines.xhtml

Study quality assessment results

The one before-and-after study [24] was given an overall 'fair' rating by both authors as it had clearly described objectives, interventions and outcome measures with a large sample size but lacked details on participant eligibility and individual level data. Based on the criteria set out above in the study quality assessment, 11 of the 25 included case series – and the Gault study itself – were rated 'poor', six were 'fair' and eight 'good'. [Table 2]

For peer review only

For peer review only - http://bmjopen.bmj.com/site/about/guidelines.xhtml

Table 2 – Quality assessment for included studies

Case series

Ref	Study	Questions 1	2	3	4	5	6	7	8	9	Overall rating	Level of evidence
4	Murphy	Y	Y	N	Y	Y	Y	Y	–	Y	Good	4
12	Kostogloudis	Y	Y	N	Y	Y	Y	Y	–	Y	Good	4
18	Linder 1983	N	Y	Y	Y	Y	Y	Y	–	Y	Good	4
19	Linder 1985	N	Y	Y	Y	Y	Y	Y	–	Y	Good	4
25	Boyar	Y	Y	N	Y	Y	Y	Y	–	Y	Good	4
27	Myers	Y	Y	Y	Y	Y	Y	Y	–	Y	Good	4
30	Onesti	N	Y	N	Y	Y	Y	Y	–	Y	Good	4
34	Yan 2014	Y	Y	N	Y	Y	Y	Y	–	Y	Good	4
10	Falcone	Y	Y	N	Y	Y	N	Y	–	Y	Fair	4
13	Sung	Y	Y	N	Y	Y	N	Y	–	Y	Fair	4
23	Weiss	N	Y	Y	Y	Y	N	Y	–	Y	Fair	4
29	Cho	Y	Y	N	Y	Y	N	Y	–	Y	Fair	4
31	Firat	Y	Y	N	Y	Y	N	Y	–	Y	Fair	4
33	Sivrioglu	Y	Y	N	Y	Y	N	Y	–	Y	Fair	4
11	Ghanem	Y	Y	N	Y	N	N	Y	–	Y	Poor	4
14	Odom	Y	Y	N	Y	N	Y	N	–	Y	Poor	4
15	Compaña	N	N	N	Y	N	N	–	–	Y	Poor	4
16	Harris	N	N	Y	–	Y	N	–	–	Y	Poor	4
17	Andrés	Y	N	Y	N	Y	Y	–	–	Y	Poor	4
20	Upton	Y	N	Y	Y	Y	N	Y	–	Y	Poor	4
21	Casanova	Y	Y	N	Y	Y	N	N	–	Y	Poor	4
22	von Heimburg	N	N	Y	N	Y	N	Y	–	Y	Poor	4
26	Yan 2017	Y	N	Y	–	Y	N	Y	Y	Y	Poor	4
28	Cochran	Y	N	N	Y	Y	N	N	–	N	Poor	4
32	Ching	Y	Y	Y	Y	Y	N	N	–	Y	Poor	4
5	Gault	N	N	Y	N	Y	Y	N	–	Y	Poor	4

Before-and-after study

Ref	Study	1	2	3	4	5	6	7	8	9	10	11	12	Overall rating	Level of evidence
24	Hanrahan	Y	N	–	–	Y	Y	Y	N	–	Y	N	N	Fair	3

Key	Y	N	–
	Yes	No	N/A, Not Recorded, or Cannot Determine

Case series questions

1. Was the study question or objective clearly stated?
2. Was the study population clearly and fully described, including a case definition?
3. Were the cases consecutive?
4. Were the subjects comparable?
5. Was the intervention clearly described?
6. Were the outcome measures clearly defined, valid, reliable, and implemented consistently across all study participants?
7. Was the length of follow-up adequate?
8. Were the statistical methods well described?
9. Were the results well described?

Before-and-after study questions

1. Was the study question or objective clearly stated?
2. Were eligibility/selection criteria for the study population prespecified and clearly described?
3. Were the participants in the study representative of those who would be eligible for the test/service/intervention in the general or clinical population of interest?
4. Were all eligible participants that met the prespecified entry criteria enrolled?
5. Was the sample size sufficiently large to provide confidence in the findings?
6. Was the test/service/intervention clearly described and delivered consistently across the study population?
7. Were the outcome measures prespecified, clearly defined, valid, reliable, and assessed consistently across all study participants?
8. Were the people assessing the outcomes blinded to the participants' exposures/interventions?
9. Was the loss to follow-up after baseline 20% or less? Were those lost to follow-up accounted for in the analysis?
10. Did the statistical methods examine changes in outcome measures from before to after the intervention? Were statistical tests done that provided p values for the pre-to-post changes?
11. Were outcome measures of interest taken multiple times before the intervention and multiple times after the intervention (i.e., did they use an interrupted time-series design)?
12. If the intervention was conducted at a group level (e.g., a whole hospital, a community, etc.) did the statistical analysis take into account the use of individual-level data to determine effects at the group level?

For peer review only

For peer review only - http://bmjopen.bmj.com/site/about/guidelines.xhtml

Patient Characteristics

Across the 26 included studies, there were 728 children. The median age of the 68 children for whom the individual data were available was 0.66 months (interquartile range (IQR) = 5.05). The mean age of 14 months (range: neonatal to 17 years old) was calculated using the data for 322 children across 16 included studies. There was a small difference in sex, with 187 males (57%) and 142 females (43%) (data on sex was lacking for 399 children). 19 of the 26 included studies recorded the sites of the implicated cannula for 553 children. The most common site was the upper limb (n=343; 62%), followed by the lower limb (n=177; 32%) and then the scalp (n=21; 4%). Ten studies specifically stated whether the cannula was a peripheral (n=314; 98%) or central line (n=7; 2%). [Table 3]

For peer review only

Table 3 – Demographics

Ref	Author	n	Mean age (months)	Male	Female	N upper limb	N lower limb	N Scalp	N other	Peripheral Cannula	Central Cannula
**Gault Technique Studies**											
4	Murphy	43	-	-	-	32	9	-	-	-	-
11	Ghanem	48	38.4	-	-	48	25	4	5	73	7
12	Kostogloudis	34	0.6	-	-	6	28	-	-	34	-
17	Andrés	15	36	-	-	14	-	1	-	15	-
21	Casanova	14	1	-	-	4	9	1	-	-	-
32	Ching	69	0.7	32	37	45	17	-	7	-	-
16	Harris	56	-	-	-	-	-	-	-	-	-
	**Total**	**279**	**15.3 (mean)**	**32**	**37**	**149**	**88**	**6**	**12**	**122**	**7**
**Debridement + further surgery**											
10	Falcone	15	0.6	7	8	12	2	1	-	-	-
13	Sung	12	-	6	6	7	5	-	-	-	-
15	Compaña	4	2.6	3	1	2	2	-	-	4	-
17	Andrés (2)	15	36	-	-	14	-	1	-	15	-
20	Upton	7	67.2	-	-	-	-	-	-	-	-
23	Weiss	4	-	-	-	-	-	4	-	4	-
25	Boyar	4	1.1	3	1	3	1	-	-	4	-
29	Cho	5	0.6	-	-	4	-	-	-	6	-
30	Onesti	26	0.6	17	9	14	10	2	-	-	-
31	Firat	13	50	3	10	6	4	3	-	-	-
33	Sivrioglu	9	0.9	-	-	5	3	1	-	9	-
18 19	Linder	18	-	-	-	-	-	-	-	-	-
22	Von Heimburg	5	-	-	-	-	-	-	-	-	-
	**Total**	**137**	**17.7 (mean)**	**46**	**28**	**67**	**27**	**12**	**0**	**42**	**0**
**Hyaluronidase injections**											
14	Odom	147	-	87	60	106	40	2	-	147	-
26	Yan (2017)	18	39.7	10	8	12	6	-	-	18	-
27	Myers	28	1.3	-	-	14	13	1	-	-	-
28	Cochran	8	-	4	4	-	-	-	-	-	-
34	Yan (2014)	13	0.9	8	5	9	3	1	-	-	-
24	Hanrahan	113	-	-	-	-	-	-	-	-	-
	**Total**	**327**	**14.0 (mean)**	**109**	**77**	**141**	**62**	**4**	**0**	**165**	**0**
	**Overall Totals**	**728**	**15.13 (mean)**	**187**	**142**	**343**	**177**	**21**	**12**	**314**	**7**

For peer review only

Of the 16 studies that provided data on comorbidities for 167 children, prematurity was the most common comorbidity in 11 (n=121; 72%). Malignancy (n=25; 15%) was the main comorbidity in three studies.

Of the 20 studies that reported on them, the most commonly extravasated substances affecting 363 children were [Table 4]:

1) Total Parenteral Nutrition (TPN) – 122 children (34%); most common in eight studies.
2) Intravenous maintenance fluids other than those specifically listed – 70 children (19%); most common in three studies. Unfortunately the data was not available for the outcomes of different types of maintenance fluids so they have been grouped together.
3) Calcium-containing products – 32 children (9%); most common in four studies.
4) Dextrose of concentration ≥5% - 28 children (8%); most common in one study.
5) Intravenous antimicrobials – 20 children (6%); most common in no studies. Of those 20 extravasations, five were due to Flucloxacillin – with one injury taking 60 days – to heal and five were due to unspecified cephalosporin antibiotics, three of which required skin grafting. One case of severe contractures and extensor loss on the dorsum of a child's hand was due to an unspecified tetracycline antibiotic. There were no individual outcomes recorded for the seven extravasations of Aciclovir, the single extravasations of Gentamicin, Ganciclovir or the remaining extravasations involving Flucloxacillin and cephalosporin antibiotics.

For peer review only

Table 4 – Comorbidities and extravasated materials in those papers that recorded them

		Comorbidities			Vesicant Type								
Ref	Author	Prematurity	Sepsis	Malignancy	TPN	≥5% Dextrose	Other IV Fluids	Calcium-containing	Antimicrobials	Chemo	Contrast	Dopamine	Other
**Gault Technique Studies**													
4	Murphy	-	-	-	10	11	6	2	6	-	2	-	5
11	Ghanem	14	-	-	22	10	6	-	8	-	-	1	1
12	Kostogloudis	34	-	-	28	4	-	-	2	-	-	-	-
17	Andrés	-	1	5	7	-	-	4	-	4	-	-	-
21	Casanova	-	-	-	-	-	-	2	-	-	-	9	3
32	Ching	"Majority"	-	-	16	-	22	-	-	-	-	-	31
	**Total**	**48**	**1**	**5**	**83**	**25**	**34**	**8**	**16**	**4**	**2**	**10**	**40**
**Debridement + further surgery**													
10	Falcone	15	-	-	9	1	3	1	-	-	-	-	2
13	Sung	9	-	-	12	-	-	-	-	-	-	-	-
15	Compaña	2	-	-	-	-	-	4	-	-	-	-	-
17	Andrés (2)	-	1	5	7	-	-	4	-	4	-	-	-
18 19	Linder	-	-	18	-	-	-	-	-	18	-	-	-
20	Upton	-	-	1	-	1	2	1	1	2	-	-	-
23	Weiss	4	-	-	-	-	-	4	-	-	-	-	-
25	Boyar	4	-	-	4	-	-	-	-	-	-	-	-
29	Cho	5	-	-	4	-	-	-	-	-	-	-	1
30	Onesti	26	-	-	-	-	26	-	-	-	-	-	-
31	Firat	-	1	1	-	1	4	3	3	1	-	-	1
33	Sivrioglu	3	-	-	-	-	-	9	-	-	-	-	-
	**Total**	**68**	**2**	**25**	**36**	**3**	**35**	**26**	**4**	**25**	**0**	**0**	**4**
**Hyaluronidase injections**													
26	Yan (2017)	-	-	-	1	-	-	1	-	-	-	-	-
28	Cochran	-	-	-	-	-	-	-	-	-	8	-	-
34	Yan (2014)	5	-	-	9	-	1	1	-	-	-	-	2
	**Total**	**5**	**0**	**0**	**10**	**0**	**1**	**2**	**0**	**0**	**8**	**0**	**2**
	**Overall Totals**	**121**	**2**	**25**	**122**	**28**	**70**	**32**	**20**	**25**	**10**	**10**	**46**

For peer review only

Interventions

Uncertainty remains over whether there is a difference in the outcomes of children who are treated conservatively or surgically and the case series that exist on conservative management cover a number of treatments for a range of injuries from swelling to full-thickness defects. However, a recent scoping review found that the outcomes used and results detailed were generally limited, [6] making it difficult for clinicians to rely upon those studies in deciding who to treat conservatively. This is why we have focused solely on studies describing invasive interventions: the Gault technique, debridement and further surgery, and hyaluronidase injections. We have still reported on the outcomes of children within each group of studies who received no treatment in order to provide a clinically useful comparison to each intervention.

<u>The Gault technique</u>

279 children with an average age of 15.3 months were involved in the seven studies looking at the Gault technique. No patients had skin necrosis at presentation.

107 were treated using the Gault technique with saline, of which nine (8%) developed full thickness skin injury / skin necrosis, one (1%) had compartment syndrome at presentation requiring fasciotomies and one (1%) had distal foot ischaemia which resolved after treatment. Of those with necrosis, six required no surgical intervention and three were debrided and treated with artificial dermis.

76 were treated using the Gault technique with hyaluronidase, of which three (4%) developed skin necrosis and were all managed conservatively.

One child received only hyaluronidase injections and developed necrosis, which healed spontaneously.

95 children received no treatment, of which ten (11%) developed necrosis (one of whom went on to lose three toes), three (3%) developed an associated infection, one (1%) had an ischaemic toe requiring amputation and one (1%) developed calcinosis cutis. [Table 5]

For peer review only - http://bmjopen.bmj.com/site/about/guidelines.xhtml

Table 5 – Interventions

Ref	Author	Intervention Summary	Outcomes
**Gault Technique Studies**			
4	Murphy	Gault's + saline (11% of children)	Three children suffered injuries, which led to significant tissue necrosis, delayed healing and prolonged morbidity. None of these were washed out due to delayed referral
12	Kostogloudis	Gault's + saline (100% of children)	Seven children developed superficial blistering and epidermolysis, while six developed necrosis, all post-treatment. All wounds healed within 25 days. One case of distal foot ischaemia resolved after treatment
16	Harris	Gault's + saline (100% of children)	No episodes of skin or soft tissue loss were recorded and no reconstructive surgery was required
17	Andrés (early)	Gault's + saline (67% of children)	Seven of ten treated with Gault's technique avoided necrosis and recovered fully. Three developed minor necrosis., Tthe remaining five were debrided and received artificial skin and obtained satisfactory outcomes
21	Casanova	Gault's + hyaluronidase (79% of children) / + saline (14%) with liposuction	No skin involvement in ten children; Blistering healed in one; Necrosis resolved in three
11	Ghanem	Gault's + hyaluronidase (46% of children) with liposuction	Three children had tissue necrosis – two were late referrals; unclear if the other one received washout. There was satisfactory healing with no requirement for surgical intervention
32	Ching	Gault's + hyaluronidase (62% of children)	Of the 62% of children washed out, none developed complications. One calcinosis cutis and one ischaemic toe requiring amputation amongst children receiving no treatment
**Debridement + further surgery**			
10	Falcone	Topical fibrinolysin/deoxyribonuclease ointment then debridement	All wounds healed completely with no infections and no functional scar contractions at up to 16 months follow-up. No skin grafts were required
23	Weiss	Wet dressings and repeated economical debridement	Wounds healed well in 15 to 40 days. Scars were visible but without discolouration
31	Firat	Topical hirudin and antibiotics, then 3% boric acid, then repetitive debridement	Seven children required split-thickness skin grafting and two required fasciocutaneous flaps. All recovered well, with scar development in four. Minor functional loss in the hands or feet as a result of scar formation was managed by physiotherapy and pressure garments
30	Onesti	Topical collagenase, then debridement, and then Hyalomatrix PA (dermal substitute)	18 children healed fully after 21 days. Four had pathological scars and four had debilitating scar contractures needing secondary surgery
25	Boyar	Enzymatic or autolytic debridement before mechanical debridement and application of dehydrated human amniotic membrane allograft (dHAMA)	Complete closure of significant wounds with minimal soft scars and normal pigmentation
15	Compaña	Topical steroids, Burow's Solution and Silver Sulfadiazine for all children. 3 (60%) then underwent debridement followed by split-thickness skin grafts	Successful healing in two children. One died of other causes
29	Cho	Topical antibiotic + anti-inflammatory herbal mixture for all children. 1 (20%) debridement. 1 (20%) escharotomy	The child who underwent debridement had a small-sized contracture at 50 days
20	Upton	Debridement and skin grafts. Excision of extensor tendons if infected or devascularized. All children required two or more operations	Two children experienced contractures, two had extensor loss, one had hair loss, one had loss of motion and one required further reconstruction
18 19	Linder	Debridement and wound closure: mostly split-thickness skin grafts or delayed primary closure. All children had at least 2 operations	The mean time for wound closure was 49 days (range 10–85 days). Three children died before wound closure. At least one patient needed a split-thickness skin graft. One child developed sympathetic dystrophy syndrome. Some children developed permanent joint stiffness
22	von Heimburg	Debridement, allogeneic donor tissue grafts and autologous split-thickness skin grafts	After 15 days there was full healing in all five infants
17	Andrés (late)	Debridement + dermal substitute in 33%	The five late referrals were debrided and received artificial skin. All obtained satisfactory outcomes
13	Sung	Multiple punctures using a scalpel blade + hydrocolloid dressing. Then debridement	All children showed favourable results without functional deficits or conspicuous scars
33	Sivrioğlu	Versajet hydrosurgery for all children. 1 (11%) sharp debridement	Minimal scar formation with no hypertrophic scarring in any patient
**Hyaluronidase injections**			
14	Odom	Injection of hyaluronidase or Phentolamine without incisions	No children required surgical intervention for wound healing or had an infection
24	Hanrahan	Injection of hyaluronidase	Mean harm scores were similar between the group receiving hyaluronidase and the group not receiving it
26	Yan (2017)	Injection of hyaluronidase (33%). 1 (6%) required surgical excision of a lesion	All healed and had "good outcomes"
27	Myers	Injection of hyaluronidase in 50%	Time to healing averaged 16.2 days (range 1-82 days). No patient required surgical intervention
28	Cochran	Injection of hyaluronidase in 25%	One patient had a prolonged course with swelling and skin peeling of the hand
34	Yan (2014)	Application of hirudoid and injection of hyaluronidase	Three children lost to follow-up. Negligible loss of functional movements. One case of scarring and readmission with calcinosis

For peer review only - http://bmjopen.bmj.com/site/about/guidelines.xhtml

Debridement and further surgery

There were 137 children with an average age of 17.7 months across the 14 studies in the debridement and further surgery group.

Nine studies (98 children) used management plans comprising debridement and further surgery in combination with conservative measures, details of which are shown in Table 4. [10,13,15,17,23,25,29–31] Of those 98 children, 34 (35%) had established full thickness skin injury / skin necrosis at presentation and 28 (29%) developed necrosis after conservative measures. 84 children (86%) underwent mechanical debridement as part of their treatment. The vast majority of children's wounds healed with minimal scarring and no functional deficit but four (4%) had debilitating scar contractures necessitating further surgery, ten (10%) required skin grafts, and two (2%) required fasciocutaneous flaps.

The other five studies (39 children) used only debridement and further surgery. All of those 39 children had full thickness skin injury / skin necrosis at presentation. 30 (77%) underwent mechanical debridement, of whom 22 (56%) required further operations including skin grafts and fasciocutaneous flaps and two developed contractures. The remaining nine children (23%), all from one study, underwent Versajet hydrosurgery with good healing in all children and one requiring sharp debridement.

In total, of the 137 children in the debridement and further surgery group, 103 (75%) developed skin necrosis, all of whom (in addition to some of those who were yet to develop necrosis, total n=123 (89%)) received either mechanical or water jet debridement. At least 38 children (28%) went on to require further operations.

Hyaluronidase injections

The final group of studies used hyaluronidase injections at the extravasation site without the need for incisions or washouts. This group comprised six studies and 327 children. [14,24,26–28,34]

97 children received hyaluronidase injections, one (1%) of whom had skin necrosis at presentation that resolved, and one (1%) developed necrosis requiring further surgery. Of the remaining 230 children that did not receive hyaluronidase, none developed skin necrosis at any stage, although reporting on the extent of skin damage and eventual outcomes across the studies was generally poor.

For peer review only

Comparison of the interventions

The Gault technique

Among the studies included in this review, Ghanem et al [11] provides the closest approximation of a comparative study of the Gault technique in children: 48 extravasations were diagnosed and children were divided into early (<24 hours, n=45) and later (>24 hours, n=3) referrals. Among early referrals, 22 were deemed to be 'at risk' injuries and received washout using Gault's technique. Skin necrosis occurred in one of 45 early referrals but in two of three later referrals. The conclusion of this study, that the incidence of necrosis is higher in the later referral group, is limited by low methodological quality including the use of an arbitrary 24-hour cut-off, the small size of the later referral group and a lack of data describing the types of vesicants. It is important to note that the necrosis resolved with conservative management in all three cases.

Of the 183 children treated with a variation of the Gault technique across the included studies, 12 (7%) developed skin necrosis (none had skin necrosis at presentation). This compares favourably to the 11% (10/95) of children who received no treatment across those same studies. However, 73% (69/95) of the untreated group were late referrals and may be expected to have worse outcomes related to time of presentation. The only study to apply the Gault technique in which plastic surgery review time and patient ages were similar was Ching, Wong and Milroy. [32] In that study, none of the 43 children treated with Gault's technique and hyaluronidase developed any complications whereas there were three cases of associated infection, one ischaemic toe requiring amputation and one case of calcinosis cutis amongst the 26 children that received no treatment.

The degree of injury was only recorded for 36 (13%) of the 279 children in the included studies describing the Gault technique, of which 22 (8%) were full-thickness skin injuries.

Debridement and further surgery

73 children (53%) of the 137 in the debridement and further surgery studies had at least skin necrosis / full thickness skin injury at presentation

For peer review only

In contrast to the Gault as well as the hyaluronidase injection studies, the degree of injury was documented for 93% of the children (128/137).

Hyaluronidase injections

One (0.3%) of the 327 children reported in studies describing hyaluronidase injections had skin necrosis at presentation, and one (0.3%) went on to develop it. The degree of injury was recorded for only 49 (15%) of the 327 children. The reporting of patient outcomes was particularly scarce in this group.

Injuries

The types of injury were recorded for 197 (27%) of the 728 children across the included studies. Injuries were limited to pain, swelling or erythema (n=34; 17%) or partial-thickness skin injury such as superficial blistering in others (n=35; 18%) and full-thickness skin injury such as necrosis in the largest number of children (n=121; 61%). However, these figures are skewed by the fact that 10 studies only looked at extravasation injuries causing at least skin necrosis. [10,12,18–20,22,23,25,31,33] Two studies described toe amputations secondary to ischaemia. [4,32] One study of major injuries with full-thickness tissue loss had several casualties including a below elbow amputation and two episodes of extensor loss. [20]

Table 6 displays only the recorded injuries suffered by children who received an intervention. It also states whether those injuries were pre- or post-intervention or whether the timing was unclear. The overall figures are similar to those above, with injuries recorded for 184 (25%) of the children. Of that 184, 33 (18%) were pain, swelling or erythema, 32 (17%) were partial-thickness and 114 (62%) were full-thickness injuries. One study described a transient episode of foot ischaemia that resolved after washout and one compartment syndrome of the left forearm requiring fasciotomies, with washout through the fasciotomy wounds. [12]

For peer review only - http://bmjopen.bmj.com/site/about/guidelines.xhtml

Table 6 – Injuries to children receiving an intervention in those papers that recorded them

		Skin changes			Other injuries				
**Ref**	**Author**	**Pain/swelling/erythema**	**Partial thickness skin injury**	**Full thickness skin injury**	**Tendon**	**Nerve**	**Vascular**	**Compartment Syndrome**	**Amputation**
**Gault Technique Studies**									
12	Kostogloudis	-	7 (post)	6 (post)	-	-	1 (pre)	1 (pre)	-
17	Andrés (early)	-	-	3 (post)	-	-	-	-	-
21	Casanova	10 (pre)	1 (pre)	3 (post)	-	-	-	-	-
	**Total**	**10**	**8**	**12**	**0**	**0**	**1**	**1**	**0**
**Debridement + further surgery**									
10	Falcone	-	-	16 (pre)	-	-	-	-	-
13	Sung	1 (pre)	2 (pre)	2 (pre) + 7 (post)	-	-	-	-	-
15	Compaña	-	-	3 (pre)	-	-	-	-	-
17	Andrés (late)	-	-	5 (pre)	-	-	-	-	-
18 19	Linder	-	-	18 (pre)	-	-	-	-	-
20	Upton	-	-	7 (?)	2 (?)	-	-	-	1 (?)
22	von Heimburg	-	-	5 (pre)	-	-	-	-	-
23	Weiss	-	-	4 (pre)	-	-	-	-	-
25	Boyar	-	-	4 (pre)	-	-	-	-	
29	Cho	-	-	2 (pre)	-	-	-	-	-
30	Onesti	-	18 (pre)	8 (pre)	-	-	-	-	-
31	Firat	-	-	9 (pre)	-	-	-	-	-
33	Sivrioglu	-	-	9 (pre)	-	-	-	-	-
	**Total**	**1**	**20**	**100**	**2**	**0**	**0**	**0**	**1**
**Hyaluronidase injections**									
28	Cochran	-	1 (post)	-	-	-	-	-	-
26	Yan (2017)	17 (pre)	-	1 (pre)	-	-	-	-	-
34	Yan (2014)	5 (pre)	3 (pre)	1 (pre)	-	-	-	-	-
	**Total**	**22**	**4**	**2**	**0**	**0**	**0**	**0**	**0**
	**Overall Totals**	**33**	**32**	**114**	**2**	**0**	**1**	**1**	**1**

Key

'pre' = pre-intervention

'post' = post-intervention

'?' = unclear whether pre- or post-intervention

For peer review only

Millam's grading system for extravasation injuries

Several of the included studies [12,14,21,26,29,33] make reference to the four stages of extravasation/infiltration severity, which was first published by Millam in 1988. [43] This focuses on presence of pain, erythema, oedema, capillary refill, skin temperature and breakdown and it has been proposed that stages III & IV may require intervention. [44] Of those studies, three state that all of the children had stage III or IV injuries before any intervention, [12,14,29] and two made reference to the grading scheme without using it to assess the injuries. [21,33] In one case series, [26] treatment was determined by the severity of extravasation injury based on a grading system similar to Millam's. Of the 18 children, the seven with Grade I and II injuries were managed conservatively. Ten children had grade III injuries – five were managed conservatively but the other five who had also developed skin erythema received active treatment with hyaluronidase injections. The one child with a grade IV injury required excision of the lesion – the sole surgical intervention in that study – after having been injected with hyaluronidase. All wounds healed with 'good outcomes'.

Other than the presence of necrosis signifying a grade IV injury, the other included studies described injuries in ways that did not easily translate into Millam's grading system. This led to the authors of this study using the injury descriptors seen in the Table 5 headings.

For peer review only

Discussion

This paper describes a systematic review of characteristics and operative management of paediatric extravasation injuries. Our review highlighted three key issues. Firstly, surgical management is commonly reported in the literature in cases where there is significant soft tissue injury but as there are no comparative studies, it is unclear whether this is optimal. Secondly, there are no data to support one surgical management approach over another, or over no treatment. Thirdly, there are no data on the adverse events caused by the different surgical interventions.

A variation of the Gault technique was used in the largest number of children to receive surgical treatment across the included studies. Gault [4] described a retrospective case series of 96 patients, ranging in age from newborn to 70 years, who were split into two groups: those seen within 24 hours of the extravasation and those seen later than this. Patients seen within 24 hours had treatment as per the Gault technique while those seen after 24 hours had no treatment. The original Gault paper was not included in our review because there were no separate paediatric data available but a separate study quality assessment by two authors rated the study as 'poor' due to the lack of a clearly defined objective or population, subjects that were not comparable and an inadequate length of follow-up. [Table 6] Despite the inherent limitations in the original Gault study, the method he described is widely used, as demonstrated by this review where it was a commonly described technique to manage extravasation injuries. This approach is not without downsides: it is an invasive procedure with associated morbidity such as pain and possible tendon and nerve damage, and takes considerable time to perform.

Evidence for the continuing use of the "Gault technique"

The degree of injury was recorded for 13% of the children included in the Gault technique studies, 93% of those in the debridement and further surgery group and 15% of those in the hyaluronidase injection group. These figures demonstrate some of the difficulties faced in drawing comparisons between different

For peer review only - http://bmjopen.bmj.com/site/about/guidelines.xhtml

interventions, both due to the poor recording of injuries and because of the fact that accurate documentation of injury severity appears to be more consistently applied following more severe injuries. To add to this, Table 6 demonstrates the inter-study variability on whether injuries were recorded pre- or post-intervention with two studies even reporting a mixture of both separate to the eventual patient outcomes. [13,21] Even when the data is presented in this way, it does not take into account how soon after the intervention the injury has taken place. For example, in several studies, skin necrosis developed one to two days after the intervention but then resolved soon afterwards. [12,17,21] This highlights the overall lack of clarity in the reporting of injuries and outcomes. It was therefore not possible to perform pooled analysis from included studies of Gault and alternative techniques due to study heterogeneity and incomplete outcome reporting.

All but one of the included studies were case series and the one before-and-after study [24] primarily examined the effect of new extravasation treatment guidelines on knowledge of hyaluronidase, extravasation incident reporting and time from extravasation discovery to treatment. Therefore, even though they found similar harm scores for the hyaluronidase and the non-hyaluronidase groups, the results could not be relied upon to assess the effectiveness of the intervention.

In addition, inclusion criteria varied considerably, details of the injuries were poorly described, and no studies made a comparison to another intervention. Finally, detailed outcome measures following interventions were generally lacking – many studies used subjective descriptions (such as 'healed completely', 'healed fully' or 'no hypertrophic scarring') on which it is difficult to base decisions in clinical practice. In summarizing the included studies, it can be noted that despite widespread use, there is no high-quality evidence to support the Gault approach or its modifications.

The future of extravasation management

A key conclusion from this review is that resolution of extravasation injuries is common regardless of the treatment applied. In the absence of data to support one treatment modality over another, it is imperative that high quality methodology randomized comparisons are undertaken; however, given the high rate of resolution with conservative treatment, any such trial will require a large, collaborative and multidisciplinary approach.

For peer review only - http://bmjopen.bmj.com/site/about/guidelines.xhtml

Although many of the in the included studies required minimal intervention, full-thickness skin injury was commonplace; this has the potential for lifelong scarring in patients with an immature skin barrier such as children and preterm infants in particular. More serious complications were rare, but given that only a minority of studies reported on longer-term outcomes, these may underestimate the true burden of extravasation injuries. We suggest the creation of a core outcome set for extravasation injury treatment and that future studies use longer-term follow-up to monitor those outcomes.

Creation of a centralized extravasation register would permit larger-scale assessment of the available interventions and the use of a universal grading scheme (such as Millam's [43]) to assess the initial extent of injuries would also aid in their comparison. However, this requires meticulous documentation of the appearance of the extravasated site as well as the extent of tissue damage.

This review was limited by the low methodological quality of included studies and inconsistent outcome reporting. Variability in baseline data, intervention data and outcome data precluded formal meta-analysis. These limitations prevent the authors from providing definitive clinical recommendations either for or against paediatric extravasation, based on the current evidence.

Conclusion

There is a paucity of evidence to inform surgical treatment of paediatric and neonatal extravasation injuries. The Gault technique is a commonly described approach, but we did not identify any evaluation of its effectiveness compared to other approaches or conservative management. A centralized extravasation register, use of a standardized and agreed grading scheme, development of a core outcome set and adequate follow-up of extravasation injuries are required to provide incidence and outcome data for this condition. This would inform a much-needed, definitive trial of therapeutic approaches to extravasation injuries in children and neonates.

For peer review only

Author Contributions

Max Little was one of the authors to independently assess eligibility of the title and abstracts of the search results. He then aided in extraction and analysis of the relevant data from included studies, performed the study quality assessments and drafted all of the versions of the article leading up to this submission.

Sophie Dupré also aided in the extraction and analysis of the relevant data from included studies, performed the study quality assessments and assisted in the drafting of each version of the article.

Justin Wormald made substantial contributions to the design of the study, aided in the extraction and analysis of the relevant data, as well as reviewing and critically editing each version of the article.

Matthew Gardiner made substantial contributions to the design of the study as well as reviewing and critically editing each version of the article.

Chris Gale acted as one of the co-senior authors, lending his expertise in paediatrics. He reviewed and critically edited the later versions of the article, substantially changing the text and ensuring its accuracy.

Abhilash Jain acted as the other co-senior author, lending his expertise in plastic surgery. He made substantial contributions to the design of the study, aided in the analysis and interpretation of the data from included studies and critically reviewed each version of the article.

Conflict of interest: CG has received support from Chiesi Pharmaceuticals to attend an educational conference; in the past 5 years he been investigator on received research grants from Medical Research Council, National Institute of Health Research, Canadian Institute of Health Research, Department of Health in England, Mason Medical Research Foundation, Westminster Medical School Research Trust and Chiesi Pharmaceuticals; he declares no other conflict of interest.

Funding: JW is an NIHR Academic Clinical Fellow; Chris Gale is funded by a Medical Research Council (MRC) Clinician Scientist Fellowship. This research is supported by the National Institute for Health Research (NIHR) infrastructure at NDORMS. The views expressed are those of the author(s) and not necessarily those

For peer review only

of the NHS, the NIHR or the Department of Health. No funding was directly received for the development of this manuscript.

Data availability: All data are included in the article or uploaded as supplementary information. No additional data are available.

For peer review only

References

1. Doellman D, Hadaway L, Bowe-Geddes LA, et al. Infiltration and extravasation: Update on prevention and management. *J Infus Nurs*. 2009;32(4):203-211. doi:10.1097/NAN.0b013e3181aac042

2. Paquette V, McGloin R, Northway T, DeZorzi P, Singh A, Carr R. Describing intravenous extravasation in children (DIVE study). *Can J Hosp Pharm*. 2011;64(5):340-345.

3. Wilkins C, Emmerson A. Extravasation injuries on regional neonatal units. *Arch Dis Child Fetal neonatal Ed*. 2004;89(3).

4. Murphy A, Gilmour R, Coombs C. Extravasation injury in a paediatric population. *ANZ J Surg*. 2017.

5. Gault D. Extravasation injuries. *Br J Plast Surg*. 1993;46(2):91.

6. Corbett M, Marshall D, Harden M, Sam O, Phillips R, McGuire W. Treatment of extravasation injuries in infants and young children: a scoping review and survey. Southampton (UK): NIHR Journals Library. *Health Technol Assess (Rockv)*. 2018;22(46). doi:10.3310/hta22460

7. Moher D, Liberati A, Tetziaff J, Altman D. Preferred Reporting Items for Systematic Reviews and Meta-Analyses. The PRISMA Statement. *PLoS Med*. 2009;6(7):e1000097. doi:10.1371/journal.pmed1000097

8. Higgins J, Green S. Cochrane Handbook for Systematic Reviews of Interventions Version 5.1.0 [updated March 2011]. *Cochrane Collab*. 2011. http://handbook.cochrane.org.

9. NIH Study Quality Assessment Tools. https://www.nhlbi.nih.gov/health-topics/study-quality-assessment-tools. Accessed March 25, 2019.

10. Falcone P, Barrall D, Jeyarajah D, Grossman J. Nonoperative management of full-thickness intravenous extravasation injuries in premature neonates using enzymatic debridement. *Ann Plast Surg*. 1989;22(2):146.

11. Ghanem M, Mansour A, Exton R, et al. Childhood extravasation injuries: improved outcome following the introduction of hospital-wide guidelines. *J Plast Reconstr Aesthetic Surg*. 2015;68(4):505.

12. Kostogloudis N, Demiri E, Tsimponis A, et al. Severe Extravasation Injuries in Neonates: A Report of 34 Cases. *Pediatr Dermatol*. 2015;32(6):830.

13. Sung K-Y, Lee S-Y. Nonoperative Management of Extravasation Injuries Associated With Neonatal Parenteral Nutrition Using Multiple Punctures and a Hydrocolloid Dressing. *Wounds A Compend Clin Res Pract*. 2016;28(5):145-152.

14. Odom B, Lowe L, Yates C. Peripheral Infiltration and Extravasation Injury Methodology: A Retrospective Study. *J Infus Nurs*. 2018;41(4):247-252.

15. Pacheco Compaña F, Midón Míguez J, de Toro Santos F. Lesions Associated With Calcium Gluconate Extravasation. *Ann Plast Surg*. 2017;79(5):444-449.

For peer review only

16. Harris P, Bradley S, Moss A. Limiting the damage of iatrogenic extravasation injury in neonates. *Plast Reconstr Surg*. 2001;107(3):893-894. doi:https://doi.org/10.1097/00006534-200103000-00053

17. Andrés A, Burgos L, López Gutiérrez J, et al. Treatment protocol for extravasation lesions. *Cir Pediatr*. 2006;19(3):136-139.

18. Linder R, Upton J, Osteen R. Management of extensive doxorubicin hydrochloride extravasation injuries. *J Hand Surg Am*. 1983;8(1):32-38. doi:https://doi.org/10.1016/S0363-5023(83)80048-3

19. Linder R, Upton J. Prevention of extravasation injuries secondary to doxorubicin. *Postgrad Med J*. 1985;77(4):105-9,112,114. doi:https://doi.org/10.1080/00325481.1985.11698920

20. Upton J, Mulliken J, Murray J. Major intravenous extravasation injuries. *Am J Surg*. 1979;137(4):497-506. doi:https://doi.org/10.1016/0002-9610(79)90121-1

21. Casanova D, Bardot J, Magalon G. Emergency treatment of accidental infusion leakage in the newborn: Report of 14 cases. *Br J Plast Surg*. 2001;54(5):396-399.

22. von Heimburg D, Pallua N. Early and late treatment of iatrogenic injection damage. *Chirurg*. 1998;69(12):1378-1382. doi:https://doi.org/10.1007/s001040050588

23. Weiss Y, Ackerman C, Shmilovitz L. Localized necrosis of scalp in neonates due to calcium gluconate infusions: a cautionary note. *Pediatrics*. 1975;56(6):1084-1086.

24. Hanrahan K. Hyaluronidase for treatment of intravenous extravasations: implementation of an evidence-based guideline in a pediatric population. *J Spec Pediatr Nurs*. 2013;18(3):253-262. doi:https://doi.org/10.1111/jspn.12035

25. Boyar V, Galiczewski C. Efficacy of Dehydrated Human Amniotic Membrane Allograft for the Treatment of Severe Extravasation Injuries in Preterm Neonates. *Wounds*. 2018;30(8):224-228.

26. Yan Y, Gong M, Chen J, Li D, Xu T, Zou H. Incidence, risk factors and treatment outcomes of drug extravasation in pediatric patients in China. *Turk J Pediatr*. 2017;(59):162-168.

27. Myers P, Krasniak P, Bell D. Managing intravenous infiltration injuries in the neonatal intensive care unit. *J Burn Care Res*. 2018;39(9):S151.

28. Cochran T, Bomyea K, Kahn M. Treatment of iodinated contrast material extravasation with hyaluronidase. *Acad Radiol*. 2002;9.

29. Cho K, Lee S, Burm J, Park E. Successful combined treatment with total parenteral nutrition fluid extravasation injuries in preterm infants. *J Korean Med Sci*. 2007;22(3):588.

30. Onesti M, Carella S, Maruccia M, Ciotti M, Scuderi N. The use of hyalomatrix PA in the treatment of extravasation affecting premature neonates. *Plast Reconstr Surg*. 2012;129(1):219e-21e.

31. Firat C, Erbatur S, Aytekin A. Management of extravasation injuries: a retrospective study. *J Plast Surg Hand Surg*. 2013;47(1):60.

32. Ching L, Wong K, Milroy C. Paediatric extravasation injuries: a review of 69 consecutive patients. *Int J Surg*. 2014;12(10):1036.

For peer review only - http://bmjopen.bmj.com/site/about/guidelines.xhtml

33. Sivrioğlu N, Irkören S. Versajet hydrosurgery system in the debridement of skin necrosis after calcium gluconate extravasation: report of 9 infantile cases. *Acta Orthop Traumatol Turc*. 2014;48(1):6.

34. Yan Y, Fan Q, Li A, Chen J, Dong F, Gong M. Treatment of cutaneous injuries of neonates induced by drug extravasation with hyaluronidase and hirudoid. *Iran J Pediatr*. 2014;24(4):352-358.

35. Loth T, Eversmann W. Extravasation injuries in the upper extremity. *Clin Orthop Relat Res*. 1991;(272):48.

36. Scuderi N, Onesti M. Antitumor agents: extravasation, management, and surgical treatment. *Ann Plast Surg*. 1994;32(1):39.

37. Langstein N, Duman H, Seelig D, Butler E, Evans R. Retrospective study of the management of chemotherapeutic extravasation injury. *Ann Plast Surg*. 2002;49(4):369.

38. D'Andrea F, Onesti M, Nicoletti G, et al. Surgical treatment of ulcers caused by extravasation of cytotoxic drugs. *Scand J Plast Reconstr Surg Hand Surg*. 2004;38(5):288-292.

39. Rose R, Felix R, Crawford-Sykes A, Venugopal R, Wharfe G, Arscott G. Extravasation injuries. *West Indian Med J*. 2008;57(1):40.

40. Sbitany H, Koltz F, Mays C, Girotto A, Langstein N. CT contrast extravasation in the upper extremity: strategies for management. *Int J Surg*. 2010;8(5):384.

41. Kim S, Cook K, Lee I, Park D. Computed tomography contrast media extravasation: treatment algorithm and immediate treatment by squeezing with multiple slit incisions. *Int Wound J*. 2017;14(2):430-434.

42. Onesti M, Carella S, Fioramonti P, Scuderi N. Chemotherapy Extravasation Management: 21-Year Experience. *Ann Plast Surg*. 2017;79(5):450-457.

43. Millam D. Managing complications of IV therapy. *Nursing (Lond)*. 1988;(18):34-43.

44. Flemmer L, Chan J. A pediatric protocol for management of extravasation injuries. *Pediatr Nurs*. 1993;18:44-47.

For peer review only

Search Results

Table of Contents

Search History .... page 2

1. Thoracic and abdominal extravasation: a complication of hyperalimentation in infants. .... page 4

2. [Traumatic cerebral aneurysm showing remarkable extravasation into the lateral ventricle--report of a case in an infant (author's transl)]. .... page 4

3. Urinary ascites in children owing to extravasation of urine from the bladder. .... page 4

4. Bone scintigraphy for neonatal osteomyelitis: simulation by extravasation of intravenous calcium. .... page 4

5. Perirenal urinary extravasation associated with urethral valves in infants. .... page 4

6. Nafcillin extravasation injury. Use of hyaluronidase as an antidote. .... page 4

7. Cutaneous calcinosis in a neonate following extravasation of calcium gluconate. .... page 4

8. Urinary extravasation in the newborn male with posterior urethral valves. .... page 4

9. Intrathoracic extravasation of sclerosing agents associated with central venous catheters. .... page 5

10. Nonoperative management of full-thickness intravenous extravasation injuries in premature neonates using enzymatic debridement. .... page 5

For peer review only

Search History

1. AMED, EMBASE, Medline, CINAHL; p?ediatric*.ti,ab; 2702402 results.
2. AMED, EMBASE, Medline, CINAHL; neonat*.ti,ab; 508216 results.
3. AMED, EMBASE, Medline, CINAHL; infant*.ti,ab; 761110 results.
4. AMED, EMBASE, Medline, CINAHL; child.ti,ab; 737276 results.
5. AMED, EMBASE, Medline, CINAHL; 1 OR 2 OR 3 OR 4; 4209881 results.
6. AMED, EMBASE, Medline, CINAHL; extra?vasat*.ti,ab; 19189 results.
7. AMED, EMBASE, Medline, CINAHL; extravasation.ti,ab; 30514 results.
8. AMED, EMBASE, Medline, CINAHL; 6 OR 7; 32931 results.
9. AMED, EMBASE, Medline, CINAHL; 5 AND 8; 1849 results.
10. AMED, EMBASE, Medline, CINAHL; outcome*.ti,ab; 2946939 results.
11. AMED, EMBASE, Medline, CINAHL; PROM*.ti,ab; 2921492 results.
12. AMED, EMBASE, Medline, CINAHL; patient?related*.ti,ab; 131 results.
13. AMED, EMBASE, Medline, CINAHL; patient?report*.ti,ab; 689 results.
14. AMED, EMBASE, Medline, CINAHL; 10 OR 11 OR 12 OR 13; 5654365 results.
15. AMED, EMBASE, Medline, CINAHL; conservative*.ti,ab; 231035 results.
16. AMED, EMBASE, Medline, CINAHL; non?operative*.ti,ab; 11082 results.
17. AMED, EMBASE, Medline, CINAHL; non?surg*.ti,ab; 14737 results.
18. AMED, EMBASE, Medline, CINAHL; cold*.ti,ab; 225847 results.
19. AMED, EMBASE, Medline, CINAHL; cool*.ti,ab; 101309 results.
20. AMED, EMBASE, Medline, CINAHL; hot*.ti,ab; 160999 results.
21. AMED, EMBASE, Medline, CINAHL; warm*.ti,ab; 110969 results.
22. AMED, EMBASE, Medline, CINAHL; elevat*.ti,ab; 1359688 results.
23. AMED, EMBASE, Medline, CINAHL; dress*.ti,ab; 53707 results.
24. AMED, EMBASE, Medline, CINAHL; 15 OR 16 OR 17 OR 18 OR 19 OR 20 OR 21 OR 22 OR 23; 2170626 results.
25. AMED, EMBASE, Medline, CINAHL; medic*.ti,ab; 3892625 results.
26. AMED, EMBASE, Medline, CINAHL; steroid*.ti,ab; 449723 results.
27. AMED, EMBASE, Medline, CINAHL; cortico?steroid*.ti,ab; 114994 results.
28. AMED, EMBASE, Medline, CINAHL; corticosteroid*.ti,ab; 206312 results.
29. AMED, EMBASE, Medline, CINAHL; sub?cut*.ti,ab; 245741 results.
30. AMED, EMBASE, Medline, CINAHL; subcut*.ti,ab; 189955 results.
31. AMED, EMBASE, Medline, CINAHL; inject*.ti,ab; 1404121 results.
32. AMED, EMBASE, Medline, CINAHL; topical*.ti,ab; 199318 results.
33. AMED, EMBASE, Medline, CINAHL; cream*.ti,ab; 38332 results.
34. AMED, EMBASE, Medline, CINAHL; ointment*.ti,ab; 24526 results.
35. AMED, EMBASE, Medline, CINAHL; emollient*.ti,ab; 3696 results.
36. AMED, EMBASE, Medline, CINAHL; antidote*.ti,ab; 11910 results.
37. AMED, EMBASE, Medline, CINAHL; Sodium?thiosulfate*.ti,ab; 6 results.
38. AMED, EMBASE, Medline, CINAHL; mechlorethamine*.ti,ab; 1486 results.
39. AMED, EMBASE, Medline, CINAHL; Dexrazoxane*.ti,ab; 1175 results.
40. AMED, EMBASE, Medline, CINAHL; N?acetylcysteine*.ti,ab; 1604942 results.
41. AMED, EMBASE, Medline, CINAHL; vitamin?C*.ti,ab; 422 results.
42. AMED, EMBASE, Medline, CINAHL; ascorbic*.ti,ab; 58559 results.
43. AMED, EMBASE, Medline, CINAHL; basic AND fibroblast AND growth AND factor.ti,ab; 29979 results.
44. AMED, EMBASE, Medline, CINAHL; granulocyte AND macrophage AND colony AND stimulating AND factor.ti,ab; 37389 results.
45. AMED, EMBASE, Medline, CINAHL; hyaluroni*.ti,ab; 42841 results.
46. AMED, EMBASE, Medline, CINAHL; salin*.ti,ab; 375104 results.
47. AMED, EMBASE, Medline, CINAHL; 25 OR 26 OR 27 OR 28 OR 29 OR 30 OR 31 OR 32 OR 33 OR 34 OR 35 OR 36 OR 37 OR 38 OR 39 OR 40 OR 41 OR 42 OR 43 OR 44 OR 45 OR 46; 7833762 results.
48. AMED, EMBASE, Medline, CINAHL; squeeze*.ti,ab; 9290 results.
49. AMED, EMBASE, Medline, CINAHL; surg*.ti,ab; 3592311 results.
50. AMED, EMBASE, Medline, CINAHL; intervention*.ti,ab; 1719913 results.
51. AMED, EMBASE, Medline, CINAHL; irrigation*.ti,ab; 45442 results.
52. AMED, EMBASE, Medline, CINAHL; excis*.ti,ab; 326226 results.
53. AMED, EMBASE, Medline, CINAHL; infiltrat*.ti,ab; 404118 results.
54. AMED, EMBASE, Medline, CINAHL; therapeut*.ti,ab; 1901755 results.
55. AMED, EMBASE, Medline, CINAHL; manage*.ti,ab; 2431717 results.
56. AMED, EMBASE, Medline, CINAHL; treatment*.ti,ab; 8388485 results.

For peer review only

For peer review only - http://bmjopen.bmj.com/site/about/guidelines.xhtml

57. AMED, EMBASE, Medline, CINAHL; wash?out*.ti,ab; 28666 results.
58. AMED, EMBASE, Medline, CINAHL; operat*.ti,ab; 2000359 results.
59. AMED, EMBASE, Medline, CINAHL; bedside*.ti,ab; 57042 results.
60. AMED, EMBASE, Medline, CINAHL; liposuct*.ti,ab; 5719 results.
61. AMED, EMBASE, Medline, CINAHL; fasciotom*.ti,ab; 5310 results.
62. AMED, EMBASE, Medline, CINAHL; 48 OR 49 OR 50 OR 51 OR 52 OR 53 OR 54 OR 55 OR 56 OR 57 OR 58 OR 59 OR 60 OR 61; 15163731 results.
63. AMED, EMBASE, Medline, CINAHL; 9 AND 24; 222 results.
64. AMED, EMBASE, Medline, CINAHL; 9 AND 47; 982 results.
65. AMED, EMBASE, Medline, CINAHL; 9 AND 62; 1130 results.
66. AMED, EMBASE, Medline, CINAHL; 24 OR 47 OR 62; 20658308 results.
67. AMED, EMBASE, Medline, CINAHL; 9 AND 66; 1455 results.
68. AMED, EMBASE, Medline, CINAHL; 67 NOT animal; 1176 results.
69. AMED, EMBASE, Medline, CINAHL; 68 NOT laboratory; 1117 results.
70. AMED, EMBASE, Medline, CINAHL; 69 NOT adult; 804 results.

For peer review only

Evidence Services | library.nhs.uk

1. Thoracic and abdominal extravasation: a complication of hyperalimentation in infants.

Citation: AJR. American journal of roentgenology, Mar 1977, vol. 128, no. 3, p. 419-422, 0361-803X (March 1977)

Author(s): Spriggs, D W; Brantley, R E

Source: Medline

2. [Traumatic cerebral aneurysm showing remarkable extravasation into the lateral ventricle--report of a case in an infant (author's transl)].

Citation: No shinkei geka. Neurological surgery, Apr 1977, vol. 5, no. 4, p. 371-378, 0301-2603 (April 1977)

Author(s): Nakamura, T; Matsuoka, Y; Nishimura, S

Source: Medline

3. Urinary ascites in children owing to extravasation of urine from the bladder.

Citation: The Journal of urology, Sep 1979, vol. 122, no. 3, p. 409-411, 0022-5347 (September 1979)

Author(s): Redman, J F; Seibert, J J; Arnold, W

Source: Medline

4. Bone scintigraphy for neonatal osteomyelitis: simulation by extravasation of intravenous calcium.

Citation: Radiology, Apr 1980, vol. 135, no. 1, p. 185-186, 0033-8419 (April 1980)

Author(s): Balsam, D; Goldfarb, C R; Stringer, B; Farruggia, S

Source: Medline

5. Perirenal urinary extravasation associated with urethral valves in infants.

Citation: The Journal of urology, Nov 1980, vol. 124, no. 5, p. 688-691, 0022-5347 (November 1980)

Author(s): Mitchell, M E; Garrett, R A

Source: Medline

6. Nafcillin extravasation injury. Use of hyaluronidase as an antidote.

Citation: American journal of diseases of children (1960), Dec 1981, vol. 135, no. 12, p. 1113-1114, 0002-922X (December 1981)

Author(s): Zenk, K E; Dungy, C I; Greene, G R

Source: Medline

7. Cutaneous calcinosis in a neonate following extravasation of calcium gluconate.

Citation: Journal of the American Academy of Dermatology, Mar 1982, vol. 6, no. 3, p. 392-395, 0190-9622 (March 1982)

Author(s): Hironaga, M; Fujigaki, T; Tanaka, S

Source: Medline

8. Urinary extravasation in the newborn male with posterior urethral valves.

Citation: Journal of pediatric surgery, Dec 1982, vol. 17, no. 6, p. 751-756, 0022-3468 (December 1982)

Author(s): Greenfield, S P; Hensle, T W; Berdon, W E; Geringer, A M

For peer review only

For peer review only - http://bmjopen.bmj.com/site/about/guidelines.xhtml

Source: Medline

9. Intrathoracic extravasation of sclerosing agents associated with central venous catheters.

Citation: The American journal of pediatric hematology/oncology, Jan 1988, vol. 10, no. 3, p. 249-251, 0192-8562 (1988)

Author(s): Watterson, J; Heisel, M; Cich, J A; Priest, J R

Source: Medline

Full Text: Available from *Ovid* in *American Journal of Pediatric Hematology/Oncology*

10. Nonoperative management of full-thickness intravenous extravasation injuries in premature neonates using enzymatic debridement.

Citation: Annals of plastic surgery, Feb 1989, vol. 22, no. 2, p. 146-149, 0148-7043 (February 1989)

Author(s): Falcone, P A; Barrall, D T; Jeyarajah, D R; Grossman, J A

Source: Medline

Full Text: Available from *Ovid* in *Annals of Plastic Surgery*

For peer review only

Search Results

Table of Contents

Search History .... page 2

1. Hepatic and splenic blush on computed tomography in children following blunt abdominal trauma: Is intervention necessary? .... page 3

2. Safety and effectiveness of intravenous pentamidine for prophylaxis of Pneumocystis jirovecii pneumonia in pediatric hematology/oncology patients .... page 3

3. Efficacy, Safety, and Pharmacokinetics of a New 10 % Liquid Intravenous Immunoglobulin Containing High Titer Neutralizing Antibody to RSV and Other Respiratory Viruses in Subjects with Primary Immunodeficiency Disease .... page 3

4. A multicenter, randomized, observer-blinded, active-controlled study evaluating the safety and effectiveness of ceftaroline compared with ceftriaxone plus vancomycin in pediatric patients with complicated community-acquired bacterial pneumonia .... page 3

5. Predictive factors for conservative treatment failure in grade IV pediatric blunt renal trauma .... page 3

6. Clinical negligence claims in pediatric surgery in England: Pattern and trends .... page 3

7. Outcomes of peripherally inserted double lumen central catheter in very low birth weight infants .... page 4

8. Pelvic Artery Embolization in the Management of Obstetrical Hemorrhage: Predictive Factors for Clinical Outcomes .... page 4

9. The role of interventional radiology for pediatric blunt renal trauma .... page 4

10. Safety and efficacy of gadoteric acid in pediatric magnetic resonance imaging: overview of clinical trials and post-marketing studies .... page 4

For peer review only

For peer review only - http://bmjopen.bmj.com/site/about/guidelines.xhtml

Search History

1. EMBASE; exp PEDIATRICS/; 87850 results.
2. EMBASE; exp CHILD/; 2256483 results.
3. EMBASE; exp INFANT/; 930470 results.
4. EMBASE; exp ADOLESCENT/; 1367630 results.
5. EMBASE; exp NEWBORN/; 495069 results.
6. EMBASE; exp CONTRAST MEDIUM EXTRAVASATION/ OR exp DRUG EXTRAVASATION/ OR exp EXTRAVASATION/ OR exp INJECTION SITE EXTRAVASATION/; 11979 results.
7. EMBASE; exp OUTCOME MEASUREMENT/ OR exp OUTCOMES RESEARCH/ OR exp EVIDENCE BASED MEDICINE/ OR exp TREATMENT OUTCOME/; 1809683 results.
8. EMBASE; 1 OR 2 OR 3 OR 4 OR 5; 2973897 results.
9. EMBASE; 6 AND 8; 958 results.
10. EMBASE; 7 AND 9; 150 results.

For peer review only

For peer review only - http://bmjopen.bmj.com/site/about/guidelines.xhtml

Evidence Services | library.nhs.uk

1. Hepatic and splenic blush on computed tomography in children following blunt abdominal trauma: Is intervention necessary?

**Citation:**	Journal of Trauma and Acute Care Surgery, 2016, vol./is. 81/2(266-270), 2163-0755;2163-0763 (2016)
**Author(s):**	Ingram M.-C.E.; Siddharthan R.V.; Morris A.D.; Hill S.J.; Travers C.D.; McKracken C.E.; Heiss K.F.; Raval M.V.; Santore M.T.
**Source:**	EMBASE
**Full Text:**	Available from *Ovid* in *Journal of Trauma and Acute Care Surgery*

2. Safety and effectiveness of intravenous pentamidine for prophylaxis of Pneumocystis jirovecii pneumonia in pediatric hematology/oncology patients

**Citation:**	Journal of Pediatric Hematology/Oncology, July 2016, vol./is. 38/6(e180-e185), 1077-4114;1536-3678 (26 Jul 2016)
**Author(s):**	Solodokin L.J.; Klejmont L.M.; Scipione M.R.; Dubrovskaya Y.; Lighter-Fisher J.; Papadopoulos J.
**Source:**	EMBASE
**Full Text:**	Available from *Ovid* in *Journal of Pediatric Hematology/Oncology*

3. Efficacy, Safety, and Pharmacokinetics of a New 10 % Liquid Intravenous Immunoglobulin Containing High Titer Neutralizing Antibody to RSV and Other Respiratory Viruses in Subjects with Primary Immunodeficiency Disease

**Citation:**	Journal of Clinical Immunology, August 2016, vol./is. 36/6(590-599), 0271-9142;1573-2592 (01 Aug 2016)
**Author(s):**	Wasserman R.L.; Lumry W.; Harris J.; Levy R.; Stein M.; Forbes L.; Cunningham-Rundles C.; Melamed I.; Kobayashi A.L.; Du W.; Kobayashi R.
**Source:**	EMBASE
**Full Text:**	Available from *Springer Link Journals* in *Journal of Clinical Immunology*

4. A multicenter, randomized, observer-blinded, active-controlled study evaluating the safety and effectiveness of ceftaroline compared with ceftriaxone plus vancomycin in pediatric patients with complicated community-acquired bacterial pneumonia

**Citation:**	Pediatric Infectious Disease Journal, July 2016, vol./is. 35/7(760-766), 0891-3668;1532-0987 (01 Jul 2016)
**Author(s):**	Blumer J.L.; Ghonghadze T.; Cannavino C.; O'Neal T.; Jandourek A.; Friedland H.D.; Bradley J.S.
**Source:**	EMBASE
**Full Text:**	Available from *Ingenta* in *Pediatric Infectious Disease Journal, The*<br>Available from *Ovid* in *Pediatric Infectious Disease Journal*

5. Predictive factors for conservative treatment failure in grade IV pediatric blunt renal trauma

**Citation:**	Journal of Pediatric Urology, April 2016, vol./is. 12/2(93.e1-93.e7), 1477-5131;1873-4898 (01 Apr 2016)
**Author(s):**	Lee J.N.; Lim J.K.; Woo M.J.; Kwon S.Y.; Kim B.S.; Kim H.T.; Kim T.-H.; Yoo E.S.; Chung S.K.
**Source:**	EMBASE

6. Clinical negligence claims in pediatric surgery in England: Pattern and trends

**Citation:**	European Journal of Pediatric Surgery, February 2015, vol./is. 25/1(66-70), 0939-7248;1439-359X (February 2015)

For peer review only

Author(s): Thyoka M.

Source: EMBASE

7. Outcomes of peripherally inserted double lumen central catheter in very low birth weight infants

Citation: Journal of Neonatal-Perinatal Medicine, March 2016, vol./is. 9/1(99-105), 1934-5798;1878-4429 (24 Mar 2016)

Author(s): Cheong S.M.; Totsu S.; Nakanishi H.; Uchiyama A.; Kusuda S.

Source: EMBASE

8. Pelvic Artery Embolization in the Management of Obstetrical Hemorrhage: Predictive Factors for Clinical Outcomes

Citation: CardioVascular and Interventional Radiology, December 2015, vol./is. 38/6(1477-1486), 0174-1551;1432-086X (01 Dec 2015)

Author(s): Zhang E.; Liu L.; Owen R.

Source: EMBASE

Full Text: Available from *Springer Link Journals* in *CardioVascular and Interventional Radiology*

9. The role of interventional radiology for pediatric blunt renal trauma

Citation: Italian Journal of Pediatrics, October 2015, vol./is. 41/1(no pagination), 1720-8424;1824-7288 (15 Oct 2015)

Author(s): Lin W.-C.; Lin C.-H.

Source: EMBASE

Full Text: Available from *National Library of Medicine* in *Italian Journal of Pediatrics*
Available from *BioMed Central* in *Italian Journal of Pediatrics*
Available from *National Library of Medicine* in *Italian Journal of Pediatrics*
Available from *ProQuest* in *Italian Journal of Pediatrics*

10. Safety and efficacy of gadoteric acid in pediatric magnetic resonance imaging: overview of clinical trials and post-marketing studies

Citation: Pediatric Radiology, November 2015, vol./is. 45/12(1831-1841), 0301-0449;1432-1998 (01 Nov 2015)

Author(s): Balassy C.; Roberts D.; Miller S.F.

Source: EMBASE

Full Text: Available from *Springer Link Journals* in *Pediatric Radiology*

For peer review only

Search Results

Table of Contents

Search History .... page 2

1. Emergency treatment of accidental infusion leakage in the newborn. .... page 3

2. Childhood extravasation injuries: improved outcome following the introduction of hospital-wide guidelines. .... page 3

3. [Treatment protocol for extravasation lesions]. .... page 3

4. The use of central venous catheters in paediatric oncology--a cautionary tale. .... page 3

5. A new approach to management of intravenous infiltration in pediatric patients: pathophysiology, classification, and treatment. .... page 3

6. Saline irrigation for the management of skin extravasation injury in neonates. .... page 3

7. Extravasation injuries. .... page 3

8. [Central venous catheters in newborn infants. Work Group Parenteral Nutrition of the Perinatology Section of the Dutch Pediatric Society]. .... page 4

9. Extravasation of parenteral alimentation fluid into the renal pelvis--a complication of central venous catheter in a neonate. .... page 4

10. Percutaneous central line extravasation masquerading as an abscess. .... page 4

For peer review only

For peer review only - http://bmjopen.bmj.com/site/about/guidelines.xhtml

Search History

1. Medline; exp PEDIATRICS/; 49428 results.
2. Medline; exp CHILD/; 1666146 results.
3. Medline; exp INFANT/; 1008023 results.
4. Medline; exp INFANT, NEWBORN/; 535610 results.
5. Medline; 1 OR 2 OR 3 OR 4; 2185064 results.
6. Medline; exp EXTRAVASATION OF DIAGNOSTIC AND THERAPEUTIC MATERIALS/; 3098 results.
7. Medline; exp "OUTCOME ASSESSMENT (HEALTH CARE)"/ OR exp PATIENT OUTCOME ASSESSMENT/ OR exp TREATMENT OUTCOME/; 815946 results.
8. Medline; exp EARLY MEDICAL INTERVENTION/; 1559 results.
9. Medline; exp THERAPEUTIC IRRIGATION/ OR exp THERAPEUTICS/; 5011881 results.
10. Medline; exp SPECIALTIES, SURGICAL/ OR exp SURGICAL PROCEDURES, OPERATIVE/; 2809893 results.
11. Medline; 8 OR 9 OR 10; 6354238 results.
12. Medline; 5 AND 6; 345 results.
13. Medline; 7 AND 12; 36 results.
14. Medline; 11 AND 12; 248 results.
15. Medline; 12 OR 13 OR 14; 345 results.

For peer review only

For peer review only - http://bmjopen.bmj.com/site/about/guidelines.xhtml

1. Emergency treatment of accidental infusion leakage in the newborn.

Citation: British journal of plastic surgery, Jan 2002, vol. 55, no. 1, p. 89., 0007-1226 (January 2002)

Author(s): Rose, Victoria; Turner, Matthew; Harris, Paul A; Moss, Anthony L H

Source: Medline

Full Text: Available from *Ovid* in *Plastic and Reconstructive Surgery*

2. Childhood extravasation injuries: improved outcome following the introduction of hospital-wide guidelines.

Citation: Journal of plastic, reconstructive & aesthetic surgery : JPRAS, Apr 2015, vol. 68, no. 4, p. 505-518, 1878-0539 (April 2015)

Author(s): Ghanem, Ali M; Mansour, Abdulrab; Exton, Rebecca; Powell, Jonathan; Mashhadi, Syed; Bulstrode, Neil; Smith, Gillian

Source: Medline

3. [Treatment protocol for extravasation lesions].

Citation: Cirugía pediátrica : organo oficial de la Sociedad Española de Cirugía Pediátrica, Jul 2006, vol. 19, no. 3, p. 136-139, 0214-1221 (July 2006)

Author(s): Andrés, A M; Burgos, L; López Gutiérrez, J C; Encinas, J L; Díaz, M; Rivas, S; Ros, Z

Source: Medline

4. The use of central venous catheters in paediatric oncology--a cautionary tale.

Citation: Scottish medical journal, Feb 1990, vol. 35, no. 1, p. 11-14, 0036-9330 (February 1990)

Author(s): Russell, L; Craig, J I; Mackinlay, G A; Eden, O B

Source: Medline

5. A new approach to management of intravenous infiltration in pediatric patients: pathophysiology, classification, and treatment.

Citation: Journal of infusion nursing : the official publication of the Infusion Nurses Society, Jul 2011, vol. 34, no. 4, p. 242-249, 1539-0667 (2011 Jul-Aug)

Author(s): Amjad, Ibrahim; Murphy, Travis; Nylander-Housholder, Linda; Ranft, Amanda

Source: Medline

Full Text: Available from *Ovid* in *Journal of Infusion Nursing*

6. Saline irrigation for the management of skin extravasation injury in neonates.

Citation: The Cochrane database of systematic reviews, Jan 2012, no. 2, p. CD008404., 1469-493X (2012)

Author(s): Gopalakrishnan, P N; Goel, N; Banerjee, Sujoy

Source: Medline

Full Text: Available from *John Wiley and Sons* in *Cochrane Library, The*

7. Extravasation injuries.

Citation: British journal of plastic surgery, Mar 1993, vol. 46, no. 2, p. 91-96, 0007-1226 (March 1993)

Author(s): Gault, D T

Source: Medline

Full Text: Available from *Ovid* in *Plastic and Reconstructive Surgery*

For peer review only

8. [Central venous catheters in newborn infants. Work Group Parenteral Nutrition of the Perinatology Section of the Dutch Pediatric Society].

**Citation:**	Tijdschrift voor kindergeneeskunde, Jun 1993, vol. 61, no. 3, p. 76-82, 0376-7442 (June 1993)
**Author(s):**	van Lingen, R A; Liem, K D; Krediet, T G
**Source:**	Medline

9. Extravasation of parenteral alimentation fluid into the renal pelvis--a complication of central venous catheter in a neonate.

**Citation:**	Journal of perinatology : official journal of the California Perinatal Association, Oct 2001, vol. 21, no. 7, p. 465-466, 0743-8346 (2001 Oct-Nov)
**Author(s):**	Nadroo, A M; al-Sowailem, A M
**Source:**	Medline
**Full Text:**	Available from *Nature Publishing Group* in *Journal of Perinatology*<br>Available from *Nature Publishing Group* in *Journal of Perinatology*<br>Available from *ProQuest* in *Journal of Perinatology*

10. Percutaneous central line extravasation masquerading as an abscess.

**Citation:**	Indian pediatrics, Apr 2014, vol. 51, no. 4, p. 309-310, 0974-7559 (April 2014)
**Author(s):**	Govind, Binu; Tete, Prakash Ignace; Thomas, Niranjan
**Source:**	Medline
**Full Text:**	Available from *Springer Link Journals* in *Indian Pediatrics*

For peer review only

Supplementary 2
Study Attrition Process

Level 1 - Is a clinical study of paediatric extravasation injury?

If yes then proceed to level 2

Level 2 - Does it report on one or more of the following outcomes?

- Short-term outcomes:
    - Time to complete tissue healing
    - Infection
    - Hypothermia
    - Anaphylactic reactions
    - Fluid and electrolyte disturbance
    - Any other adverse effects reported in the studies;
- Long-term outcomes (preferably at least three months from the injury):
    - Severity of the scar including contractures, functional impairment, disfigurement and need for further surgical procedures.

Level 3 – is it an original clinical study or systematic review of clinical studies?

If yes then proceed to level 4

Level 4 - In which of the following groups of the study types does it belong?

Group 1

- RCT
- Quasi-randomised study (e.g. alternation rather than randomisation used)
- Systematic reviews of RCTs or quasi-randomised studies

Group 2

- Cross-sectional study
- Case-series
- Systematic reviews of cross-sectional studies or case-series

If yes then retrieve full article for consideration for inclusion

For peer review only

Supplementary 3
Review-Specific Proforma

Author	Patients (n)	Age (mean)	Gender		Comorbidities				Cannula location			Cannula type	
			M	F	Premature	Sepsis	Malignancy	Other	Upper limb	Lower limb	Scalp	Peripheral	Central

Author	Vesicants							Medical Management	Surgical Management	Control (Y/N)
	TPN	≥5% Dextrose	Other IV Fluids	Calcium-containing fluids	Antibiotics	Contrast	Other			

Author	Surgical injuries								Wound outcome	Functional outcome
	Swelling/ Erythema	Partial thickness skin injury	Full thickness skin injury	Tendon injury	Nerve injury	Vascular injury	Compartment Syndrome	Amputation		

For peer review only

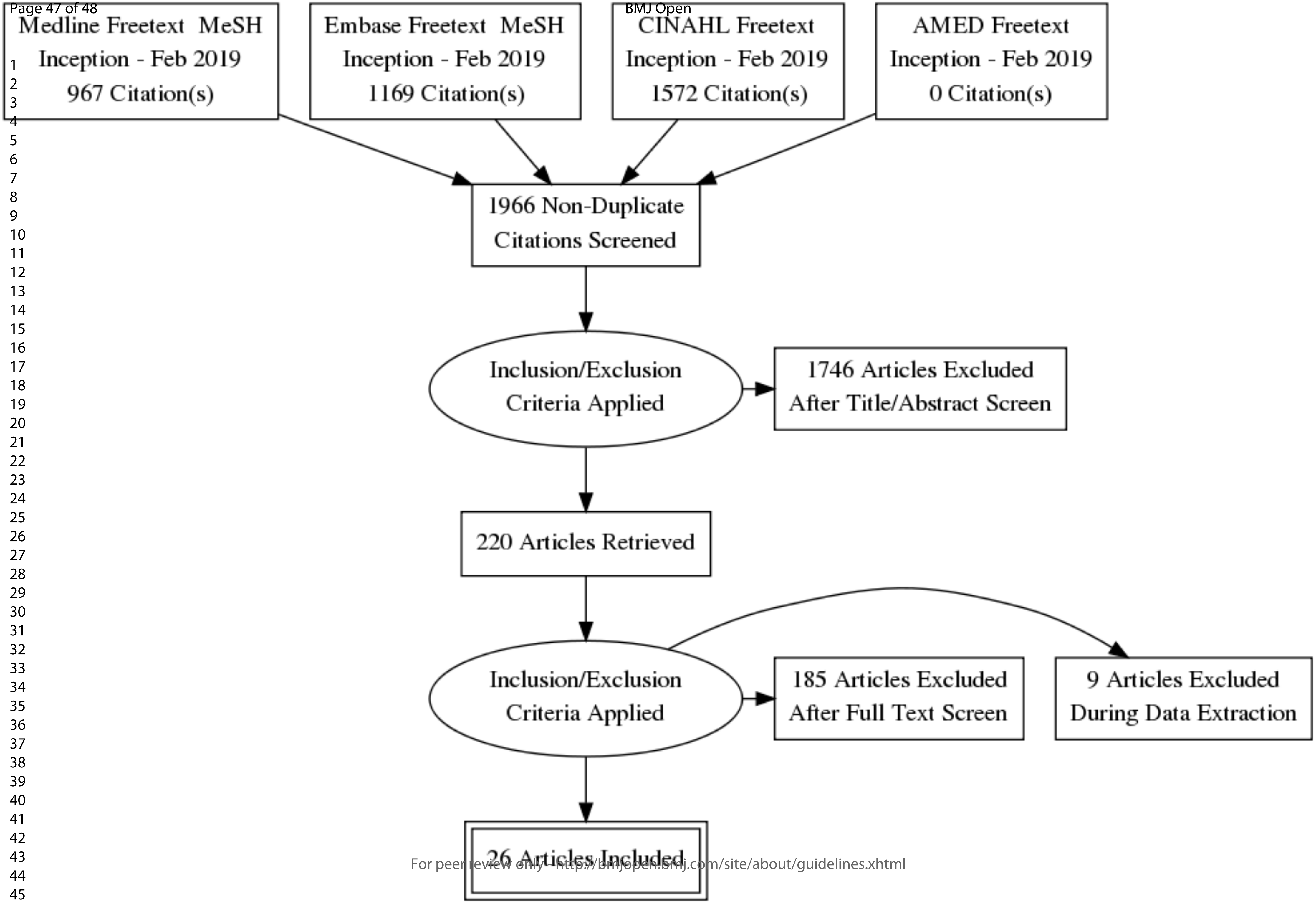
Medline Freetext MeSH
Inception - Feb 2019
967 Citation(s)
Embase Freetext MeSH
Inception - Feb 2019
1169 Citation(s)
CINAHL Freetext
Inception - Feb 2019
1572 Citation(s)
AMED Freetext
Inception - Feb 2019
0 Citation(s)
1966 Non-Duplicate Citations Screened
Inclusion/Exclusion Criteria Applied
1746 Articles Excluded After Title/Abstract Screen
220 Articles Retrieved
Inclusion/Exclusion Criteria Applied
185 Articles Excluded After Full Text Screen
9 Articles Excluded During Data Extraction
26 Articles Included

PRISMA 2009 Checklist – Surgical Intervention for Paediatric Extravasation

Section/topic	#	Checklist item	Reported on page #
**TITLE**			
Title	1	Identify the report as a systematic review, meta-analysis, or both.	1
**ABSTRACT**			
Structured summary	2	Provide a structured summary including, as applicable: background; objectives; data sources; study eligibility criteria, participants, and interventions; study appraisal and synthesis methods; results; limitations; conclusions and implications of key findings; systematic review registration number.	2
**INTRODUCTION**			
Rationale	3	Describe the rationale for the review in the context of what is already known.	4
Objectives	4	Provide an explicit statement of questions being addressed with reference to participants, interventions, comparisons, outcomes, and study design (PICOS).	5
**METHODS**			
Protocol and registration	5	Indicate if a review protocol exists, if and where it can be accessed (e.g., Web address), and, if available, provide registration information including registration number.	6
Eligibility criteria	6	Specify study characteristics (e.g., PICOS, length of follow-up) and report characteristics (e.g., years considered, language, publication status) used as criteria for eligibility, giving rationale.	6
Information sources	7	Describe all information sources (e.g., databases with dates of coverage, contact with study authors to identify additional studies) in the search and date last searched.	6,7,8
Search	8	Present full electronic search strategy for at least one database, including any limits used, such that it could be repeated.	Supplementary-1
Study selection	9	State the process for selecting studies (i.e., screening, eligibility, included in systematic review, and, if applicable, included in the meta-analysis).	7
Data collection process	10	Describe method of data extraction from reports (e.g., piloted forms, independently, in duplicate) and any processes for obtaining and confirming data from investigators.	Supplementary-2
Data items	11	List and define all variables for which data were sought (e.g., PICOS, funding sources) and any assumptions and simplifications made.	Supplementary-2
Risk of bias in individual studies	12	Describe methods used for assessing risk of bias of individual studies (including specification of whether this was done at the study or outcome level), and how this information is to be used in any data synthesis.	7
Summary measures	13	State the principal summary measures (e.g., risk ratio, difference in means).	N/A
Synthesis of results	14	Describe the methods of handling data and combining results of studies, if done, including measures of consistency (e.g., I^2) for each meta-analysis.	N/A

For peer review only - http://bmjopen.bmj.com/site/about/guidelines.xhtml

PRISMA 2009 Checklist – Surgical Intervention for Paediatric Extravasation

Section/topic	#	Checklist item	Reported on page #
Risk of bias across studies	15	Specify any assessment of risk of bias that may affect the cumulative evidence (e.g., publication bias, selective reporting within studies).	N/A
Additional analyses	16	Describe methods of additional analyses (e.g., sensitivity or subgroup analyses, meta-regression), if done, indicating which were pre-specified.	N/A
**RESULTS**			
Study selection	17	Give numbers of studies screened, assessed for eligibility, and included in the review, with reasons for exclusions at each stage, ideally with a flow diagram.	8
Study characteristics	18	For each study, present characteristics for which data were extracted (e.g., study size, PICOS, follow-up period) and provide the citations.	Table 1 + Table 2
Risk of bias within studies	19	Present data on risk of bias of each study and, if available, any outcome level assessment (see item 12).	Table 5
Results of individual studies	20	For all outcomes considered (benefits or harms), present, for each study: (a) simple summary data for each intervention group (b) effect estimates and confidence intervals, ideally with a forest plot.	Table 4
Synthesis of results	21	Present results of each meta-analysis done, including confidence intervals and measures of consistency.	N/A
Risk of bias across studies	22	Present results of any assessment of risk of bias across studies (see Item 15).	N/A
Additional analysis	23	Give results of additional analyses, if done (e.g., sensitivity or subgroup analyses, meta-regression [see Item 16]).	N/A
**DISCUSSION**			
Summary of evidence	24	Summarize the main findings including the strength of evidence for each main outcome; consider their relevance to key groups (e.g., healthcare providers, users, and policy makers).	14-17
Limitations	25	Discuss limitations at study and outcome level (e.g., risk of bias), and at review-level (e.g., incomplete retrieval of identified research, reporting bias).	13
Conclusions	26	Provide a general interpretation of the results in the context of other evidence, and implications for future research.	19
**FUNDING**			
Funding	27	Describe sources of funding for the systematic review and other support (e.g., supply of data); role of funders for the systematic review.	1

From: Moher D, Liberati A, Tetzlaff J, Altman DG, The PRISMA Group (2009). Preferred Reporting Items for Systematic Reviews and Meta-Analyses: The PRISMA Statement. PLoS Med 6(6): e1000097. doi:10.1371/journal.pmed1000097

For more information, visit: **www.prisma-statement.org**.

For peer review only - http://bmjopen.bmj.com/site/about/guidelines.xhtml
